# Revision, cladistic analysis and biogeography of *Typhochlaena* C. L. Koch, 1850, *Pachistopelma* Pocock, 1901 and *Iridopelma* Pocock, 1901 (Araneae, Theraphosidae, Aviculariinae)

**DOI:** 10.3897/zookeys.230.3500

**Published:** 2012-10-23

**Authors:** Rogério Bertani

**Affiliations:** 1 Instituto Butantan, Laboratório Especial de Ecologia e Evolução, Av. Vital Brazil, 1500, 05503–900, São Paulo – SP, Brazil

**Keywords:** Brazilian Atlantic rainforest, bromeliads, campo rupestre, cerrado, endemism, new species, restinga, systematics, tarantula

## Abstract

Three aviculariine genera endemic to Brazil are revised. *Typhochlaena* C. L. Koch, 1850 is resurrected, including five species; *Pachistopelma* Pocock, 1901 includes two species; and *Iridopelma* Pocock, 1901, six species. Nine species are newly described: *Typhochlaena amma* **sp. n.**, *Typhochlaena costae* **sp. n.**, *Typhochlaena curumim* **sp. n.**, *Typhochlaena paschoali* **sp. n.**, *Pachistopelma bromelicola* **sp. n.**, *Iridopelma katiae* **sp. n.**, *Iridopelma marcoi* **sp. n.**, *Iridopelma oliveirai* **sp. n.** and *Iridopelma vanini* **sp. n.** Three new synonymies are established: *Avicularia pulchra* Mello-Leitão, 1933 and *Avicularia recifiensis* Struchen & Brändle, 1996 are junior synonyms of *Pachistopelma rufonigrum* Pocock, 1901 **syn. n.**, and *Avicularia palmicola* Mello-Leitão, 1945 is a junior synonym of *Iridopelma hirsutum* Pocock, 1901 **syn. n.** *Pachistopelma concolor* Caporiacco, 1947 is transferred to *Tapinauchenius* Ausserer, 1871, making the new combination *Tapinauchenius concolor* (Caporiacco, 1947*)* **comb. n.** Lectotypes are newly designed for *Pachistopelma rufonigrum* Pocock, 1901 , *Iridopelma hirsutum* Pocock, 1901 and *Pachistopelma concolor* Caporiacco, 1947. Cladistic analyses using both equal and implied weights were carried out with a matrix comprising 62 characters and 38 terminal taxa. The chosen cladogram found with X-Pee-Wee and concavity 6 suggests they are monophyletic. All species are keyed and mapped and information on species habitat and area cladograms are presented. Discussion on biogeography and conservation is provided.

## Introduction

Theraphosidae is the richest mygalomorph family, comprising *ca*. 1/3 of the 2,693 known mygalomorph species (Platnick 2012). The New World is particularly rich in theraphosid species (Platnick 2012), comprising representatives of three subfamilies: Ischnocolinae, Theraphosinae and Aviculariinae which comprises more than a half of the 932 known theraphosid species. Despite their potential importance as top predators in ecological webs (Sergio et al. 2008), the pet trade (e. g. Yáñez and Floater 2000, Bertani and Fukushima 2009) and a source of important tools for pharmacological research and therapeutical leads (e. g. Escoubas and Rash 2004), theraphosids have been largely neglected in taxonomical and biological research. Only in the past 30 years have they received more attention from the scientific community in some taxonomic revisions and cladistic analyses (e. g. Raven 1985, Pérez-Miles et al. 1996, Bertani 2001, West et al. 2008, West et al. 2012), molecular analyses (e. g. Hamilton et al. 2011), biochemical and pharmaceutical (e. g. Silva et al. 2000, Bode et al. 2001, Escoubas and Rash 2004), ethological and ecological studies (e. g. Marshall and Uetz 1990, Bertani and Marques 1996, Yáñez and Floater 2000) that have displayed a fascinating diversity in all these aspects.

This work focuses on those aviculariines occurring mainly in Northeastern Brazil from where the type species of aviculariine genera *Typhochlaena* C. L. Koch, 1850, *Pachistopelma* Pocock, 1901 and *Iridopelma* Pocock, 1901 were described. Within all aviculariine genera presently accepted (West et al. 2008), (*Avicularia* Lamarck, 1818, *Iridopelma*, *Pachistopelma*, *Ephebopus* Simon, 1892, *Tapinauchenius* Ausserer, 1871, *Psalmopoeus* Pocock, 1895 from the New World – and *Stromatopelma* Karsch, 1881 and *Heteroscodra* Pocock, 1889 – from Africa), these three are the least understood both taxonomically and biologically.

*Typhochlaena* and *Iridopelma* share a history of synonymies and revalidations, whereas *Pachistopelma* has lacked important taxonomic change since its description. *Typhochlaena* was erected by C. L. Koch (1850) for his species *Mygale seladonia* C. L. Koch, 1841 from Bahia, Brazil, and *Mygale caesia* C. L. Koch, 1842, from Puerto Rico. Only supposed females were described and the diagnosis for the genus was very short, with references to the highly colored abdomen and well developed scopulae on the legs. Karsch (1879) described *Typhochlaena magdalenae* Karsch, 1879, male, from Santha-Martha, Colombia, but did not discuss the genus diagnosis.

Simon (1892) synonymized *Typhochlaena* with *Avicularia*, and considered the small species with mottled abdomen to belong in a distinct group of *Avicularia* species: *Avicularia seladonia*, *Avicularia caesia*, *Avicularia magdalenae* and *Avicularia glauca* Simon, 1891. Simon (1892) recognized only two genera in Avicularieae: *Tapinauchenius* and *Avicularia*. However, Pocock (1901) considered that *Psalmopoeus* and *Ephebopus* should also be included in Simon’s Avicularieae Simon, 1892 to which he also added two new genera, *Pachistopelma* Pocock, 1901 and *Iridopelma* Pocock, 1901. *Pachistopelma* was described utilizing the male and female of a single species, *Pachistopelma rufonigrum* Pocock, 1901 from Igarassu, Brazil. Pocock (1901) considered it closely related to *Avicularia* due to a single spinose spur on the tibia of first leg in males, marginal sternal sigilla, absence of spines and development of scopulae, and differentiated it by the slightly procurved first ocular row in *Pachistopelma*. *Iridopelma* Pocock, 1901 was described in the same paper also using the male and female of a single species, *Iridopelma hirsutum* Pocock, 1901, from Pernambuco, Brazil. Pocock (1901) also related this genus with *Avicularia*, but differentiated it by the presence in *Iridopelma* of a spur on the tibia of leg II in males and leg I being longer than leg IV.

Two years later, Simon (1903) synonymized *Iridopelma* with *Avicularia*, by considering the presence of a tibial spur on leg II of the male to be of little generic significance and the females lack characteristics that allow generic recognition. Mello-Leitão (1923) disagreed with Simon (1903) and considered it possible to identify females of *Iridopelma* by characters given by Pocock (1901), i. e. leg I longer than IV and size of the median anterior eyes. However, he received a male from the state of Bahia, Brazil with tibial spurs on legs I and II and identified it, with doubts, as *Typhochlaena seladonia*, thus synonymizing *Iridopelma* with *Typhochlaena* (Mello-Leitão 1923). He stated that, in the case of this specimen not being congeneric with *Typhochlaena seladonia*, it should be considered a new *Iridopelma* species, named *Iridopelma amphidorotherion* Mello-Leitão, 1923. The generic description given by him for the genus *Iridopelma* should be seen as the description of that species (Mello-Leitão 1923). In the same paper (and page) it was given a new name, *Typhochlaena pococki* Mello-Leitão, 1923 for *Iridopelma hirsutum* Pocock, 1901, without further explanation. Another species, *Typhochlaena zorodes* Mello-Leitão, 1926 was described three years later from a male from the state of Bahia, Brazil, and distinguished from other species by the uniform abdominal coloration, cephalothorax as wide as long and anterior eyes of equal size (Mello-Leitão 1926).

Roewer’s (1942) Catalog listed both *Iridopelma* and *Typhochlaena* as junior-synonyms of *Avicularia*. Raven (1985) revalidated *Iridopelma*, and Smith (1993) redescribed the types of *Iridopelma hirsutum* and transferred from *Avicularia Iridopelma zorodes* (Mello-Leitão, 1926) and *Iridopelma seladonium* (C. L. Koch, 1841), making new combinations. Almeida-Silva et al. (2008) obtained photographs of the holotype and described the male of *Iridopelma seladonium* for the first time and retained the species in *Iridopelma*, even though the male lacks tibial spurs both on leg I and II, the diagnostic character of the genus (Pocock 1901, Raven 1985). Concerning *Pachistopelma*, Caporiacco (1947) described a new species, *Pachistopelma concolor* Caporiacco, 1947, from Guyana. To date, this genus has remained unrevised.

Herein, species of *Typhochlaena*, *Pachistopelma* and *Iridopelma* are revised and relationships are analyzed using cladistic methods. Geographical distribution and notes on habitats of species are given with area cladograms also presented. Thirteen species are herein recognized, five in *Typhochlaena*, six in *Iridopelma* and two in *Pachistopelma*, nine of which are newly described, showing an unexpected high richness of aviculariine species out of their core distribution within and immediately surrounding the Amazon region.

## Methods

The general description format follows Raven (2005) with some modifications, e. g., hair types and trichobothrial conformation on legs were not as deeply studied as in that work. Terminology of urticating hairs follows Cooke et al. (1972). All measurements are in millimeters and were obtained with a Mitutoyo digital caliper with an error of 0.005 mm, rounded up to two significant decimals. Leg and palp measurements were taken from the dorsal aspect of the left side (unless appendages were lost or obviously regenerated). A Nikon SMZ1500 dissecting microscope was used for illustrations (with a camera lucida attachment). Abbreviations: ALE = anterior lateral eyes, AME = anterior median eyes, ITC = inferior tarsal claw, PLE = posterior lateral eyes, PME = posterior median eyes, PMS = posterior median spinnerets, STC = superior tarsal claws, AMNRJ = Arachne collecting team from MNRJ (Arachnids and Myriapods from Atlantic Rain Forest Project).

Specimens from the following institutions were examined: DZUB – Departamento de Zoologia da Universidade de Brasília, Brasilia; IBSP – Instituto Butantan, São Paulo; MIZA – Instituto de Zoologia, Universidad Central de Venezuela, Maracay; MNRJ – Museu Nacional do Rio de Janeiro, Rio de Janeiro; MPEG – Museu Paraense Emilio Goeldi, Belém; MZSP – Museu de Zoologia da Universidade de São Paulo, São Paulo; MZUF – Museo Zoologico “La Specola”, Firenze; NHM – The Natural History Museum, London; SMF – Senckenberg Museum, Frankfurt.

Geographical coordinates: primary sources are between round brackets and secondary sources (Google Earth©) are between square brackets (following West et al. 2008).

Cladistic analyses were based mainly on the matrix of West et al. (2008), except for characters 7, 9, 10, 12, 13, 28–30, 33–35, 39, 40, 49, 59, 61, newly introduced or modified. Representatives of seven of the eight theraphosid subfamilies recognized by Raven (1985) were included, as well as the genera *Poecilotheria* Simon, 1885 and *Encyocratella* Strand, 1907, of uncertain taxonomic position. Following West et al. (2008), an undescribed species of *Melloina* Brignoli, 1985 (Paratropididae, Glabropelmatinae) was included as the outgroup. It was chosen because paratropidids are the sister-group of Theraphosidae and *Melloina* is the genus which retains more plesiomorphic characters in common with the Theraphosidae (Raven 1985).

## Data resources

Matrix data deposited in the Dryad repository: doi: 10.5061/dryad.bv0kr

## Examined specimens for cladistics

For *Iridopelma*, *Pachistopelma* and *Typhochlaena* species, it was used the same specimens used in descriptions.

**Paratropididae:** Glabropelmatinae. *Melloina* sp., male (MIZA 520), female (MNRJ 12961), Venezuela, Lara, Cueva El Santuario [9°49'N, 70°03'W], O. Villarreal, 19 April 2000.

**Theraphosidae:** Aviculariinae: *Avicularia* sp. male, Brazil, state of Pará, U.H.E. Tucuruí [4°20'S, 49°31'W], Equipe Resgate de Fauna, 1984 (IBSP 7879 IBA 1002), female, same locality and collectors (IBSP 8845 Ref 48186); *Avicularia diversipes* (C. L. Koch, 1842), female, Brazil, state of Bahia, Ilhéus, CEPLAC [14°46'S, 39°13'W], R. Bertani and G. Puorto, March 1991 (IBSP 11754), male, same data (IBSP 119271); *Avicularia gamba* Bertani and Fukushima, 2009, holotype male, Brazil, state of Bahia, Elísio Medrado, RPPN Jequitibá (12°52'3.20"S, 39°28'9.09"W), R. Bertani, C.S. Fukushima and R.H. Nagahama, 07 October 2007 (MZSP 31115), paratype female, same data (MZSP 31116); *Avicularia juruensis* Mello-Leitão, 1923, male, Brazil, state of Rondônia, Porto Velho [8°45'S, 63°54'W] (IBSP 2503), female, Brazil, state of Mato Grosso, between Vale de São Lourenço and Pontes e Lacerda, U.H.E. Guaporé [14°40'S, 56°51'W] (IBSP 10279); *Avicularia sooretama* Bertani and Fukushima, 2009, holotype male, Brazil, state of Espírito Santo, Reserva Biológica de Sooretama [18°59'S, 40°07'W], AMNRJ, 18 April 2006 (MNRJ 18435), paratype female, Brazil, state of Espírito Santo, Pinheiros, Reserva Biológica Córrego do Veado (18°37'0.16"S, 40°14'1.60"W), AMNRJ, 22 October 2005 (MNRJ 12930); *Avicularia taunayi* (Mello-Leitão, 1920), female, Brazil, state of Minas Gerais, Barão de Cocais [19°56'S, 43°28'W], J. P. Couto, 5 November 1970 (IBSP 199), male, Brazil, Distrito Federal, Brasília, Lago Sul [15°50'S, 47°49'W], R.A. Brandão and G. Zerbini (DZUB 1675); *Ephebopus murinus* (Walckenaer, 1837), male (IBSP 9650), female (IBSP 9658), Brazil, state of Pará, Tucuruí, U.H.E. Tucuruí [4°20'S, 49°31'W], Equipe de Resgate de Fauna, 1984; *Ephebopus uatuman* Lucas, Silva & Bertani, 1992, male holotype (IBSP 4939), female paratype (IBSP 4940), Brazil, state of Amazonas, Presidente Figueiredo, Uatuman River, Balbina Hydroelectric Power Station [2°02'S, 60°01'W], M. Costa, 19.II.1988; *Ephebopus cyanognathus* West & Marshall, 2000, *Ephebopus rufescens* West & Marshal, 2000 and *Ephebopus foliatus* West, Marshall, Fukushima and Bertani, 2008 data were taken from West et al. (2008); *Heteroscodra maculata* Pocock, 1899, male, Africa, pet trade (IBSP 9642), female, Guinea-Bissau, pet trade (IBSP 9644); *Psalmopoeus cambridgei* Pocock, 1895, male, Trinidad–Tobago, pet trade (IBSP 9653); *Psalmopoeus* sp., female, Venezuela, pet trade (IBSP 9655); *Stromatopelma* sp., male, Sierra Leone, pet trade (IBSP 9665), female, Africa, pet trade (IBSP 11136); *Tapinauchenius violaceus* (Mello-Leitão, 1930), male paratype of *Typhochlaena purpureus* Schmidt, 1995, French Guiana (SMF 38046); female holotype of *Typhochlaena purpureus* Schmidt, 1995, French Guiana (SMF 38042). Eumenophorinae: *Pelinobius muticus* Karsch, 1885, male, Kenya (IBSP 8530), female born in captivity, pet trade (IBSP 9643). Harpactirinae: *Pterinochilus* sp., male, Angola, Buila-Dala [11°10'S, 20°12'E] (IBSP 9647), female, Africa, pet trade (IBSP 8765). Ischnocolinae: *Holothele rondoni* (Lucas & Bücherl, 1972), male holotype and female paratype, Brazil, state of Amazonas, Iauaretê [°36'N, 69°11'W] (IBSP 4090). Ornithoctoninae: *Haplopelma longipes* von Wirth & Striffler, 2005, male, Cambodia, Skuon [1°33'N, 104°55'E], A. Anderson, 10 July 2003 (MZSP 28761); *Haplopelma minax* (Thorell, 1897), female, Thailand, 1 mi E. Bangkok [13°43'N, 100°31'E] (IBSP 9645). Poecilotherinae: *Poecilotheria* sp., male, India, pet trade (IBSP 9660); *Poecilotheria ornata* Pocock, 1899, female, Sri Lanka (IBSP 8767). Selenocosmiinae: *Phlogiellus* sp., male, Vietnam, R. Blauman, April 1989 (MZSP 28762); *Phlogiellus* sp., female, data were taken from West et al. (2008). Theraphosinae: *Euathlus vulpinus* (Karsch, 1880), male, Chile, (IBSP 3817–A); female, Chile, Osorno [40°34'S, 73°09'W] (IBSP 3817–B); *Lasiodora* sp., male, Brazil, state of Paraíba, João Pessoa [7°07'S, 34°52'W] (IBSP 11143), female, Brazil, state of Pernambuco, Jaboatão dos Guararapes [8°06'S, 35°00'W], Conjunto Murebeca (IBSP 10293). Incertae sedis: *Encyocratella olivacea* Strand, 1907 (data collected from Gallon 2003, 2005).

**Figures 1–7.**
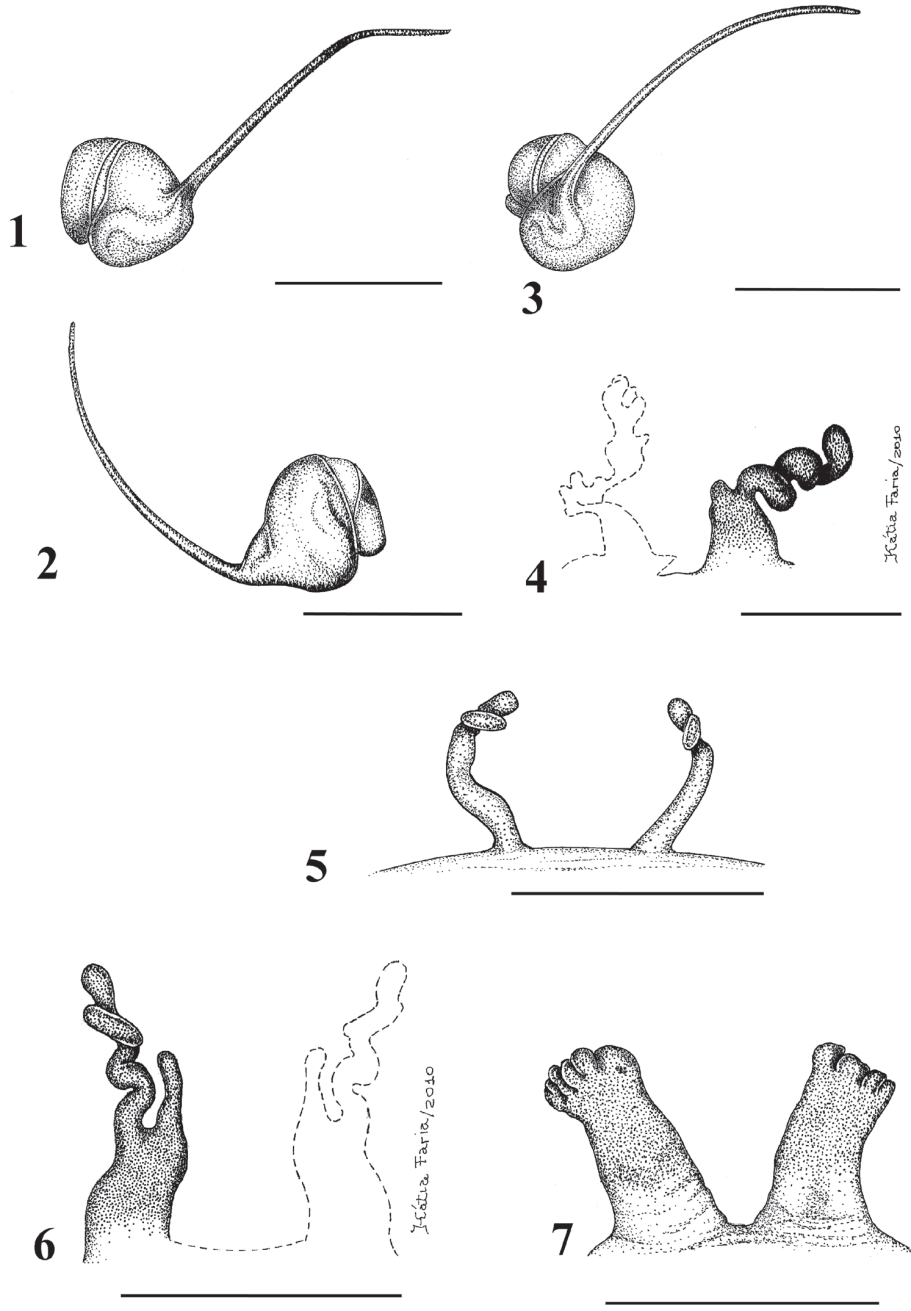
**1–5** *Typhochlaena seladonia* C. L. Koch, 1841 **1–3** male (IBSP 4551) left palpal bulb **1 **prolateral **2** retrolateral **3** frontal **4–5** females, spermathecae **4** exuvium (IBSP 4551) **5** female (IBSP 109718) **6** *Typhochlaena curumin* sp. n. holotype female (IBSP 8701) spermathecae **7** *Typhochlaena paschoali* sp. n.**,** paratype female (MNRJ 12928), spermathecae. Scale bar = 1mm.

*List of Characters*: (0) - Eye tubercle in males and females: (0) low (Fig. 36), (1) high (Fig. 91). (1) Anterior row of eyes in males and females, measured from the anterior margins: (0) procurved (Fig. 92), (1) straight (Fig. 37). (2) Clypeus in males and females: (0) not evident (Fig. 37), (1) narrow (Fig. 92), (2) wide. (3) Fovea in males and females, curvature: (0) straight, (1) recurved, (2) procurved. (4) Fovea in males and females, closure: (0) slit-like (closed), (1) pit-like (open). (5) Fovea in males and females, depth: (0) shallow, (1) deep. (6) Labial cuspules in males and females, number: (0) 30–300, (1) 0–20, (2) 350–450. (7) Sternum, shape: (0) longer than wide, not truncated behind (Fig. 93), (1) as long as wide, truncated behind (Fig. 20). Sternum is typically longer than wide in adult theraphosids, with few exceptions. However, in *Typhochlaena*, it is always as long as wide, or wider than long and truncated behind. Since this condition is present in immatures of other theraphosid species, this state seems to be neotenic in *Typhochlaena*, in agreement with the small size of adult individuals in all species of this genus. (8) Sigilla, posterior pair in males and females, position: (0) marginal, less than 1.5 times its own diameter from margin (Fig. 20), (1) located closer to the center of the sternum separated from marginby twice its own diameter. (9) Abdomen in females, (0) ovoid, about a half longer than wide (Figs 89–90), (1) dorso-ventrally flattened, about a quarter longer than wide (Figs 34–35). (10) Posterior lateral spinnerets, distal segment, (0) digitiform (Fig. 39), (1) domed (Fig. 21). Raven (1985) discussed the distribution of derived characters states in Mygalomorphae and suggested the most parsimonious hypothesis for digitiform distal segment of PLS is plesiomorphic. He proposed that this derived condition arose independently in some microstigmatids, nemesiids, ctenizoids and barychelids; and, in Theraphosidae, the plesiomorphic state is digitiform (Raven 1985: 13, 26). In the studied species, the derived condition was found in all *Typhochlaena* species. (11) Tarsus IV in males, cracked: (0) present, (1) absent. (12) Leg IV/I length ratio in females, (0) leg IV more than 10% longer than I, (1) leg IV roughly the same length as leg I, (2) leg IV more than 10% shorter than leg I. See discussion for character 13. (13) Leg IV/I length ratio in mature males, (0) leg IV more than 10% longer than I, (1) leg IV roughly the same length of leg I, (2) leg IV more than 10% shorter than leg I. Pocock (1901) distinguished his new genus *Iridopelma* from *Avicularia*, among other characters, by the longer leg I in relation to leg IV in males and females. In fact, male *Iridopelma hirsutum*, as well as male *Iridopelma oliveirai* sp. n. has a longer leg I in comparison to leg IV, but in other *Iridopelma* species (*Iridopelma zorodes*, *Iridopelma vanini* sp. n., and *Iridopelma katiae* sp. n.) male leg I and IV are approximately the same length (less than 10%). (14) Tarsal scopulae in males and females: (0) no true scopula; (1) scopula of sparse hairs; (2) dense scopula that does not extend much laterally; (3) scopula very extensive laterally, giving the tarsi and metatarsi I and IIa spatulate appearance. (15) Division of scopulae on metatarsus IV in males and females: (0) present; (1) absent. (16) Leg spines in males and females: (0) present on apex and other faces of tibiae and metatarsi, (1) present only on ventral apices of tibiae and metatarsi, (2) legs aspinose. (17) Palpal femora in males and females, scopula on retrolateral face: (0) absent, (1) present. (18) Femora IV in males and females, scopulae on retrolateral face: (0) absent, (1) present. (19) Chelicerae in males and females, scopulae on retrolateral face: (0) absent, (1) present. (20) Maxillae, spiniform setae on lower prolateral face: (0) absent, (1) present; (21) Stridulatory bristles in males and females form maxillae lyra: (0) absent, (1) present. (22) Stridulatory bristles on coxae I of males and females: (0) absent, (1) present. (23) Longitudinal white stripes on patellae and tibiae of males and females: (0) absent, (1) present. (24) Leg rings on distal femora, tibiae and metatarsi of males and females: (0) same color of the rest of segment, (1) white, (2) yellow. (25) Chelicerae of females, hair: (0) not iridescent, (1) iridescent. (26) Black marking dorsally on tibiae, metatarsi and tarsi of males and females: (0) absent, (1) present. (27) Female abdominal pattern: (0) all one color or irregularly mottled, (1) with pattern. (28) Color pattern ontogeny: (0) absent, (1) present. Only species in which individuals suffer a dramatic change in color pattern, and little of the juvenile pattern remains in adults, were considered. (29) Dorsal abdominal pattern in immatures: (0) homogeneous, (1) herringbone (Fig. 170), (2) with a zigzag central longitudinal dark stripe over a clear spot, which is marginated in black and connects to five narrow transversal black stripes (Fig. 171), (3) two median dorso-lateral spots (Fig. 172), (4) central longitudinal black stripe with 5–6 lateral stripes, connecting or not with the central stripe (Fig. 173), (5) black with a large central clear area having a longitudinal dark stripe, area closer to longitudinal dark stripe is usually reddish (Fig. 174), (6) two longitudinal dark stripes with five lateral stripes connecting to them (Fig. 175). (30) First instars color pattern: (0) brownish or grayish (Fig. 140), (1) metallic green (Fig. 40). First instars of some aviculariine species are almost completely metallic green (state 1), whereas, in other species they have a grayish to brownish general color pattern, normally with a black tarsi (state 0). (31) Spermathecae, number: (0) two, completely separated, (1), two, fused at base, (2) one, totally fused. (32) Spermathecal lobes: (0) absent, (1) present. (33) Spermathecae, shape: (0) straight or almost so (Figs 5, 7), (1) with a curvature on its middle (Fig. 11). Some aviculariine species have spermathecae long and curved in the middle, giving them an “M” shape (Fig. 11). (34) Spermathecae: (0) non-spiraled, (1) spiraled. (35) Spiraled spermathecae: (0) with a single fold (Fig. 5), (1) with two folds (Figs 150–152), (2) with multiple folds (Fig. 6). (36) Cymbium: (0) without spiniform process between lobes, (1) with spiniform process between lobes (fig. 84). (37) Prolateral cymbium lobe shape: (0) rounded, (1) subtriangular. (38) Subtegulum: (0) small, (1) large, extending down the bulb for half of the tegulum. (39) Tegulum: (0) without depression (Fig. 83), (1) with slight depression (Fig. 3), (2) with accentuated depression (Fig. 176). Retrolateral tegulum lacks a depression in the junction with the subtegulum in some taxa (state 0), whereas, other mygalomorphs can have a slight (state 1), to an accentuated (state 2), depression. From the studied specimens, state 2 was found only in *Avicularia* spp.1 (Fig. 176). (40) Embolus curvature to retrolateral side: (0) straight or very slightly curved (0 to 40°) (Fig. 31), (1) curved (50 to 120°) (Fig. 83); (2) strongly curved (more than 130°). (41) Embolus length: (0) 1.5–2.5 times longer than tegulum (Fig. 81), (1) shorter than tegulum, (2) more than 3 times longer than tegulum (Fig. 1). (42) Embolus distal width: (0) thin, less than 1/5 of tegulum height, (1) thick, more than 1/3 of tegulum height. (43) Embolus shape: (0) not flattened, (1) slightly flattened, (2) very flattened. (44) Prolateral inferior keel of bulb: (0) absent, (1) present. (45) Prolateral superior keel on embolus: (0) absent, (1) present. (46) Apical keel on embolus: (0) absent, (1) present. (47) Retrolateral keel on embolus: (0) absent, (1) present. (48) Male tibial spurs: (0) present, two-branched, (1) present, with an apical megaspine, (2) present, with spiniform setae, (3) not evident. (49) Male tibial spurs: (0) absent on leg II, (1) present on leg II (Fig. 86), which is a classical character used by Pocock (1901) as one of the characters defining *Iridopelma*. Presence of a tibial spur on leg II is known in theraphosids exclusively in *Iridopelma* species. (50) Hairs on metatarsi and tibiae I–IV of males: (0) normal, (1) long hairs laterally projected, forming a brush. (51) Urticating hairs of males and females: (0) only non-urticating hairs present on the abdomen and pedipalps, (1) urticating hairs on dorsum of abdomen, (2) urticating hairs on distal prolateral face of pedipalps. (52) Type I urticating hairs in males and females: (0) absent, (1) present. (53) Type II urticating hairs in males and females: (0) absent, (1) present. (54) Type III urticating hairs in males and females: (0) absent, (1) present. (55) Type IV urticating hairs in males and females: (0) absent, (1) present. (56) Type V urticating hairs in males and females: (0) absent, (1) present. (57) Burrow entrance (fossorial) of female: (0) simple with little or no silk, (1) collar of silk bound with surrounding debris, (2) trumpet-shaped. (58) Eggsac type: (0) mobile, (1) fixed hammock, (2) fixed flat. (59) Habitat: (0) evergreen forest, (1) deciduous forest, (2) desert-scrub, (3) “campo rupestre” - characterized by their height above sea level (above 900m), in association with a high degree of outcropping and consequent reduction of soil depth (Giulietti and Pirani 1988) (Figs 145–146). (60) Habits of female: (0) retreat within surface layers of soil, (1) arboreal, (2) opportunistic, (3) fossorial. (61) Retreats, made by adult arboreal: (0) built on tree trunk or on palm tree leaf base, (1) built in leaves, normally with two or more leaves connected by silk (Figs 101–105), (2) built under loosened tree bark (Fig. 23), (3) bromelicolous (Figs 75–80, 147–149). All arboreal species make a retreat with silk, but they differ in the preferred substrate. In Trinidad, *Avicularia* juveniles use leaves of low growing plants, but adults prefer trunks of large trees (Stradling 1994). In the Brazilian Amazon, it is common to find *Avicularia* retreats on the base of palm tree leaves (pers. obs.). *Poecilotheria*, *Stromatopelma* and *Heteroscodra* adults also make their retreats on tree trunks or in palm trees. In some *Iridopelma* species, *Avicularia diversipes*, *Avicularia sooretama* and *Avicularia gamba*, the retreat is made on leaves, normally connecting two or more leaves with silk. *Typhochlaena seladonia* and *Typhochlaena curumim* sp. n. were recorded with retreats made under loosened tree bark. *Pachistopelma* spp. are found exclusively inside bromeliads and *Iridopelma katiae* sp. n. seems to prefer bromeliads.

*Computer methods*: A data matrix (Table 1) with 62 characters and 38 taxa was analyzed with NONA 2.0 for Windows (Goloboff 1998) and X-PEE-WEE 1.3 for Windows (Goloboff 1997). The commands h1000, h/20, amb- and mult*50 were used. Concavities 1 to 6 were used with PEE-WEE. All characters were treated as non-additive. This is discussed fully below in cladistics.

**Table 1.** Data matrix showing distribution of character states in cladistic analysis. (?=unknown, - = non-applicable, both treated as missing data).<br/>

	**0**	**1**	**2**	**3**	**4**	**5**	**6**	**7**	**8**	**9**	**10**	**11**	**12**	**13**	**14**	**15**	**16**	**17**	**18**	**19**	**20**	**21**	**22**	**23**	**24**	**25**	**26**	**27**	**28**	**29**	**30**	**31**	**32**	**33**	**34**	**35**	**36**	**37**	**38**	**39**	**40**	**41**	**42**	**43**	**44**	**45**	**46**	**47**	**48**	**49**	**50**	**51**	**52**	**53**	**54**	**55**	**56**	**57**	**58**	**59**	**60**	**61**
*Melloina*	0	0	0	0	0	0	0	0	0	0	0	0	0	0	0	0	0	0	0	0	0	0	0	0	0	0	0	0	0	0	0	0	0	0	0	-	0	0	0	0	0	0	0	0	0	0	0	0	0	0	0	0	0	0	0	0	0	-	?	0	0	-
*Holothele*	0	0	1	1	0	0	0	0	0	0	0	0	0	0	1	0	0	0	0	0	0	0	0	0	0	0	0	0	0	0	0	0	1	0	0	-	0	0	0	1	1	0	0	0	0	0	0	0	0	0	0	0	0	0	0	0	0	0	0	0	3	-
*Stromatopelma*	0	1	1	-	1	0	0	0	0	0	0	1	1	1	3	0	2	0	0	0	0	0	0	0	1	0	1	1	0	1	0	0	0	0	0	-	0	1	0	0	0	0	0	0	0	0	0	0	3	0	1	0	0	0	0	0	0	-	2	0	1	0
*Heteroscodra*	0	1	1	-	1	0	0	0	0	0	0	1	0	1	3	0	2	0	0	0	0	0	0	0	1	0	1	1	0	1	0	0	0	0	0	-	0	1	0	0	0	0	0	0	0	0	0	0	3	0	1	0	0	0	0	0	0	-	2	0	1	0
*Pterinochilus*	1	0	2	0	0	0	0	0	0	0	0	1	1	2	2	0	0	0	0	1	0	0	0	0	1	0	0	1	0	1	0	0	0	0	0	-	0	1	0	0	0	0	0	0	0	0	0	0	1	0	0	0	0	0	0	0	0	0	1	2	3	-
*Pelinobius*	1	0	2	2	0	1	0	0	1	0	0	1	0	0	2	1	1	1	0	0	0	0	1	0	1	0	0	0	0	0	0	0	1	0	0	-	0	1	0	1	0	0	0	0	0	0	0	0	3	0	0	0	0	0	0	0	0	0	1	2	3	-
*Encyocratella*	1	0	1	-	1	1	1	0	0	0	0	1	1	1	2	0	0	1	0	0	0	0	0	0	1	0	0	1	0	1	0	-	-	-	-	-	0	1	0	?	0	0	1	2	1	1	0	0	3	0	0	0	0	0	0	0	0	-	1	?	1	0
*Haplopelma*	1	0	2	0	0	0	0	0	1	0	0	1	1	1	2	0	0	0	0	1	1	0	0	0	1	0	0	1	0	1	0	2	-	0	-	-	0	1	1	0	1	0	1	2	1	1	1	1	2	0	0	0	0	0	0	0	0	2	0	0	3	-
*Phlogiellus*	0	1	0	2	0	1	2	0	1	0	0	0	1	1	2	0	1	0	0	0	0	1	0	0	1	0	0	0	0	0	0	0	0	0	0	-	0	1	0	1	0	0	0	0	0	0	0	0	3	0	0	0	0	0	0	0	0	0	0	0	3	-
*Lasiodora*	1	0	2	0	0	1	0	0	0	0	0	1	1	1	2	0	0	0	1	0	0	0	1	0	1	0	0	0	0	0	0	1	0	0	-	-	0	0	1	0	1	1	1	1	1	1	1	1	0	0	0	1	1	0	1	0	0	0	0	0	3	-
*Euathlus*	1	0	1	0	0	1	1	0	0	0	0	1	?	2	2	0	0	0	0	0	0	0	0	0	1	0	0	0	0	3	0	0	1	0	-	-	0	0	1	0	1	0	0	0	1	1	0	0	0	0	0	1	0	0	1	1	0	0	0	1	3	-
*Poecilotheria*	0	0	1	0	0	0	0	0	0	0	0	1	1	1	3	0	2	0	0	0	1	1	0	0	1	0	0	1	0	2	0	2	-	0	-	-	0	1	0	0	1	1	1	0	1	1	1	0	3	0	0	0	0	0	0	0	0	-	0	1	1	0
*Psalmopoeus*	0	1	0	0	0	1	0	0	0	0	0	1	1	2	3	0	1	0	0	0	0	1	0	0	1	0	0	0	1	4	0	0	0	0	0	-	0	1	0	1	1	2	0	0	0	0	0	0	0	0	1	0	0	0	0	0	0	-	0	0	2	-
*Tapinauchenius*	0	1	0	0	0	1	0	0	0	0	0	1	1	1	3	0	1	0	0	0	0	0	0	0	1	0	0	0	1	4	0	0	0	0	0	-	0	1	0	0	0	2	0	0	0	0	0	0	0	0	1	0	0	0	0	0	0	-	0	0	2	-
*Ephebopus murinus*	1	1	0	0	0	1	0	0	0	0	0	1	2	2	3	0	1	0	0	0	0	0	0	1	1	0	0	0	1	0	0	0	0	0	0	-	0	1	0	1	0	0	0	0	0	0	0	0	0	0	0	2	0	0	0	0	1	2	0	0	3	-
*Ephebopus uatuman*	1	1	0	0	0	1	0	0	0	0	0	1	1	1	3	0	1	0	0	0	0	0	0	0	2	0	0	0	1	0	0	0	0	0	0	-	0	1	0	1	0	2	0	0	0	0	0	0	0	0	0	2	0	0	0	0	1	1	0	0	3	-
*Ephebopus cyanognathus*	1	1	0	0	0	1	0	0	0	0	0	1	1	2	3	0	1	0	0	0	0	0	0	0	2	1	0	0	1	0	0	0	0	0	0	-	0	1	0	?	0	2	0	0	0	0	0	0	0	0	0	2	0	0	0	0	1	1	0	0	3	-
*Ephebopus foliatus*	1	1	0	0	0	1	0	0	0	0	0	1	1	1	3	0	1	0	0	0	0	0	0	0	2	0	0	1	?	?	?	0	0	0	0	-	0	1	0	?	0	2	0	0	0	0	0	0	0	0	0	2	0	0	0	0	1	?	?	0	?	-
*Ephebopus rufescens*	1	1	0	0	0	1	0	0	0	0	0	1	1	1	3	0	1	0	0	0	0	0	0	0	1	0	0	0	1	0	0	0	0	0	0	-	0	1	0	?	0	0	0	0	0	0	0	0	0	0	0	2	0	0	0	0	1	1	0	0	2	-
*Avicularia juruensis*	1	0	1	0	0	1	0	0	0	0	0	1	0	0	3	0	2	0	0	0	0	0	0	0	2	0	0	0	1	4	0	0	0	1	0	-	1	1	0	2	1	2	0	0	0	0	0	0	2	0	0	1	0	1	0	0	0	-	0	0	1	0
*Avicutaria* sp.	1	0	1	0	0	1	0	0	0	0	0	1	0	0	3	0	2	0	0	0	0	0	0	0	1	0	0	0	1	4	0	0	0	1	0	-	1	1	0	2	1	2	0	0	0	0	0	0	2	0	0	1	0	1	0	0	0	-	0	0	1	0
*Avicularia diversipes*	1	0	1	0	0	0	0	0	0	0	0	1	1	1	3	0	2	0	0	0	0	0	0	0	2	0	0	1	1	6	1	0	0	1	0	-	1	1	0	0	2	2	0	0	0	0	0	0	3	0	0	1	0	1	0	0	0	-	0	0	1	1
*Avicularia sooretama*	1	0	1	0	0	0	0	0	?	0	0	1	1	1	3	0	2	0	0	0	0	0	0	0	1	0	0	1	1	6	1	0	1	0	0	-	0	1	0	1	1	2	0	0	0	0	0	0	3	0	0	1	0	1	0	0	0	-	?	0	1	1
*Avicularia gamba*	1	0	1	0	0	0	0	0	?	0	0	1	1	1	3	0	2	0	0	0	0	0	0	0	1	0	0	1	1	6	1	0	0	0	0	-	0	1	0	0	1	2	0	0	0	0	0	0	3	0	0	1	0	1	0	0	0	-	?	0	1	1
*Avicularia taunayi*	1	0	1	0	0	1	0	0	0	0	0	1	1	1	3	0	2	0	0	0	0	0	0	0	1	0	0	1	1	4	0	0	1	1	0	-	1	1	0	2	1	2	0	0	0	0	0	0	2	0	0	1	0	1	0	0	0	-	0	1	1	0
*Pachistopelma rufonigrum*	0	1	0	0	0	0	0	0	0	1	0	1	0	0	3	0	2	0	0	0	0	0	0	0	0	0	0	0	1	4	1	0	0	0	0	-	1	1	0	0	0	2	0	0	0	0	0	0	2	0	0	1	0	1	0	0	0	-	0	-	1	3
*Pachistopelma bromelicola* sp. n.	0	1	0	0	0	0	0	0	0	1	0	1	0	0	3	0	2	0	0	0	0	0	0	0	0	0	0	0	1	4	1	0	0	0	0	-	1	1	0	0	0	2	0	0	0	0	0	0	2	0	0	1	0	1	0	0	0	-	0	-	1	3
*Typhochlaena seladonia*	0	0	1	0	0	0	0	1	0	0	1	1	1	1	3	0	1	0	0	0	0	0	0	0	0	1	0	1	0	4	0	0	0	0	1	2	0	1	0	1	1	2	0	0	0	0	0	0	3	0	0	1	0	1	0	0	0	-	?	0	1	2
*Typhochlaena amma* sp. n.	1	0	1	0	0	0	0	1	0	0	1	1	0	1	3	0	2	0	0	0	0	0	0	0	1	1	0	1	?	5	0	0	0	1	0	-	0	1	0	0	1	0	0	0	0	0	0	0	3	0	1	1	0	1	0	0	0	-	?	0	1	?
*Typhochlaena curumim* sp. n.	1	0	1	0	0	0	0	1	0	0	1	?	0	?	3	0	2	0	0	0	0	0	0	0	1	0	0	1	0	4	0	0	1	0	1	2	?	?	?	?	?	?	?	?	?	?	?	?	?	?	?	1	0	1	0	0	0	-	?	0	1	2
*Typhochlaena paschoali* sp. n.	1	0	1	0	0	0	0	1	0	0	1	?	1	?	3	0	1	0	0	0	0	0	0	0	1	0	0	1	0	5	0	0	1	0	0	-	?	?	?	?	?	?	?	?	?	?	?	?	?	?	?	1	0	1	0	0	0	-	?	0	?	?
*Typhochlaena costae* sp. n.	1	0	1	0	0	0	0	1	0	0	1	1	1	1	3	0	2	0	0	0	0	0	0	0	1	0	0	1	0	4	0	0	0	0	0	-	0	1	0	0	1	0	0	0	0	0	0	0	3	0	0	1	0	1	0	0	0	-	?	1	?	?
*Iridopelma hirsutum*	1	0	1	0	0	0	0	0	0	0	0	1	1	2	3	0	2	0	0	0	0	0	0	0	1	0	0	1	1	5	1	0	1	0	1	0	1	1	0	0	1	0	0	0	0	0	0	0	2	1	0	1	0	1	0	0	0	-	?	0	1	1
*Iridopelma zorodes*	1	0	1	0	0	0	0	0	0	0	0	1	1	1	3	0	2	0	0	0	0	0	0	0	1	0	0	0	1	5	1	0	0	0	0	-	1	1	0	0	1	0	0	0	0	0	0	0	2	1	0	1	0	1	0	0	0	-	?	0	1	1
*Iridopelma vanini* sp. n.	1	0	1	0	0	1	0	0	0	0	0	1	1	1	3	0	2	0	0	0	0	0	0	0	1	0	0	1	1	5	?	0	1	0	1	1	1	1	0	0	1	0	0	0	0	0	0	0	2	1	0	1	0	1	0	0	0	-	?	1	1	?
*Iridopelma katiae* sp. n.	1	0	1	0	0	0	0	0	0	0	0	1	1	1	3	0	2	0	0	0	0	0	0	0	1	0	0	1	1	5	0	0	1	0	0	-	1	1	0	0	1	0	0	0	0	0	0	0	2	1	0	1	0	1	0	0	0	-	0	3	1	3
*Iridopelma marcoi* sp. n.	1	0	1	0	0	0	0	0	0	0	0	?	1	?	3	0	2	0	0	0	0	0	0	0	1	1	0	0	?	?	?	0	1	0	1	1	?	?	?	?	?	?	?	?	?	?	?	?	?	?	?	0	0	0	0	0	0	-	?	1	1	?
*Iridopelma oliveirai* sp. n.	1	0	1	0	0	0	0	0	0	0	0	1	1	2	3	0	2	0	0	0	0	0	0	0	1	0	0	0	1	5	?	0	1	0	1	1	1	1	0	0	1	0	0	0	0	0	0	0	2	1	0	1	0	1	0	0	0	-	?	1	1	?

## Taxonomy

### Typhochlaena

C. L. Koch, 1850 revalidated

http://species-id.net/wiki/Typhochlaena

Figs 1 [Fig F2] [Fig F3] [Fig F4] –28

Typhochlaena C. L. Koch, 1850:75.
Avicularia :Simon 1892:171 (in part: *Avicularia seladonia*).
Iridopelma : Smith 1993:15 (in part: *Iridopelma seladonium*).

#### Type species.

*Mygale seladonia* C. L. Koch, 1841 by subsequent designation (Mello-Leitão 1923:332). Almeida-Santos et al. (2008) stated that Mello-Leitão (1923) “erroneously reestablished *Typhochlaena* without removing the type species, *Typhochlaena caesia* C. L. Koch, 1842, from *Avicularia*”, but they did not indicate where their information on the type species of the genus came from. The genus *Typhochlaena* was erected by Koch (1850) and originally included two species: *Typhochlaena seladonia* and *Typhochlaena caesia*. Neither Koch (1850) nor subsequent authors indicated the type species of *Typhochlaena*, until Mello-Leitão (1923), who designated *Typhochlaena seladonia* C. L. Koch, 1841.

#### Diagnosis. 

Differs from all other aviculariine genera by the domed, short distal segment of PLS (Fig. 21) and adults with sternum as long as wide, truncated behind (Fig. 20). Additionally, they are very small aviculariines having urticating hair type II on the dorsum of the abdomen and males lack both tibial spurs and spiniform process on the cymbium.

#### Description.

**Figures 16–21. F3:**
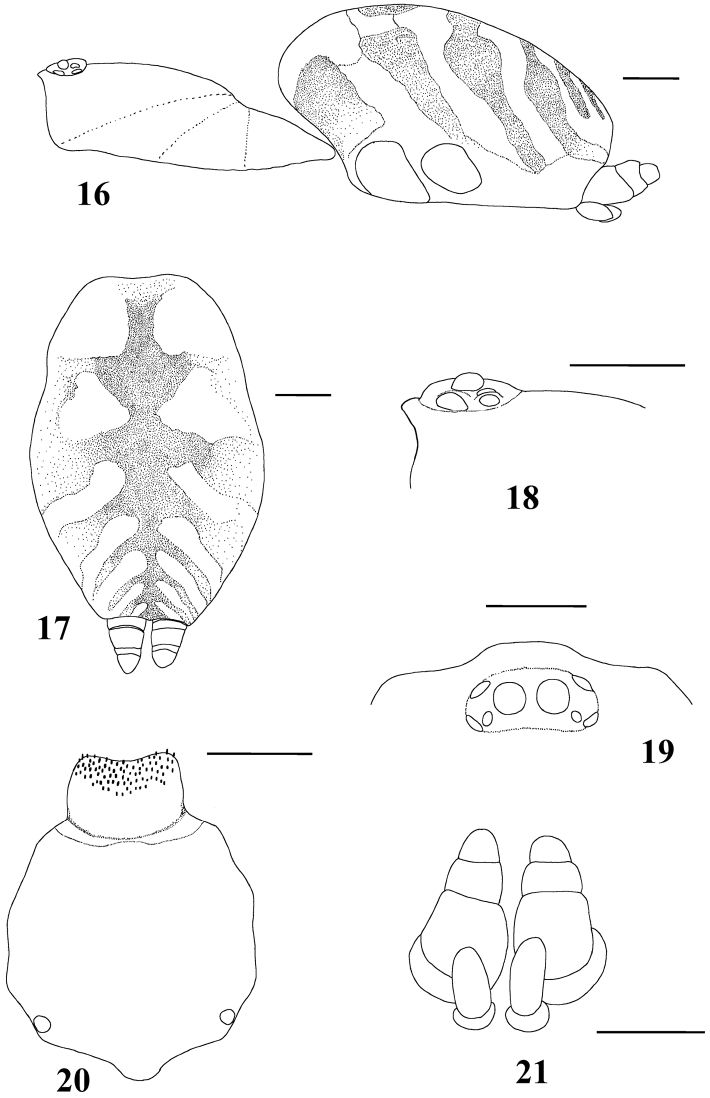
*Typhochlaena seladonia* C. L. Koch, 1841, female (IBSP 109718) **16** carapace and abdomen, lateral **17** abdomen, dorsal **18** eye tubercle, lateral **19** eye tubercle, dorsal **20** labium and sternum **21** spinnerets, ventral. Scale bar = 1mm.

Carapace as long as wide, or slightly longer than wide, cephalic region moderately raised (Fig. 16). Cephalic and thoracic striae shallow. Fovea shallow, straight. Chelicerae without rastellum. Eye tubercle low (*Typhochlaena seladonia*) (Fig. 18) or raised (other species), wider than long. Clypeus narrow (Figs 18–19). Anterior eye row procurved (Fig. 19). Labium wider than long, with ca. 58–122 cuspules concentrated on anterior half. Maxillary lyra absent. Maxilla subrectangular, anterior lobe distinctly produced into conical process, inner angle bearing (40–69) cuspules. Sternum wider than long or as long as wide, truncated behind (Fig. 20). Posterior angle not separating coxae IV. Three pairs of sigillae, all rounded, less than a quarter diameter from margin, sometimes not evident. Leg formula: IV I II III (except male *Typhochlaena amma* sp. n.: I IV II III). Clavate trichobothria on the distal 1/2 of tarsi I-IV. STC of males and femalewithout teeth. Tarsi I–III fully scopulated, IV divided by a band of setae. Scopulae of tarsi and metatarsi I–II extended very laterally giving them a spatulate appearance. Femur IV without retrolateral scopula. Legs lacking spines in males, female legs aspinose or with two ventro-apical spines on metatarsi III and/ or IV (*Typhochlaena seladonia*, *Typhochlaena paschoali* sp. n.). Posterior lateral spinnerets with distal article short, domed (Fig. 21). Stridulatory setae absent. Cymbium with two subequal lobes, the prolateral one triangular in shape (fig. 25 in West et al. 2008). Male spur on tibia I lacking. Male metatarsus I straight. Male palpal bulb globose narrowing abruptly forming a long slender embolus, 4 times (*Typhochlaena seladonia*) (Figs 1–3), 1.5 times (*Typhochlaena costae* sp. n.) (Figs 12–14), or 2.5 times (*Typhochlaena amma* sp. n.) (Figs 8–10) longer than tegulum length, with a curvature of roughly 60° (*Typhochlaena seladonia*, *Typhochlaena amma* sp. n.) (Figs 1–3, 8–10) or 45° (*Typhochlaena costae* sp. n.) (Figs 12–14) to retrolateral side, keels absent, tegulum with a slight prolateral depression (*Typhochlaena seladonia*) (Fig. 3), or absent (*Typhochlaena costae* sp. n., *Typhochlaena amma* sp. n.) (Figs 10, 14). Two spermathecae extremely variable in shape (Figs 4–7, 11, 15). Cymbium lacking spiniform process. Type II of urticating hair on abdomen dorsum of males and females. Ontogenetic change in color pattern lacking.

Males of *Typhochlaena curumim* sp. n. and *Typhochlaena paschoali* sp. n. are unknown.

**Figures 8–15. F2:** **8–11** *Typhochlaena amma* sp. n. **8–10** paratype male (MNRJ 12926), left palpal bulb **8** prolateral **9** retrolateral **10** frontal **11** holotype female (MNRJ 06239), spermathecae **12–15** *Typhochlaena costae* sp. n. **12–14** paratype male (IBSP unnumbered), left palpal bulb **12** prolateral **13** retrolateral **14** frontal **15** holotype female (IBSP unnumbered), spermathecae. Scale bar = 1mm.

Species included: *Typhochlaena seladonia* (Figs 1–5, 16–23), *Typhochlaena curumim* sp. n. (Figs 6, 26), *Typhochlaena paschoali* sp. n. (Figs 7, 27), *Typhochlaena amma* sp. n. (Figs 8–11, 24) and *Typhochlaena costae* sp. n. (Figs 12–15, 25).

#### Distribution and habitat.

Brazil: Northeastern, part of Central-West (state of Tocantins) and part of Southeastern (state of Espirito Santo) (Fig. 28). Specimens of *Typhochlaena* spp. are mostly found in Brazilian Atlantic rainforest, but one species seems to occur in drier, open environments (*Typhochlaena costae* sp. n.). Available data for two species (*Typhochlaena seladonia* and *Typhochlaena curumim* sp. n.) indicates they build a silky retreat under loose tree bark (Fig. 23).

**Figures 22–27. F4:**
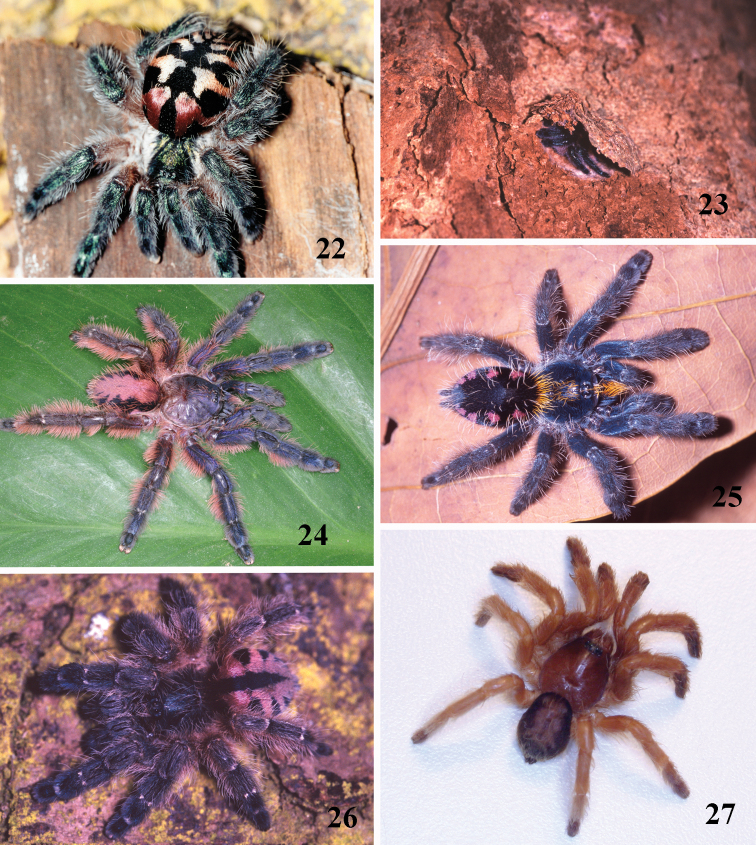
**22–23** *Typhochlaena seladonia* C. L. Koch, 1841, habitus **22** female, Santa Luzia do Itanhy, state of Sergipe **23** immature inside its retreat in tree bark, same locality **24** *Typhochlaena amma* sp. n., female, Santa Teresa, state of Espirito Santo **25** *Typhochlaena costae* sp. n., female, Palmas, state of Tocantins **26** *Typhochlena curumim* sp. n., female, Areia, state of Paraiba **27** *Typhochlaena paschoali* sp. n., preserved female, Camacam, state of Bahia (holotype MNRJ 13723). Photos: R. Bertani.

**Figure 28.**
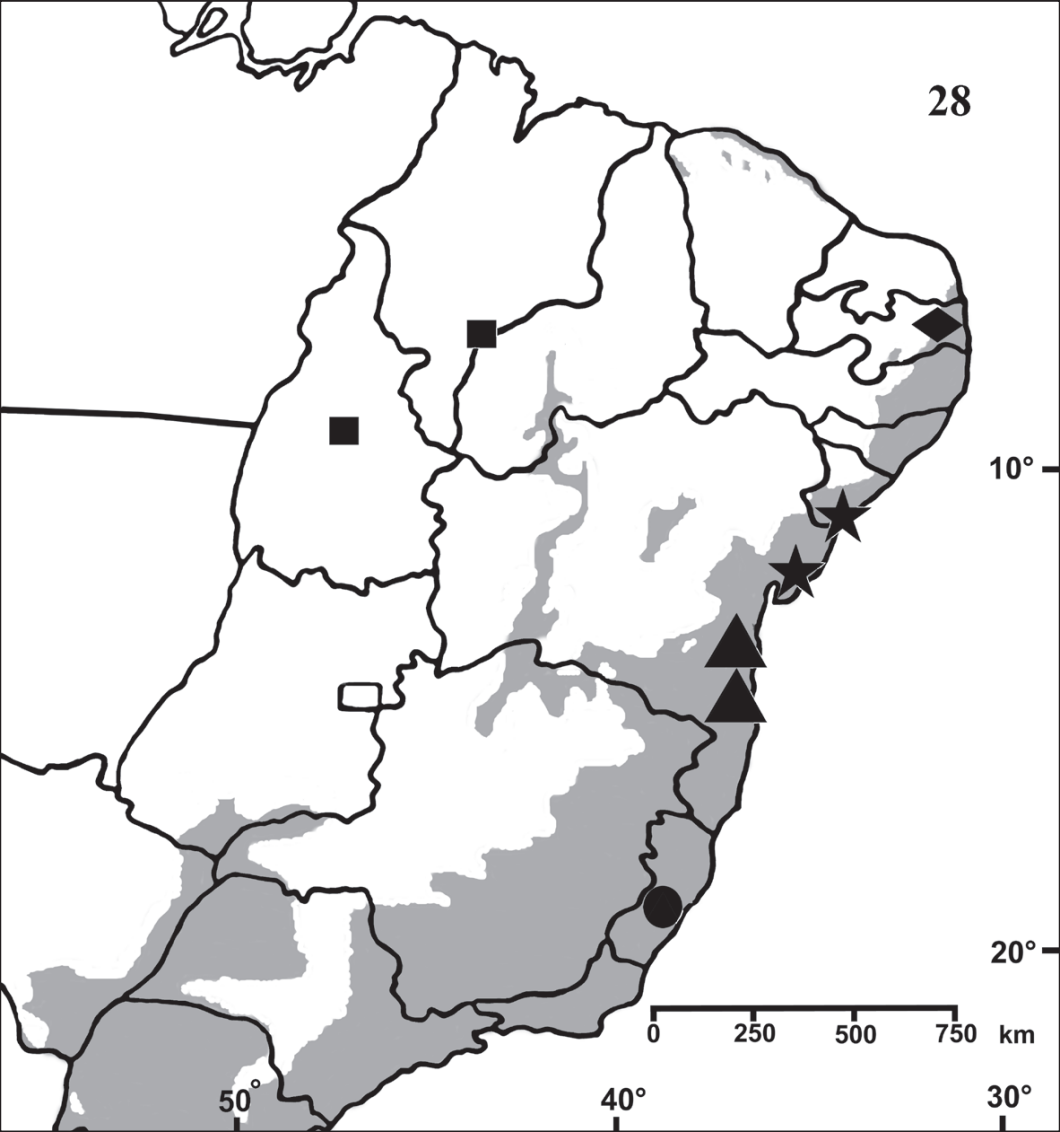
Map showing records of *Typhochlaena* species in Northestern, Central western and Southeastern Brazil. Star = *Typhochlaena seladonia* C. L. Koch, 1841, square = *Typhochlaena costae* sp. n., diamond = *Typhochlaena curumim* sp. n., triangle = *Typhochlaena paschoali* sp. n., circle = *Typhochlaena amma* sp. n. The gray area represents the approximate original distribution of Brazilian Atlantic rainforest. White area represents open environment (cerrado and caatinga).

#### Color pattern ontogeny.

Contrary to most aviculariines, ontogenetic changes are not known in species of *Typhochlaena*.

#### Key to species of *Typhochlaena*

(Males of *Typhochlaena curumim* sp. n. and *Typhochlaena paschoali* sp. n. are unknown)

**Table:** 

1	Male	2
–	Female	4
2	Embolus very long and slender (Figs 1–3); most of carapace and legs metallic green (Fig. 22); abdomen pattern as in Fig. 22	*Typhochlaena seladonia*
–	Embolus short (Figs 9, 13); coloration brownish or dark with blue sheen	3
3	Embolus too short (Figs 12–14), some long curled yellow setae over carapace and chelicerae (Fig. 25)	*Typhochlaena costae* sp. n.
–	Embolus longer (Figs 8–10), setae over carapace and chelicerae brownish	*Typhochlaena amma* sp. n.
4	Spermathecae strongly curved outwards from its base (Fig. 11); legs dark with blue sheen and with long reddish setae (Fig. 24)	*Typhochlaena amma* sp. n.
–	Spermathecae straight or almost so (Figs 5–7, 15); coloration otherwise	5
5	Spermathecae broad, straight, with multiple lobes at their tip (Fig. 7)	*Typhochlaena paschoali* sp. n.
–	Spermathecae slender or tapering from base to tip, without multiple lobes. (Figs 5–6, 15)	6
6	Spermathecae with lobes or folds (Figs 5–6); yellow curled setae over carapace and chelicerae lacking	7
–	Spermathecae lacking lobes and folds (Fig. 15); some long curled yellow setae over carapace and chelicerae (Fig. 25)	*Typhochlaena costae* sp. n.
7	Spermathecae slender throughout their length (Fig. 5); carapace and legs mostly metallic green (Fig. 22)	*Typhochlaena seladonia*
–	Spermathecae broad at base, tapering distally (Fig. 6); carapace and legs dark brown (Fig. 26)	*Typhochlaena curumim*sp. n.

### Typhochlaena seladonia

C. L. Koch, 1841 comb. restored

http://species-id.net/wiki/Typhochlaena_seladonia

Figs 1–5 16 –23 , 28

Mygale seladonia C. L. Koch 1841:39, Tab. CCC Fig. 716.
Typhochlaena seladonia : C. L. Koch 1850:75; Mello-Leitão 1923:335.
Avicularia seladonia : Simon 1892:171; Petrunkevitch 1911:50; Roewer 1942:255.
Iridopelma seladonia : Smith 1993:15 (transfer).
Iridopelma seladonium : Almeida-Santos et al. 2008:729; Platnick 2012.

#### Diagnosis.

Males differ from those of other species by the very long male palpal bulb embolus (Figs 1–3). The females differ by the long and slender spermathecae, spiraled distally (Figs 4–5). Additionally, males and females have cephalothorax metallic green and abdomen dorsum black with two series of six spots, the most anterior pair reddish, other yellowish (Fig. 22).

#### Types.

Holotype female of *Mygale seladonia* C. L. Koch, 1841 [dry, pinned] from Brazil, State of Bahia, Gomez, deposited at Museum für Naturkunde, Berlin (ZMB 2033), not examined, too brittle for mail.

#### Additional material examined.

Brazil: *Sergipe*: Santa Luzia do Itanhy, Mata do Crasto (11°23'S, 37°24'W) 1 immature male, A. D. Brescovit, 6 December 1996 (IBSP 115372); *Bahia*: Camaçari, Jacuipe [12°42'S, 38°07'W], 1 male, 1 female exuvium, T. Brazil ded., August 1980 (IBSP 4551); Salvador, Alphaville [12°56'S, 38°21'W], 1 female, G. G. Montingelli, 11 December 2001 (IBSP 109718).

#### Redescription.

Male (IBSP 4551). Carapace 4.6 long, 4.9 wide, chelicerae 2.4. Legs (femur, patella, tibia, metatarsus, tarsus, total): I: 5.6, 2.7, 4.0, 3.6, 2.0, 17.9. II: 5.5, 2.6, 3.7, 3.3, 1.7, 16.8. III: 4.7, 2.3, 3.4, 3.3, 1.7, 15.4. IV: 5.8, 2.4, 4.6, 4.6, 1.8, 19.2. Palp: 3.7, 2.0, 2.6, –, 0.9, 9.2. Mid-widths (lateral): femora I –IV = 1.1, 1.2, 1.2, 1.0, palp=0.8; patellae I–IV = 1.0, 1.1, 1.0, 1.0, palp = 1.0; tibiae I–IV = 0.9, 0.8, 0.8, 0.8, palp = 1.0; metatarsi I–IV = 0.7, 0.7, 0.6, 0.6; tarsi I–IV = 0.7, 0.7, 0.6, 0.5, palp = 0.9. Abdomen 5.2 long, 3.6 wide. Spinnerets: PMS, 0.6 long, 0.3 wide, 0.1 apart; PLS, 0.9 basal, 0.4 middle, 0.6 domed distal; mid-widths (lateral), 0.6, 0.4, 0.3, respectively. Carapace: length to width 0.94. Fovea 0.8 wide. Eyes: tubercle 0.1 high, length 0.6, width 1.3. Clypeus 0.3. Anterior eye row procurved, posterior slightly recurved. Eye sizes and inter-distances: AME 0.3, ALE 0.3, PME 0.1, PLE 0.2, AME–AME 0.2, AME–ALE 0.1, AME–PME 0.05, ALE–ALE 0.9, ALE–PME 0.2, PME–PME 0.7, PME–PLE 0.08, PLE–PLE 1.0, ALE–PLE 0.1, AME–PLE 0.2. Ratio of eye group width to length 2.54. Maxillae: length to width: 2.0. Cuspules: 46 spread over ventral inner heel. Labium: 0.6 long, 0.9 wide, with 58 cuspules spaced by one diameter from each other on the anterior half. Labio-sternal groove shallow, flat, sigilla not evident. Chelicerae: basal segments with seven teeth, the two more distal closely positioned, others spaced by at least one diameter from each other. Sternum: 2.1 long, 2.2 wide (Fig. 20). Legs: leg formula: IV I II III. Scopula: tarsi I–IV fully scopulate, IV divided by a four setae wide row. Metatarsi I–II fully scopulate; III 1/2 distal scopulate; IV 1/4 distal scopulate. IV divided by three wide row of setae. Spines absent on all legs and palps. Urticating hairs: type II (0.25 long, 0.01 wide) on the abdomen dorsum. Palp: embolus 2.4 in length, with a 60° curvature to the retrolateral side. Embolus basal, middle and distal width of 0.1, 0.07 and 0.03, respectively. Tegulum 0.6 long. (Figs 1–3). Cymbium: spiniform process lacking. Color pattern: carapace and chelicerae covered with metallic green setae, and pale yellow setae on both margins. Legs, palps, labium and sternum metallic green. Longitudinal stripes on femora, patellae, tibiae and metatarsi inconspicuous. Distal femora, patellae, tibiae and metatarsi rings very discrete, whitish. Abdomen dorsum black with two series of six spots. Most anterior pair reddish, other yellowish. Abdomen laterally and venter metallic green (Fig. 22).

#### Redescription.

Female (IBSP 109718) Carapace 5.4 long, 5.2 wide, chelicerae 2.5. Legs (femur, patella, tibia, metatarsus, tarsus, total): I: 4.2, 2.5, 2.5, 2.0, 1.7, 12.9. II: 3.7, 2.2, 2.4, 1.8, 1.7, 11.8. III: 3.5, 2.1, 2.1, 2.1, 1.4, 11.2. IV: 4.4, 2.1, 2.9, 2.7, 1.3, 13.4. Palp: 3.0, 1.9, 2.0, –, 2.3, 9.2. Mid-widths (lateral): femora I –IV = 1.1, 1.2, 1.2, 1.0, palp = 1.0; patellae I–IV = 1.3, 1.3, 1.2, 1.1, palp = 0.9; tibiae I–IV = 1.2, 1.2, 1.1, 1.0, palp = 1.0; metatarsi I–IV = 1.0, 0.9, 0.8, 0.8; tarsi I–IV = 1.0, 1.0, 0.8, 0.8, palp = 1.0. Abdomen 6.0 long, 4.0 wide. Spinnerets: PMS, 0.9 long, 0.3 wide, 0.1 apart; PLS, 0.7 basal, 0.3 middle, 0.3 domed distal; mid-widths (lateral), 0.6, 0.5, 0.4, respectively. Carapace: length to width 1.03. Fovea 0.8 wide. Eyes: tubercle 0.02 high, 0.7 long, 1.4 wide. Clypeus 0.3. Anterior eye row procurved, posterior slightly recurved. Eye sizes and inter-distances: AME 0.3, ALE 0.3, PME 0.1, PLE 0.2, AME–AME 0.1, AME–ALE 0.1, AME–PME 0.08, ALE–ALE 0.9, ALE–PME 0.2, PME–PME 0.8, PME–PLE 0.04, PLE–PLE 1.0, ALE–PLE 0.1, AME–PLE 0.2. Ratio of eye group width to length 2.72. Other characters as in male, except: maxillae: length to width: 1.6. Cuspules: *ca*. 50 spread over ventral inner heel. Labium: 0.8 long, 1.1 wide, with *ca*. 87 cuspules. Sternum: 2.3 long, 2.5 wide. Scopula: tarsi I–II fully scopulate, III with some sparse setae along its center, IV divided by a four setae row. Metatarsi I–II fully scopulate; III 1/2 distal scopulate; IV 1/5 distal scopulate. IV divided by three wide row of setae. Spines: metatarsi III and IV with two ventro-apical spines. Urticating hairs type II (0.43 to 0.48 long, 0.015 wide) on the abdomen dorsum. Genitalia: paired long, slender, curved, weakly sclerotized spermathecae spiraled distally.

#### Remarks.

Almeida-Silva et al. (2008) redescribed the female of *Iridopelma seladonium* (C. L. Koch, 1841) after examining photographs of the recently rediscovered holotype of *Mygale seladonia* C. L. Koch, 1841. They presented photographs of the holotype and an illustration displaying non-spiraled spermathecae from a fresh specimen from state of Bahia, Brazil. A female exuvium examined by myself in this work showed spermathecae with two folds (Fig. 4) and a dissected female showed a single fold spermathecae (Fig. 5). Spermathecae of exuvium could have been modified by the moulting process or by preservation conditions, but the dissected specimen is well preserved. The fold is not easily seen and possibly it was overlooked by Almeida-Silva et al. (2008). The unavailability of additional specimens prevented me from investigating this question more thoroughly.

#### Distribution.

Brazil, state of Sergipe and northeastern Bahia (Fig. 28).

#### Natural history.

One female was found under loose tree bark in Santa Luzia do Itanhy SE (September 1999). Fig. 23. Two females were found in a similar retreat in nature, in Bahia, and one specimen built a retreat with small pieces of tree bark in captivity (Almeida-Silva et al. 2008).

### Typhochlaena curumim sp. n.

urn:lsid:zoobank.org:act:818C7745-D8D8-4B32-B5F4-08059D64DDCA

http://species-id.net/wiki/Typhochlaena_curumim

Figs 6 , 26 , 28

#### Diagnosis.

The female differs by the spermatheca broad at its base, tapering to a single or bifid slender spiraled distal region (Fig. 6). Additionally, females have cephalothorax and legs dark brown or brown, and the abdomen metallic yellowish-green, dorsally with central longitudinal black stripe and a series of five black stripes on both sides, extending laterally (Fig. 26).Male unknown.

#### Etymology.

The specific nameis derived fromthe Brazilian indigenous Tupi language, meaning “child”. It refers to the local children that found the type specimens high in a tree in Areia, State of Paraíba, Brazil, during an arachnological expedition.

**Types.** Holotype female, Brazil, *Paraíba*, Areia, Reserva Ecológica Estadual Mata do Pau-Ferro [6°58'S, 35°42'W], 500 m a.s.l., under tree bark, A.D. Brescovit, R. Bertani, A.B.Bonaldo, S.C.Dias, September 1999 (IBSP 8701); Paratype female, same data (IBSP 8354).

#### Additional material examined.

None.

#### Description.

Holotype female (IBSP 8701). Carapace 5.0 long, 4.8 wide, chelicerae 2.5. Legs (femur, patella, tibia, metatarsus, tarsus, total): I: 3.2, 2.4, 2.3, 1.8, 1.3, 11.0. II: 3.1, 2.2, 2.2, 1.9, 1.3, 10.7. III: 2.7, 1.9, 2.1, 2.0, 1.4, 10.1. IV: 3.6, 2.2, 2.6, 2.6, 1.6, 12.6. Palp: 2.5, 1.7, 1.7, –, 2.0, 26.9. Mid-widths (lateral): femora I –IV = 1.2, 1.1, 1.1, 1.0, palp = 0.8; patellae I–IV = 1.0, 1.2, 1.0, 0.9, palp = 0.9; tibiae I–IV = 1.1, 1.1, 1.0, 0.9, palp = 1.0; metatarsi I–IV = 1.0, 1.0, 0.8, 0.7; tarsi I–IV = 1.1, 1.0, 0.8, 0.7, palp = 0.9. Abdomen 5.3 long, 3.3 wide. Spinnerets: PMS, 0.5 long, 0.3 wide, 0.1 apart; PLS, 0.4 basal, 0.3 middle, 0.3 domed distal; mid-widths (lateral), 0.4, 0.4, 0.3, respectively. Carapace: length to width 1.04. Fovea 1.4 wide. Eyes: tubercle 0.2 high, 1.0 long, 1.5 wide. Clypeus 0.2. Anterior eye row procurved, posterior straight. Eye sizes and inter-distances: AME 0.3, ALE 0.3, PME 0.2, PLE 0.2, AME–AME 0.2, AME–ALE 0.1, AME–PME 0.1, ALE–ALE 0.9, ALE–PME 0.3, PME–PME 0.8, PME–PLE 0.1, PLE–PLE 1.0, ALE–PLE 0.3, AME–PLE 0.3. Ratio of eye group width to length 2.1. Maxillae: length to width: 1.45. Cuspules: ca. 51 spread over ventral inner heel. Labium: 0.6 long, 1.0 wide, with 122 cuspules spaced by one diameter from each other on the anterior half. Labio-sternal groove shallow, flat, sigilla not evident. Chelicerae: basal segments with six larger teeth and three very small after the 3°, 5° and 7° teeth. Sternum: 1.9 long, 2.1 wide. Legs: leg formula: IV I II III. Scopula: tarsi I–III fully scopulate, IV divided by row of 6–7 setae. Metatarsi I–II 4/5 scopulate; III 1/2, IV 1/3 distal scopulate. IV divided by a six wide row of setae. Spines absent on all legs and palps. Urticating hairs type II (0.3 mm long, 0.01 wide) on the abdomen dorsally. Genitalia: paired spermathecae with two lobes distally, a long spiraled and a straight short (Fig. 6). Color pattern: carapace and chelicerae dark brown with pale yellow long hairs on the carapace border. Legs and palps dark brown, except for brown femora. Cephalic region, legs, palps and chelicerae covered with long and abundant chestnut setae. Coxae brown. Labium, sternum and maxilla dark brown. Longitudinal stripes on femora, patellae, tibiae and metatarsi inconspicuous. Distal femora, patellae, tibiae and metatarsi rings whitish. Abdomen metallic yellowish-green, dorsally with central longitudinal black stripe and a series of five black stripes on both sides, extending laterally. Several scattered white and very long guard hairs over abdomen dorsally (Fig. 26).

#### Distribution.

Known only from “Mata do Pau-Ferro”, Areia, in the state of Paraiba, Brazil (Fig. 28).

#### Natural history.

Three specimens were found high in a tree, under loose bark in “Mata do Pau-Ferro” reserve, September, 1999.

### Typhochlaena paschoali sp. n.

urn:lsid:zoobank.org:act:962A2C3A-127A-4656-9D3E-E9A1A9639CAD

http://species-id.net/wiki/Typhochlaena_paschoali

Figs 7 , 27 –28

#### Diagnosis.

The females differ from those of all other *Typochlaena* spp. by an almost straight, broad and short spermathecae, with a multilobular distal portion (Fig. 7). Male unknown. Additionally, females have cephalothorax and legs brown, abdomen black with dorsum with a central longitudinal white pattern having zigzag border (Fig. 27).

#### Etymology.

The specific name is a patronym in honour of Elbano Paschoal de Figueiredo Moraes, a Brazilian environmentalist who was died early on April 2011. He was one of the founders of the NGO “GAMBA – Grupo Ambientalista da Bahia”, and was well known for his efforts in preserving Brazilian Atlantic rainforest remnants in the state of Bahia, Brazil.

#### Types.

Holotype female and immature paratype, Brazil, state of Bahia, Camacan [15°24'S, 39°30'W], no further data (MNRJ 13723); Paratypes 1 female, 1 subadult male, 9 immatures, Brazil, state of Bahia, Jussari [15°10'S, 39°29'W], no further data (MNRJ 12928 – R2981).

#### Additional material examined.

Brazil, *Pernambuco*: Tapera [8°23'S, 38°05'W], 1 female, no further data (MNRJ 13615) (probably mislabeled); *Bahia*: Uruçuca, Faz. S. Teresa [14°35'S, 39°17'W], 2 females, 1 immature, no further data (MNRJ 12919 – R 2955); (CEPLAC), 1 subadult male, N. Tingarine (MNRJ 13761).

#### Description.

Holotype female (MNRJ 13723). Carapace 6.4 long, 6.0 wide, chelicerae 3.0. Legs (femur, patella, tibia, metatarsus, tarsus, total): I: 4.6, 3.3, 3.2, 2.4, 2.0, 15.5. II: 4.2, 2.8, 3.3, 2.4, 2.1, 14.8. III: 3.6, 2.6, 2.6, 2.5, 1.6, 12.9. IV: 4.6, 2.9, 3.7, 3.5, 1.8, 16.5. Palp: 3.5, 2.4, 2.1, –, 2.2, 10.2. Mid-widths (lateral): femora I –IV = 1.5, 1.5, 1.4, 1.3, palp = 0.9; patellae I–IV = 1.3, 1.4, 1.3, 1.3, palp = 1.2; tibiae I–IV = 1.3, 1.3, 1.2, 1.1, palp = 1.1; metatarsi I–IV = 1.3, 1.2, 0.9, 0.8; tarsi I–IV = 1.2, 1.1, 0.9, 0.7, palp = 1.2. Abdomen 6.4 long, 4.7 wide. Spinnerets: PMS, 0.5 long, 0.4 wide, 0.1 apart; PLS, 0.9 basal, 0.5 middle, 0.5 domed distal; mid-widths (lateral), 0.7, 0.6, 0.4, respectively. Carapace: length to width 1.0. Fovea 1.5 wide. Eyes: tubercle 0.1 high, 1.1 long, 1.8 wide. Clypeus 0.2. Anterior eye row procurved, posterior slightly recurved. Eye sizes and inter-distances: AME 0.3, ALE 0.4, PME 0.2, PLE 0.3, AME–AME 0.4, AME–ALE 0.2, AME–PME 0.2, ALE–ALE 1.3, ALE–PME 0.3, PME–PME 1.1, PME–PLE 0.1, PLE–PLE 1.5, ALE–PLE 0.3, AME–PLE 0.4. Ratio of eye group width to length 2.4. Maxillae: length to width: 1.7. Cuspules: 64 spread over ventral inner heel. Labium: 0.9 long, 1.3 wide, with 76 cuspules spaced by one diameter from each other on the anterior half. Labio-sternal groove shallow, flat, sigilla not evident. Chelicerae: basal segments with 8 teeth having similar size and well spaced from each other. Sternum: 3.1 long, 3.0 wide. Legs: leg formula: IV I II III. Scopula: tarsi I–III fully scopulate, IV divided by row of 6 setae. Metatarsi I–II 4/5 scopulate; III 2/3, IV 1/4 distal scopulate. IV divided by six wide row of setae. Spines: two apical spines on ventral metatarsus IV. Urticating hairs type II (0.32 to 0.4 long, 0.01 wide) on the abdomen dorsum. Genitalia: paired broad spermathecae very slightly curved outwards, ending in multilobular apex (Fig. 7). Color pattern (preserved in alcohol): carapace and chelicerae brown. Legs and palps light brown, except for tarsi and metatarsi I–III and tarsi of palp and leg IV, darker. Coxae, maxillae and labium light brown. Sternum dark brown. Longitudinal stripes on dorsum of femora, patellae, tibiae and metatarsi inconspicuous. Distal femora, patellae, tibiae and metatarsi with white rings. Abdomen black, dorsum with a central longitudinal white pattern having zigzag border. Cephalic area and chelicerae with long stiff setae. Abdomen dorsum with several scattered, very long, white guard-hairs (Fig. 27).

#### Distribution.

Known from southern state of Bahia, Brazil. A single record for Tapera, in the state of Pernambuco, Brazil is probably a label mistake (Fig. 28).

#### Natural history. 

No data available.

### Typhochlaena amma sp. n.

urn:lsid:zoobank.org:act:C177BD29-700C-4B94-AFA5-211FA5C37A1A

http://species-id.net/wiki/Typhochlaena_amma

Figs 8–11 , 24 , 28

#### Diagnosis.

The male differs from those of *Typhochlaena seladonia* by shorter and broader embolus (Figs 8–10). Differs from *Typhochlaena costae* sp. n. by the embolus longer and stouter. The females differ by the strongly curved spermathecae, diverging, in their basal portion (Fig. 11). Additionally, the females have cephalothorax and legs covered with dark setae having a blue metallic sheen under light, abdomen black ventrally and dorsally with a central pinkish stripe having zigzag border (Fig. 24). The males are light brown and the abdomen is black with a dorsally pale stripe similar in shape to that of the female.

#### Etymology.

The specific name refers to the project AMMA – arachnids and myriapods from Brazilian Atlantic rainforest carried out by the arachnologists from Museu Nacional, Rio de Janeiro, who collected the types.

#### Types.

Holotype female, Brazil, *Espírito Santo*, Santa Teresa, Estação Ecológica de Santa Lúcia [19°58'S, 40°32'W], 672 m.a.s.l, A. P. L. Giupponi, February 2008 (MNRJ 06239); Paratype female, same locality, A. Pérez Gonzalez, January 2005 (MNRJ 06240); Paratype male, same locality, no further data (MNRJ 12926); Paratypes 2 males, Domingos Martins, Pico do Eldorado [20°22'S, 40°39'W], M. T. Tavares *et al*., 13 December 2004 (IBSP 13005).

#### Additional material examined.

None.

#### Description.

Holotype female (MNRJ 06239). Carapace 10.3 long, 10.3 wide, chelicerae 5.1. Legs (femur, patella, tibia, metatarsus, tarsus, total): I: 7.3, 5.4, 5.7, 4.5, 2.8, 25.7. II: 7.1, 4.8, 5.1, 4.5, 2.7, 24.2. III: 6.3, 4.5, 5.0, 4.7, 3.0, 23.5. IV: 7.9, 4.6, 6.8, 6.4, 2.9, 28.6. Palp: 5.1, 3.5, 3.4, –, 3.5, 15.5. Mid-widths (lateral): femora I–IV=2.5, 2.6, 2.6, 2.4, palp = 2.0; patellae I–IV = 2.3, 2.3, 2.2, 2.2, palp = 1.9; tibiae I–IV = 2.3, 2.3, 2.1, 2.0, palp = 2.0; metatarsi I–IV = 1.9, 2.0, 1.6, 1.2; tarsi I–IV = 1.6, 1.8, 1.6, 1.4, palp = 1.9. Abdomen 12.0 long, 8.9 wide. Spinnerets: PMS, 1.6 long, 0.7 wide, 0.2 apart; PLS, 1.6 basal, 0.9 middle, 1.0 domed distal; mid-widths (lateral), 1.2, 1.0, 0.6, respectively. Carapace: length to width 1.0. Fovea 1.8 wide. Eyes: tubercle 0.1 high, 1.1 long, 1.8 wide. Clypeus 0.3. Anterior eye row procurved, posterior slightly recurved. Eye sizes and inter-distances: AME 0.5, ALE 0.5, PME 0.3, PLE 0.5, AME–AME 0.6, AME–ALE 0.3, AME–PME 0.2, ALE–ALE 1.9, ALE–PME 0.5, PME–PME 1.6, PME–PLE 0.1, PLE–PLE 2.1, ALE–PLE 0.5, AME–PLE 0.6. Ratio of eye group width to length 2.3. Maxillae: length to width: 1.8. Cuspules: 51 spread over ventral inner heel. Labium: 1.7 long, 2.0 wide, with 79 cuspules spaced by one diameter from each other on the anterior third. Labio-sternal groove shallow, flat, sigilla not evident. Chelicerae: basal segments with 7 teeth well spaced from each other. Sternum: 4.7 long, 4.3 wide. Leg formula: IV I II III. Scopula: tarsi I–III fully scopulate, IV divided by six wide row of setae. Metatarsi I–II 4/5 scopulate; III 1/2, IV 1/4 distal scopulate. IV divided by six wide row of setae. Spines absent on all legs and palps. Urticating hairs type II (0.42 to 0.56 long, 0.01 wide) on the abdomen dorsum. Genitalia: paired spermathecae curving abruptly outwards from their base (more than 90°) and with a partial division of their apex into two lobes (Fig. 11). Color pattern: carapace, chelicerae, sternum, maxillae and legs covered with dark setae having a blue metallic sheen under light. Legs, mainly ventrally, with long chestnut-brown setae. Longitudinal stripes on femora, patellae, tibiae and metatarsi inconspicuous. Distal femora, patellae, tibiae and metatarsi with white rings. Abdomen black ventrally and dorsally with a central pinkish stripe having zigzag border (Fig. 24).

#### Description.

Paratype male (MNRJ 12926). Carapace 7.3 long, 7.7 wide, chelicerae 3.2. Legs (femur, patella, tibia, metatarsus, tarsus, total): I: 8.9, 4.4, 7.7, 6.7, 3.0, 30.7. II: 7.8, 4.2, 6.9, 6.0, 2.6, 27.5. III: 6.0, 3.5, 5.1, 4.8, 2.4, 21.8. IV: 8.1, 4.0, 7.8, 7.2, 2.4, 29.5. Palp: 4.6, 2.9, 3.5, –, 1.6, 12.6. Mid-widths (lateral): femora I–IV = 1.1, 1.4, 1.8, 1.1, palp = 0.8; patellae I–IV = 1.5, 1.5, 1.4, 1.6, palp = 1.1; tibiae I–IV = 1.3, 1.1, 1.5, 1.4, palp = 1.3; metatarsi I–IV = 1.1, 1.0, 1.0, 0.9; tarsi I–IV = 1.0, 1.1, 1.0, 0.9, palp = 1.1. Abdomen 6.6 long, 3.8 wide. Spinnerets: PMS, 0.8 long, 0.4 wide, 0.1 apart; PLS, 1.1 basal, 0.6 middle, 0.6 domed distal; mid-widths (lateral), 0.6, 0.5, 0.3, respectively. Carapace: length to width 0.95. Fovea 1.4 wide. Eyes: tubercle 0.6 high, 1.4 long, 2.3 wide. Clypeus 0.1. Anterior eye row procurved, posterior slightly recurved. Eye sizes and inter-distances: AME 0.5, ALE 0.5, PME 0.2, PLE 0.3, AME–AME 0.3, AME–ALE 0.3, AME–PME 0.2, ALE–ALE 1.6, ALE–PME 0.5, PME–PME 1.4, PME–PLE 0.1, PLE–PLE 1.7, ALE–PLE 0.3, AME–PLE 0.5. Ratio of eye group width to length 2.3. Other characters as in female, except: maxillae: length to width: 1.9. Cuspules: 40 spread over ventral inner heel. Labium: 0.9 long, 1.5 wide, with 71 cuspules spaced by one diameter from each other on the anterior half. Chelicerae: basal segments with seven teeth spaced by at least one diameter from each other. Sternum: 3.4 long, 3.2 wide. Legs: leg formula: I IV II III. Scopula: tarsi I–IV fully scopulate, IV divided by six wide row of setae. Metatarsi I–II 4/5 scopulate; III 1/2 distal scopulate; IV 1/4 distal scopulate. IV divided by six wide row of setae. Urticating hairs type II (0.51 to 0.70 long, 0.01 wide) on the abdomen dorsum. Palp: embolus 2.2 long, with a 60° curvature retrolaterally. Embolus basal, middle and distal width of 0.3, 0.2 and 0.05, respectively. Tegulum 0.9 long. (Figs 8–10). Color pattern: (specimen is preserved and faded) carapace, chelicerae, palps, legs, sternum and labium are light brown. Abdomen black with a dorsally pale stripe similar in shape to the female.

#### Distribution.

Known only from Santa Teresa and Domingos Martins, in the mountain range of state of Espirito Santo, Brazil (Fig. 28).

### Typhochlaena costae sp. n.

urn:lsid:zoobank.org:act:1EAFD951-7AE2-46AA-9373-618A78B1A8FB

http://species-id.net/wiki/Typhochlaena_costae

Figs 12–15 , 25 , 28

#### Diagnosis.

Males differ from those of other species by the short and slender embolus (Figs 12–14). Females differ by the non-spiraled spermathecae, lacking lobes, diverging on their base and converging on their distal portions (Fig. 15). Additionally, males and females have cephalothorax brown and abdomen dorsally black with two series of four red spots extending laterally. Carapace, dorsum of chelicerae and most anterior dorsal region of abdomen with very long, yellow, stiff setae (Fig. 25).

#### Etymology.

The specific name is a matronym in honour of Miriam Costa, who collected the holotype and several other new spider species during several years she worked at Instituto Butantan.

#### Types.

Holotype female, Brazil, state of Tocantins, Palmas, U.H.E. Luis Eduardo Magalhães [10°12'S, 48°24'W], 211 m.a.s.l, during faunal rescuing, M. Costa and D. Candido, 12 January 2002 (IBSP unnumbered); Paratype male, Brazil, state of Tocantins, Lajeado, (9°46'4.85"S, 48°21'6.69"W), 226 m.a.s.l, G. Puorto, R. Martins, I. Knysak (pitfall trap), April 2002 (IBSP unnumbered).

#### Additional material examined.

Brazil: *Maranhão/Piaui* border, 1 immature, C. E. V. Toledo, February 2010 (MZSP 36880).

#### Description.

Holotype female (IBSP unnumbered). Carapace 6.2 long, 6.0 wide, chelicerae 2.9. Legs (femur, patella, tibia, metatarsus, tarsus, total): I: 4.1, 2.9, 2.8, 2.4, 1.9, 14.1. II: 4.0, 2.8, 2.8, 2.2, 1.7, 13.5. III: 4.1, 2.6, 2.5, 2.2, 1.8, 13.2. IV: 4.9, 2.7, 3.3, 2.9, 1.8, 15.6. Palp: 3.4, 2.2, 1.8, –, 2.3, 9.7. Mid-widths (lateral): femora I –IV = 1.4, 1.5, 1.2, 1.2, palp = 1.0; patellae I–IV = 1.4, 1.4, 1.3, 1.2, palp = 1.1; tibiae I–IV = 1.3, 1.5, 1.2, 1.2, palp = 1.3; metatarsi I–IV = 1.2, 1.3, 0.9, 0.9; tarsi I–IV = 1.2, 1.2, 1.0, 0.9, palp = 1.2. Abdomen 8.3 long, 5.5 wide. Spinnerets: PMS, 0.9 long, 0.5 wide, 0.3 apart; PLS, 1.3 basal, 0.7 middle, 0.5 distal; mid-widths (lateral), 0.9, 0.6, 0.5, respectively. Carapace: length to width 1.03. Fovea 1.4 wide. Eyes: tubercle 0.3 high, 1.2 long, 1.7 wide. Clypeus 0.3. Anterior eye row procurved, posterior slightly recurved. Eyes size and inter-distances: AME 0.4, ALE 0.5, PME 0.2, PLE 0.4, AME–AME 0.3, AME–ALE 0.2, AME–PME 0.2, ALE–ALE 1.1, ALE–PME 0.4, PME–PME 1.0, PME–PLE 0.1, PLE–PLE 1.3, ALE–PLE 1.3, AME–PLE 0.2. Ratio of eye group width to length 2.0. Maxillae: length to width: 1.9. Cuspules: 69 spread over ventral inner heel. Labium: 0.8 long, 1.3 wide, with 80–90 cuspules spaced by one diameter from each other on the anterior third. Labio-sternal groove shallow, flat, sigilla not evident. Chelicerae: basal segments with six teeth having similar size and well spaced from each other. Sternum: 2.7 long, 2.8 wide. Legs: leg formula: IV I II III. Scopula: tarsi I–III fully scopulate, IV divided by row of 6–7 setae. Metatarsi I–II fully scopulate; III 4/5, IV 1/3 distal scopulate. IV divided by six wide row of setae. Spines absent on all legs and palps. Urticating hairs type II (0.33 long, 0.01 wide) on the abdomen dorsum. Genitalia: paired spermathecae diverging basally and curving abruptly inwards medially (Fig. 15). Color pattern: carapace, chelicerae and dorsum of legs and palps dark brown with light brown hairs. Coxae, labium, maxilla and legs ventrally brown. Sternum darker. Longitudinal stripes on dorsum of femora, patellae, tibiae and metatarsi light brown. Distal femora, patellae, tibiae and metatarsi with white rings. Abdomen dorsally black with two series of four red spots extending laterally. Abdomen ventrally black with three transversal grayish stripes. Carapace covered with very long, yellow, stiff setae in cephalic region and in front of eye tubercle. Same type of yellow setae on dorsum of chelicerae, in internal half area and in most anterior dorsal region of abdomen. Rest of abdomen dorsum has some long, scattered, white setae (Fig. 25).

#### Description.

Paratype male (IBSP unnumbered). Carapace 6.5 long, 6.1 wide, chelicerae 2.8. Legs (femur, patella, tibia, metatarsus, tarsus, total): I: 7.0, 3.2, 5.3, 4.4, 2.7, 22.6. II: 6.4, 3.1, 4.8, 4.0, 2.3, 20.6. III: 5.7, 2.8, 4.2, 4.0, 2.4, 19.1. IV: 6.9, 2.9, 5.7, 5.8, 2.4, 23.7. Palp: 3.5, 2.0, 2.6, –, 1.1, 9.2. Mid-widths (lateral): femora I–IV = 1.2, 1.4, 1.3, 1.1, palp = 0.9; patellae I–IV = 1.2, 1.3, 1.2, 1.3, palp = 1.1; tibiae I–IV = 1.2, 1.0, 1.1, 0.9, palp = 1.1; metatarsi I–IV = 0.9, 0.9, 0.9, 0.8; tarsi I–IV = 1.0, 0.9, 1.0, 0.8, palp = 1.0. Abdomen 6.4 long, 4.0 wide. Spinnerets: PMS, 0.7 long, 0.4 wide, 0.1 apart; PLS, 0.8 basal, 0.5 middle, 0.6 distal; mid-widths (lateral), 0.8, 0.7, 0.5, respectively. Carapace: length to width 1.06. Fovea 0.9 wide. Eyes: tubercle 0.4 high, 1.2 long, 1.7 wide. Clypeus 0.1. Anterior eye row procurved, posterior straight. Eye sizes and inter-distances: AME 0.4, ALE 0.4, PME 0.2, PLE 0.3, AME–AME 0.3, AME–ALE 0.2, AME–PME 0.1, ALE–ALE 1.2, ALE–PME 0.2, PME–PME 1.0, PME–PLE 0.1, PLE–PLE 1.4, ALE–PLE 0.1, AME–PLE 0.3. Ratio of eye group width to length 2.5. Other characters as in female, except: maxillae: length to width: 1.4. Cuspules: 45 spread over ventral inner heel. Labium: 0.7 long, 1.2 wide, with 70–80 cuspules. Chelicerae: basal segments with seven teeth. Sternum: 3.0 long, 2.9 wide. Scopula: tarsi I–IV fully scopulate, IV with a few sparse setae. Metatarsi I–II 4/5 scopulate; III 1/2 distal scopulate; IV 1/3 distal scopulate. IV divided by three wide row of setae. Urticating hairs type II (0.42 to 0.68 long, 0.01 to 0.02 wide) on the abdomen dorsum. Palp: embolus 1.2 long, with a 45° curvature to the retrolateral side. Embolus basal, middle and distal width of 0.15, 0.06 and 0.05, respectively. Tegulum 0.8 long. (Figs 12–14).

#### Distribution.

Brazil, states of Tocantins, Maranhão and Piaui (Fig. 28).

#### Natural history.

No available data. The male was collected by pitfall trap, the female was taking during a faunal rescue in a flooded area, and the immature in fossil tree samples coming from states of Maranhão and Piauí border.

### Pachistopelma

Pocock, 1901

http://species-id.net/wiki/Pachistopelma

Figs 29 [Fig F7] [Fig F8] [Fig F9] [Fig F10] [Fig F11] [Fig F12] [Fig F13] [Fig F14] –80

Pachistopelma Pocock 1901:548; Mello-Leitão 1923:336; Roewer 1942:256; Raven 1985:119; Platnick 2012.
Pachystopelma : Simon 1903:959; Petrunkevitch 1911:82.

#### Type species.

*Pachistopelma rufonigrum* Pocock, 1901, by original designation.

#### Diagnosis.

Male and female *Pachistopelma* differ from most other aviculariines, except *Ephebopus*, *Tapinauchenius* and *Psalmopoeus* by the straight to sligthly procurved first eye row (Fig. 37). *Pachistopelma* males differ from these genera by having a spinose spur on tibia I and females differ by having a dorso-ventrally flattened abdomen in combination with paired long spermathecae with a slight curvature medially and lacking constrictions or lobes. Additionally, males and females differ from these genera by both the absence of leg spines and the presence of urticating type II hair on the abdomen dorsum (except in mature females, that lack them).

#### Description.

**Figures 29–33.**
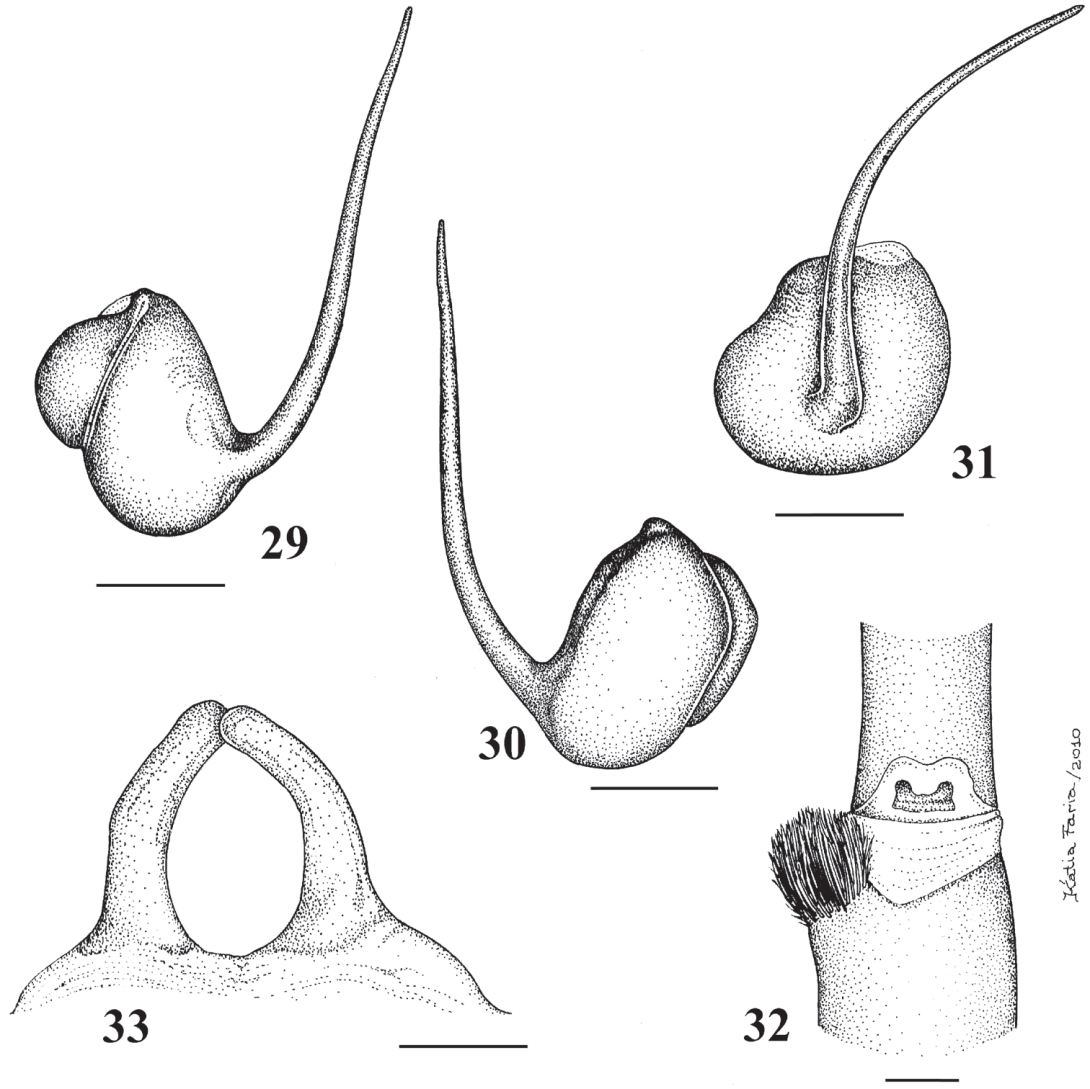
*Pachistopelma rufonigrum* Pocock, 1901 **29–32** male (MNRJ 06247), left palpal bulb **29** prolateral **30** retrolateral **31** frontal **32** male tibial spur of left leg I **33** female (MNRJ 06246) spermathecae. Scale bar = 1mm.

**Figures 34–39. F7:**
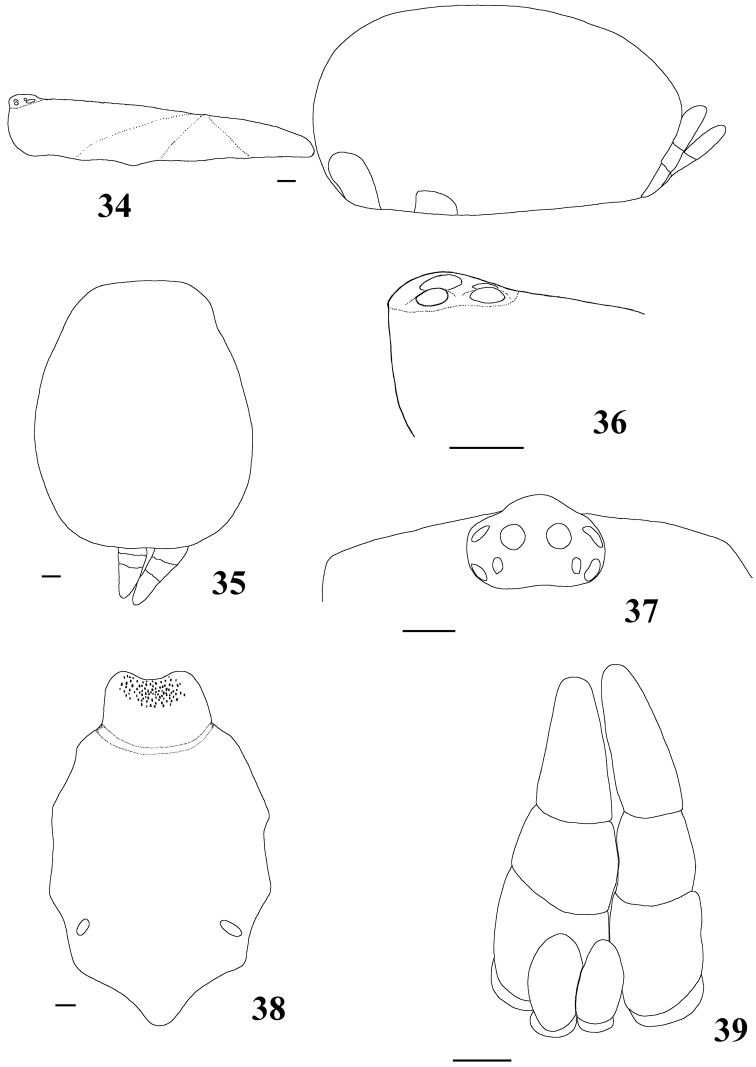
*Pachistopelma rufonigrum* Pocock, 1901, female (MNRJ 06246) **34** carapace and abdomen, lateral **35** abdomen, dorsal **36–37** eye tubercle **36** lateral **37** dorsal **38** labium and sternum **39** spinnerets, ventral. Scale bar = 1mm.

Carapace longer than wide; cephalic region low (mainly in female) (Fig. 34). Cephalic and thoracic striae hardly distinct. Fovea shallow, straight. Chelicerae without rastellum. Eye tubercle low (mainly in female), wider than long (Fig. 36). Clypeus absent (Fig. 37). Anterior eye row straight in female, slightly procurved in male (Fig. 37). Labium wider than long, with 80–150 cuspules concentrated on anterior half. Maxillary lyra absent. Maxilla subrectangular, anterior lobe distinctly produced into conical process, inner angle bearing numerous cuspules (130–200). Sternum longer than wide. Posterior angle not separating coxae IV. Posterior sigilla submarginal, less than one diameter from margin. Leg formula: IV I II III. Clavate trichobothria on distal 2/3 of tarsi I–IV. STC of males and femalewithout teeth. Tarsi I–IV fully scopulated. Scopulae of tarsi and metatarsi I–II extended very laterally giving them a spatulate appearance. Femur IV without retrolateral scopula. Legs lacking spines. Posterior median spinneret with distal article digitiform (Fig. 39). Abdomen dorso-ventrally flattened in female (Figs 34–35). Stridulatory setae absent. Male spur on tibia I, consists of a low elevation with numerous spiniform setae on its tip (Fig. 33). Male palpal bulb globose narrowing abruptly forming long slender embolus, 3 times longer than tegulum length, with a curvature of roughly 45° to retrolateral side, keels absent, tegulum without prolateral depression (Figs 29–31). Two long, uniform, weakly sclerotized spermathecae with slight curvature in their middle (Fig. 32). Cymbium with a short spiniform process (Fig. 49). Type II of urticating hair on abdomen dorsum of immatures and males, absent in mature females.

**Figures 40–45. F8:**
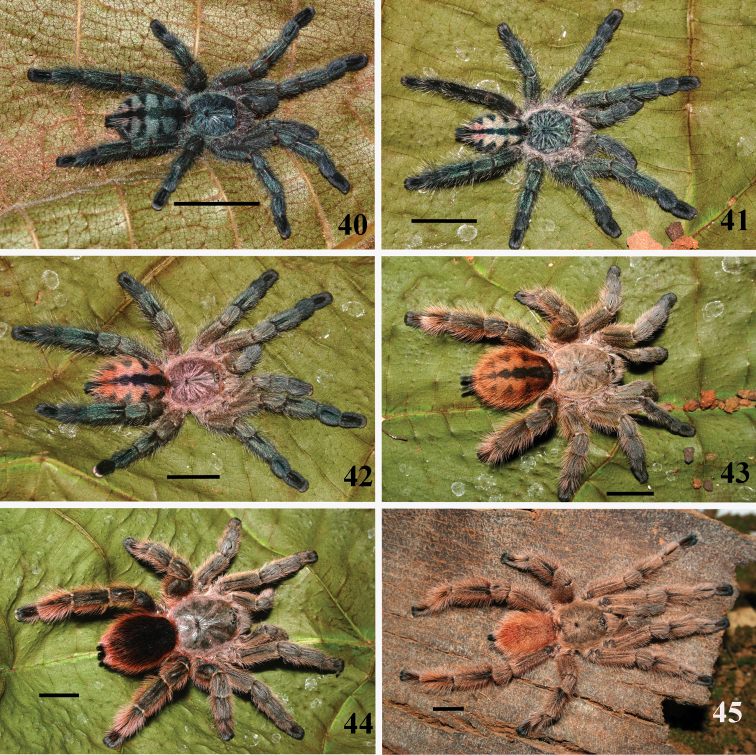
*Pachistopelma rufonigrum* Pocock, 1901, habitus **40–43** immatures in progression **44**  female **45** male, all from E.E. Murici, Murici, state of Alagoas. Photos: R. Bertani. Scale bar = 10 mm.

**Figures 46–51. F9:**
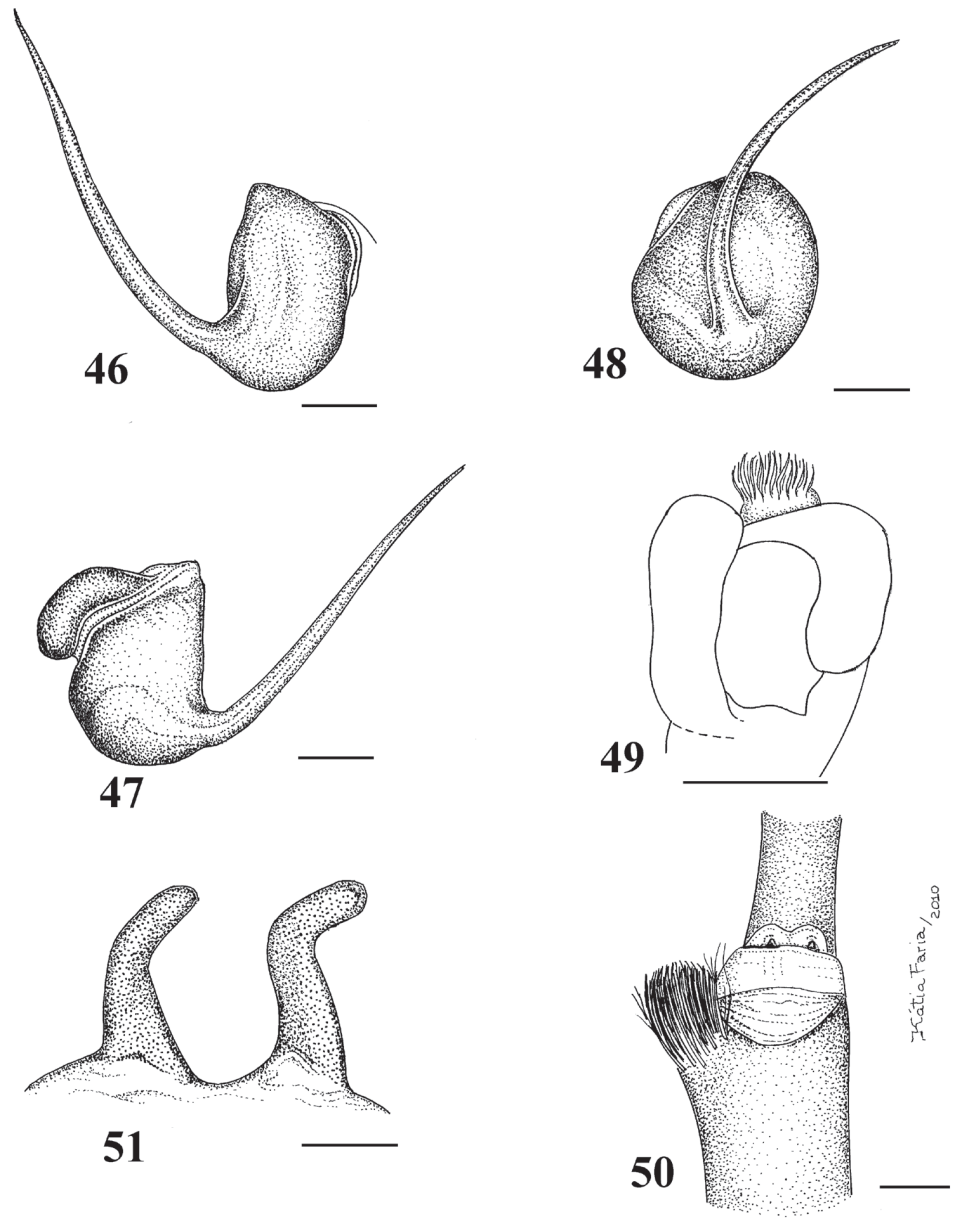
*Pachistopelma bromelicola* sp. n. **46–50** male, holotype (MNRJ 06241) left palpal bulb **46** retrolateral, **47** prolateral, **48** frontal **49** male cymbium showing protuberance **50** male tibial spur of left leg I **51** female paratype (MNRJ 06242) spermathecae. Scale bar = 1mm.

#### Species included.

*Pachistopelma rufonigrum* Pocock, 1901 (Figs 29–45, 58–61, 67–68, 76–77), *Pachistopelma bromelicola* sp. n. (Figs 46–57, 62–68, 75, 78–80).

**Figures 52–57. F10:**
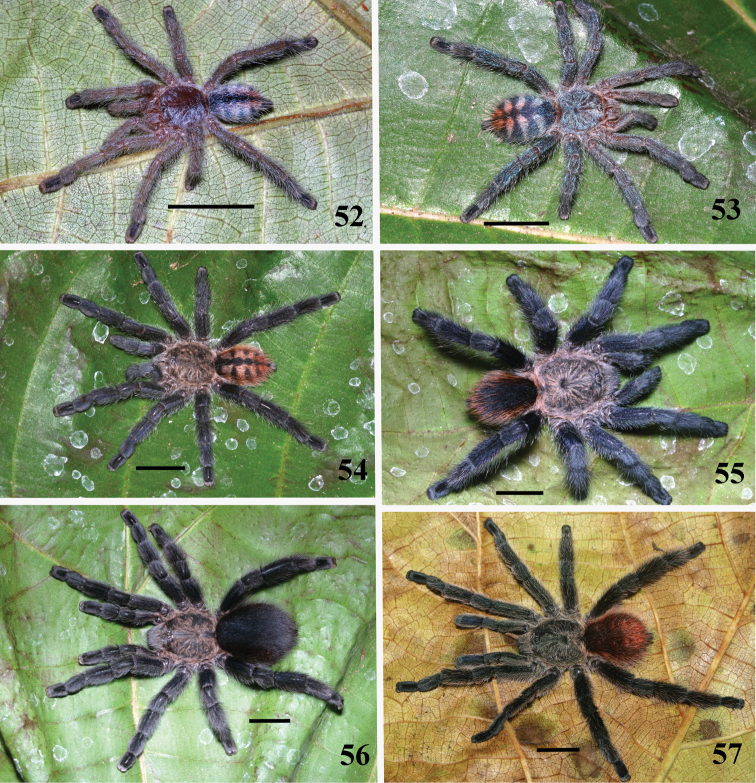
*Pachistopelma bromelicola* sp. n., habitus **52–55** immatures in progression **56** female **57** male, all from RPPN Sapiranga, Mata de São João, state of Bahia, except male, from Jeremoabo, state of Bahia. Photos: R. Bertani. Scale bar = 10 mm.

**Figures 58–65. F11:**
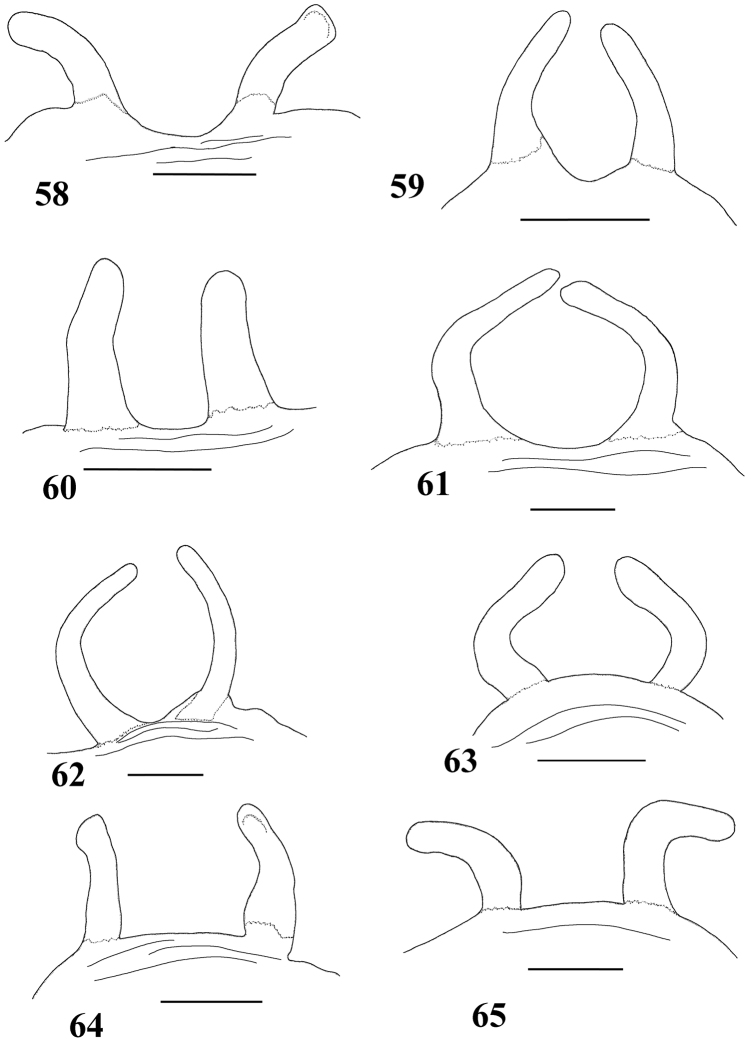
Variation in spermathecae. **58–60** *Pachistopelma rufonigrum* Pocock, 1901 **58** Alhandra, state of Paraiba (MZSP 10839) **59** Mamanguape, state of Paraíba (IBSP 9756) **60** Igarassu, state of Pernambuco (MZSP 10862) **61–65** *Pachistopelma bromelicola* sp. n. **61** Nossa Senhora da Glória, state of Sergipe (IBSP ref 28482) **62–64** Santo Amaro das Brotas, state of Sergipe (MZSP 10847, 10847, 10846) **65** Maracás, state of Bahia (IBSP 7889). Scale bar = 1mm.

**Figures 66–67. F12:**
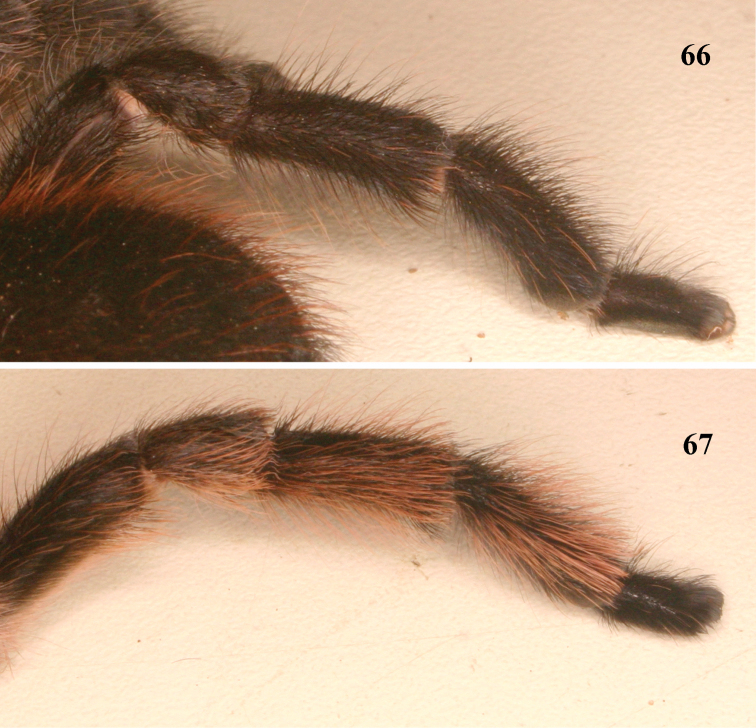
Female leg IV retrolateral view **66** *Pachistopelma bromelicola* sp. n. **67** *Pachistopelma rufonigrum* Pocock, 1901.

#### Distribution and habitat.

**Figures 69–74. F14:**
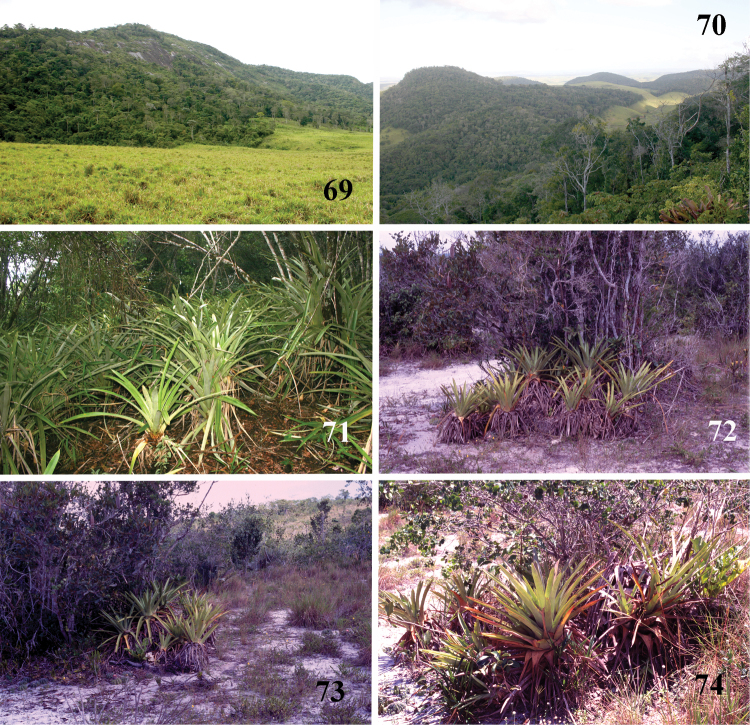
*Pachistopelma* spp. habitats **69–71** E. E. Murici, Murici, state of Alagoas **69** general view of a hill covered with Brazilian Atlantic rainforest and showing rocky formation on its tip, lower, yellowish area is a secondary grassland **70** same, viewed from its tip and showing bromeliads on the lower right of the photo **71** details of the rocky area with bromeliads intermixed with Brazilian Atlantic rainforest **72–74** Itabaiana National Park, Areia Branca, state of Sergipe, sandy areas resembling “restinga” vegetation with bromeliad “islands”. Photos: R. Bertani.

**Figures 75–80.**
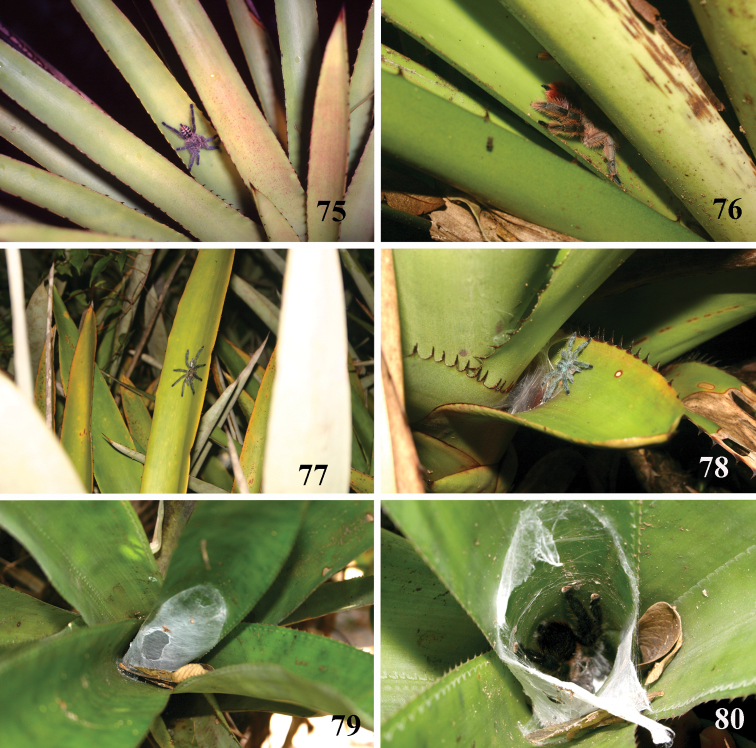
*Pachistopelma* spp. microhabitats **75, 79–80** *Pachistopelma bromelicola* sp. n. **75** Itabaiana National Park, Areia Branca, state of Sergipe **79–80** RPPN Sapiranga, São João da Mata, state of Bahia **79** closed retreat has a female with eggsac **80** same retreat opened **76–78** *Pachistopelma rufonigrum* Pocock, 1901, E. E. Murici, Murici, state of Alagoas. Photos: R. Bertani.

Northeastern Brazil, from the state of Rio Grande do Norte [6°22'S] to state of Bahia [13°25'S], mostly in the coastal region (Fig. 68). Both species of *Pachistopelma* inhabit tank bromeliads exclusively, e. g. *Aechmea aquilega* (Dias et al. 2000, Santos et al. 2004); *Hohenbergia stellata*, *Hohenbergia ridley* (Dias et al. 2000); *Hohenbergia ramageana* and *Avicularia lingulata* (Santos et al. 2004) (Figs 69–80) which can be found in very distinct habitats such as restinga (Figs 72–74), caatinga or even rainforest (Figs 69–71). In the last case, the spiders were in bromeliads that grow mainly on rocky outcrops exposed to direct and intense sunlight.

**Figure F13:**
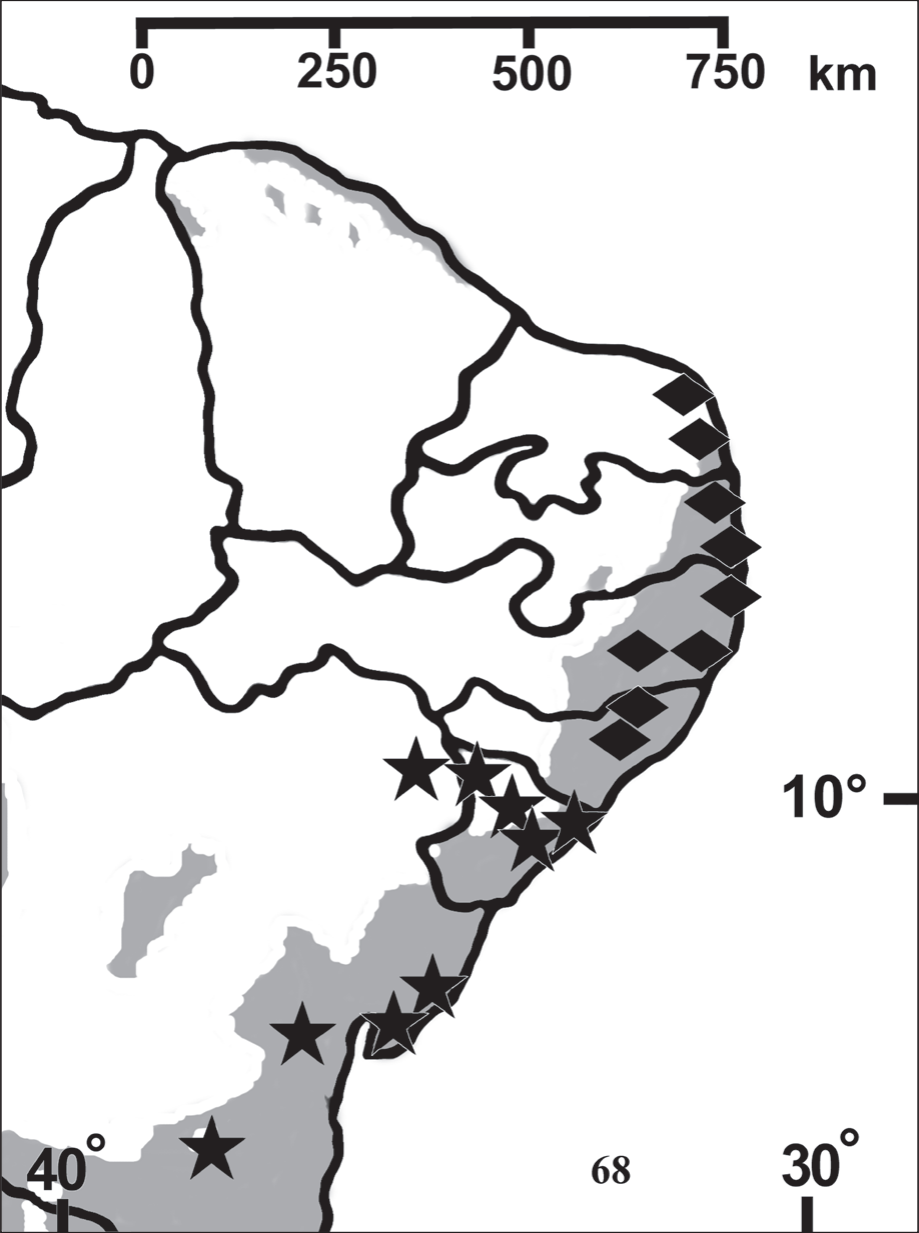
**Figure 68.** Map showing records of *Pachistopelma* species on Northeastern Brazil. Losangle = *Pachistopelma rufonigrum*, star = *Pachistopelma bromelicola* sp. n. The gray area represents the approximate original distribution of Brazilian Atlantic rainforest. White area represents open environments (cerrado and caatinga).

#### Color pattern ontogeny.

*Pachistopelma* juveniles possess a metallic green general pattern, and the dorsum of abdomen having a black longitudinal central stripe and five lateral black stripes that may connect with the central stripe. Adults are brownish to pinkish or blackish, without abdominal pattern (Figs 40–45, 52–57).

#### Remarks.

*Pachistopelma concolor* Caporiacco, 1947, lectotype (herein designated), immature male, Guyana, Campo di Marlissa, 31 December (MZUF 507); paralectotype, immature, Guyana, Campo I Demerara (MZUF 505), deposited in Museo Zoologico “La Specola”, Firenze, examined. Specimens have spines on apex of tibiae and metatarsi, lack urticating hair both on abdomen dorsum as well as on prolateral distal femur of palp, first ocular eye row is almost straight, and maxillary lyra is absent. This combination of characters is diagnostic for *Tapinauchenius*, and, therefore, *Pachistopelma concolor* Caporiacco, 1947 is transferred to *Tapinauchenius concolor* (Caporiacco, 1947) comb. n.

#### Key to species of *Pachistopelma*

**Table:** 

1	Metatarsus IV incrassate, with stiff setae (Fig. 66); general coloration dark (Fig. 56), male with longer setae on legs whitish and abdomen dorsum reddish (Fig. 57)	*Pachistopelma bromelicola* sp. n.
–	Metatarsus IV not incrassate, with long and flexible reddish setae (Fig. 67); general coloration dark brown with pinkish setae (Figs 44–45)	*Pachistopelma rufonigrum*

### Pachistopelma rufonigrum

Pocock, 1901

http://species-id.net/wiki/Pachistopelma_rufonigrum

Figs 29 [Fig F7] –45 , 58–60 , 67 –68 , 76–77

Pachistopelma rufonigrum Pocock 1901:548; Mello-Leitão 1923:337; Roewer 1942:256; Platnick 2012.
Pachystopelma rufonigrum : Simon 1903:959; Petrunkevitch 1911:82.
Avicularia pulchra Mello-Leitão 1933:171, f. 33. **syn. n.**
Avicularia recifiensis Struchen and Brändle 1996:2, f 1–4. **syn. n.**

#### Diagnosis.

Males and females differ from *Pachistopelma bromelicola* sp. n. by the slender metatarsi IV without stiff bristles (Fig. 67) and legs pinkish with black tarsi (Figs 43–45).

#### Types.

Lectotype (herein designated) a dissected adult female and 8 paralectotypes (herein designated), comprising 4 females, 2 males and 2 immatures fom Iguarassu, Brazil, G. A. Ramage, BMNH 1888.47, examined.

#### Additional material examined.

BRAZIL: *Rio Grande do Norte*: Baia Formosa [6°22'S, 35°00'W], inside bromeliads, 1 female, 8 immatures, A. L. Castro, 29 January 1964 (MNRJ 12922); Natal, road to Zumbi [5°42'S, 35°16'W], 1 female, 3 immatures, M. Aranha, M. Uvana, M. C. B. Pereira, H. R. Silva, 1 February 1989 (MNRJ 13750); *Paraíba*: Alhandra [7°26'S, 34°54'W], inside bromeliads, 1 female, 44 immatures, Exp. ABC MZUSP, 8 May 1971 (MZSP 10839); same data, 1 female, 2 immatures (MZSP 28579); Igarassú [7°50'S, 34°54'W], 2 females, 4 immatures, Vanzolini, 7 June 1963 (MZSP 10862); Mamanguape, Reserva Biológica de Mamanguape [6°47'S, 34°59'W], inside bromeliads, 1 male, 2 females, Cascone and Alonso, 31 October 1982 (IBSP 9756); same data, 4 immatures (IBSP 14276); *Pernambuco*: Agrestina, Serra da Quitéria [8°27'S, 35°56'W], inside bromeliads, 1 female, 2 immatures, Exp. ABC MZUSP, 15 May 1971 (MZSP 28580); Rio Formoso, Estação Ecológica de Saltinho [8°43'S, 35°10'W], 1 immature, 12 May 1969 (IBSP 3986B); *Alagoas*: Murici, Estação Ecológica de Murici, Triunfo (9°14'9.52"S, 35°48'0.25"W), 245 m.a.s.l., inside bromeliads, 3 immatures, R. Bertani, R. H. Nagahama, D. R. M Ortega, 11 August 2006 (MNRJ 06244); same data, 5 immatures, 2 males, 1 female, 12 August 2006 (MNRJ 06245); same data, 6 immatures, 1 male, 5 females, 13 August 2006 (MNRJ 06246); Jitituba (9°14'9.52"S, 35°48'0.25"W), 295 m.a.s.l., inside bromeliads, 2 males, 3 females, R. Bertani, R. H. Nagahama, D. R. M Ortega, 14 August 2006 (MNRJ 06247); Fazenda Santa Fé (9°26'1.83"S, 35°50'3.93"W), 362 m.a.s.l., inside bromeliads, 1 male, 2 females, R. Bertani, R. H. Nagahama, D. R. M. Ortega, 15 August 2006 (MNRJ 06248); Passo do Camaragibe, Fazenda Santa Justina [9°14'S, 35°29'W], 2 females, 1 immature, H. R. Silva and C. A. Caetano, 13–18 January 1988 (MNRJ 1781).

#### Redescription.

Female (MNRJ 06246, Al 1110) from Brazil, state of Alagoas, Murici, Estação Ecológica de Murici. Carapace 15.1 long, 13.3 wide, chelicerae 7.3. Legs (femur, patella, tibia, metatarsus, tarsus, total): I: 9.7, 6.6, 7.3, 6.8, 4.1, 34.5. II: 9.2, 6.3, 6.8, 6.5, 3.9, 32.7. III: 8.8, 5.5, 6.3, 6.8, 4.0, 31.4. IV: 11.0, 6.1, 9.1, 9.7, 4.3, 40.2. Palp: 7.3, 4.7, 4.8, –, 5.1, 21.9. Mid-widths (lateral): femora I–IV=2.8, 2.8, 3.0, 3.1, palp=2.4; patellae I–IV=2.7, 2.8, 2.8, 2.9, palp=2.4; tibiae I–IV=2.4, 2.5, 2.4, 2.8, palp=2.3; metatarsi I–IV=1.9, 1.9, 1.8, 1.7; tarsi I–IV=1.8, 1.8, 1.7, 1.9, palp=2.0. Abdomen 14.5 long, 12.0 wide. Spinnerets: PMS, 1.8 long, 0.7 wide, 0.3 apart; PLS, 2.7 basal, 2.1 middle, 2.6 distal; mid-widths (lateral), 1.6, 1.3, 0.8, respectively. Carapace: length to width 1.13; Fovea 1.6 wide. Eyes: tubercle 0.3 high, 1.7 long, 2.6 wide. Anterior eye row straight, posterior slightly recurved. Eye sizes and inter-distances: AME 0.6, ALE 0.6, PME 0.3, PLE 0.5, AME–AME 0.4, AME–ALE 0.3, AME–PME 0.3, ALE–ALE 1.7, ALE–PME 0.3, PME–PME 1.3, PME–PLE 0.2, PLE–PLE 1.8, ALE–PLE 0.3, AME–PLE 0.5. Ratio of eye group width to length 2.2. Maxillae: length to width: 1.7. Cuspules: 150–200 spread over ventral inner heel. Labium: 1.8 long, 2.7 wide, with 100–120 cuspules spaced by one diameter from each other on the anterior third. Labio-sternal groove shallow, flat, sigilla not evident. Chelicerae: basal segments with ten teeth decreasing in size from distal to basal portion. Sternum: 7.4 long, 6.2 wide. Sigilla: three pairs, ellipsoid, less than one diameter from margin. Scopula: tarsi I–IV fully scopulate, IV divided by five wide row of setae. Metatarsi I 3/4 scopulate; II 2/3 scopulate; III 1/2, IV 1/3 distal scopulate. IV divided by three wide row of setae. Urticating hair absent. Genitalia: paired long, uniform, weakly sclerotized spermathecae with a slight curvature medially (Fig. 32). Color pattern: carapace and chelicerae brown, covered with golden hairs intermixed with metallic pinkish hairs. Legs and palps dark brown, covered with dark hairs having metallic green/blue iridescence. Coxae, labium, maxilla and sternum black. Longitudinal stripes on dorsum of femora, patellae, tibiae and metatarsi narrow, whitish. Distal femora, patellae, tibiae and metatarsi rings absent. Abdomen dorsum black with long reddish hairs mainly on lateral and posterior regions. Abdomen ventrally black (Fig. 44).

#### Redescription.

Male (MNRJ 06245, Al 1100) fromBrazil, state of Alagoas, Murici, Estação Ecológica de Murici. Carapace 12.7 long, 11.4 wide, chelicerae 5.6. Legs (femur, patella, tibia, metatarsus, tarsus, total): I: 9.8, 5.5, 7.5, 7.8, 4.8, 35.4. II: 9.3, 5.6, 7.2, 7.2, 4.0, 33.3. III: 8.6, 4.6, 6.5, 7.3, 3.9, 30.9. IV: 10.4, 5.5, 9.1, 10.4, 3.9, 39.3. Palp: 6.8, 4.1, 5.3, –, 1.8, 18.0. Mid-widths (lateral): femora I–IV=2.2, 2.3, 2.6, 2.8, palp=1.8; patellae I–IV=2.3, 2.2, 2.3, 2.3, palp=1.7; tibiae I–IV=1.8, 1.9, 1.9, 2.1, palp=1.6; metatarsi I–IV=1.1, 1.1, 1.3, 1.1; tarsi I–IV=1.2, 1., 1.2, 1.3, palp=1.5. Abdomen 13.9 long, 9.2 wide. Spinnerets: PMS, 1.2 long, 0.5 wide, 0.4 apart; PLS, 2.1 basal, 1.4 middle, 2.0 distal; mid-widths (lateral), 1.0, 0.9, 0.6, respectively. Carapace: length to width 1.11; Fovea: 0.9 wide. Eyes: tubercle 0.4 high, 1.7 long, 2.3 wide. Anterior eye row slightly procurved, posterior slightly recurved. Eye sizes and inter-distances: AME 0.4, ALE 0.6, PME 0.3, PLE 0.4, AME–AME 0.4, AME–ALE 0.3, AME–PME 0.2, ALE–ALE 1.6, ALE–PME 0.5, PME–PME 1.1, PME–PLE 0.1, PLE–PLE 1.5, ALE–PLE 0.3, AME–PLE 0.5. Ratio of eye group width to length 1.9. Other characters as in female, except: labium: 1.5 long, 2.0 wide, with ca. 80 cuspules spaced by one diameter from each other on the anterior third center. Chelicerae: basal segments with nine teeth decreasing in size from distal to basal portion. Sternum: 6.0 long, 4.9 wide. Sigilla: three pairs, small, rounded, first and last pairs 1.5 diameter from margin, second less than half diameter from margin. Scopula: metatarsi I–II 4/5 scopulate; III 1/3 distal scopulate; IV 1/4 distal scopulate. IV divided by five wide row of setae. Tibial spur 0.7 high, 1.3 wide; with numerous spiniform setae on the tip (Fig. 33). Metatarsus I straight. Urticating hairs type II (0.51 to 0.58 long, 0.012 wide) on the abdomen dorsum. Palp: embolus 3.4 long, with a 45° curvature to the retrolateral side. Embolus basal, middle and distal width of 0.3, 0.2 and 0.04, respectively. Tegulum 1.0 long, 1.7 wide (Figs 29–31). Cymbium: two subequal lobes, the prolateral one triangular in shape. Spiniform process 0.5 long, 0.6 wide on the apex. Color pattern: carapace, chelicerae, legs and palps dark brown, covered with dense layer of pinkish hairs and longer hairs of the same color. Tarsi black, metatarsi dorsum with black stripe. Coxae and sternum dark brown with pinkish hairs. Labium and maxillae dark brown. Longitudinal stripes on dorsum of femora, patellae, tibiae and metatarsi discrete, light brown. Abdomen dorsum orange with long reddish hairs. Abdomen ventrally orange with a greyish area on its center (Fig. 45).

#### Sexual dimorphism.

Females and immatures have a very low cephalic region, when compared to the males, and the abdomen is dorso-ventrally flattened in the former (Fig. 34). The eye tubercle is very low in females (Fig. 36) and immatures, and the first ocular row is straight (Fig. 37). Males possess a more developed eye tubercle and the anterior ocular row is slightly procurved. Immatures and adult males have type II urticating hairs on abdomen dorsum, which becomes lost in adult females.

#### Spermathecae variation.

A typical *Pachistopelma rufonigrum* spermatheca is weakly sclerotized, long, tapering distally, without constrictions or lobes, and slightly curved inwards (Figs 32, 59). Nevertheless, shorter (Figs 58, 60) spermathecae are not rare, as well as broader ones (Fig. 60). Most are sligthly curved inwards (Figs 32, 59) whereas a fraction are curved outwards (Fig. 58).

#### Distribution.

Brazil, from state of Rio Grande do Norte southwards to state of Alagoas, mainly in coastal region (Fig. 68).

#### Natural history.

All specimens with recorded field data indicate *Pachistopelma rufonigrum* individuals were found inside bromeliads (Bertani et al. 1994; Santos et al. 2002, 2004; this work). Field observations suggest a strict dependency on bromeliads (Santos et al. 2004), both in restinga vegetation and in restinga associated with Brazilian Atlantic rainforest. Of these, bromeliads occurring in Atlantic rainforest areas had a low occupancy rate by *Pachistopelma rufonigrum* individuals than those bromeliads in xerophylous environments (Santos et al. 2004). In Estação Ecologica Murici, Murici, state of Alagoas, Brazil, I observed several individuals exclusively inside tank bromeliads. These were 245-362 m a.s.l. in rocky outcrops marginated by Brazilian Atlantic forest. Bromeliads in this area form large patches in rocky outcrops where the soil is shallow and sunlight intense. The margin of these patches overlap with shaded areas contiguous with the forest, and, in agreement with Santos et al. 2004, specimens were found mainly in bromeliads more exposed to sunlight. The “bromeliad islands” in rocky outcrops are hundreds or thousands of meters apart, and no *Pachistopelma rufonigrum* specimens were found outside of bromeliads. Thus, these populations are probably isolated. The species is abundant, *ca*. 30 specimens were collected, and others were observed but not collected. Individuals in all developmental stages were observed during this the period (August 2006). In agreement with Santos et al. 2004, spiders were observed diving into the phytotelma water, staying there for several minutes, when disturbed.

#### Color pattern ontogeny.

Juveniles are almost completely metallic green, except for a pattern on dorsum of abdomen comprising a central longitudinal black stripe connected with five lateral black stripes (Fig. 40). In larger individuals carapace border and dorsum of chelicerae, coxae and trochantera are light brown. Dorsum of abdomen is light brown with a reddish area posteriorly. The black stripes remain (Fig. 41). In a next stage carapace is completely pink, as well as dorsum of coxae, trochantera and most femora. Remaining parts of legs retain the metalic-green. The clear part of abdomen is now of a vivid red, and the black stripes remain (Fig. 42). Subadults have carapace and legs brown with sparse pinkish long hairs. Dorsum of abdomen is still reddish, but the black stripes begin to fade (Fig. 43). Adult female is completely brown with long pinkish setae on legs, carapace and chelicerae. Abdominal pattern is lacking or very inconspicuous (Fig. 44). Adult male carapace, chelicerae and legs are brown and covered by pinkish setae, except for the tarsi and a stripe on metatarsi that are black. Abdomen is a vivid orange/red, there is no vestige of any pattern (Fig. 45).

#### Remarks.

*Avicularia pulchra* Mello-Leitão, 1933, holotype from Brazil, Pernambuco, D. Bento Pickel (MNRJ 29180), examined, is an immature specimen with a low eye tubercle, almost straight anterior row of eyes, abdomen flattened dorso-ventrally, and typical abdominal pattern, all characteristics of *Pachistopelma*. The color pattern and geographical distribution are consistent with *Pachistopelma rufonigrum*. Therefore, *Avicularia pulchra* Mello-Leitão, 1933 is transferred to *Pachistopelma* and considered a junior-synonym of *Pachistopelma rufonigrum* Pocock, 1901, syn. n.

*Avicularia recifiensis* Struchen & Brändle, 1996, holotype female (SMF 39873) and paratype male (SMF 39872), from Brazil, Recife, Martin Weber, 1993 according to original description. Specimen labels indicate holotype male and paratype female contrary to paper. The holotype female, examined, has a very low eye tubercle, straight anterior eye row, dorso-ventrally flattened abdomen, and spermathecae, though dissected, was not in the vial. Paratype male has a poorly developed tibial spur typical of the species (contrary to description that states it lacks a tibial spur), a spiniform process between cymbium lobes, and a sligthly procurved anterior eye row. All characters of both male and female agree with those of *Pachistopelma*, and color pattern and geographical distribution agree with *Pachistopelma rufonigrum*. Therefore, *Avicularia recifiensis* Struchen & Brändle, 1996 is transferred to *Pachistopelma* and considered a junior-synonym of *Pachistopelma rufonigrum* Pocock, 1901, syn. n.

### Pachistopelma bromelicola sp. n.

urn:lsid:zoobank.org:act:0D248413-EA0C-4187-8E15-C71B93A27D9E

http://species-id.net/wiki/Pachistopelma_bromelicola

Figs 46 –57 , 61 [Fig F12] –68 , 75, 78–80

Pachistopelma rufonigrum : Dias 2004:153–154; Dias et al. 2000:22–24; Dias and Brescovit 2003:13–17; 2004:789–796.

#### Diagnosis.

Males and females differ from those of *Pachistopelma rufonigrum* by the incrassate metatarsus IV with stiff bristles (Fig. 66) and blackish color of the legs (Figs 54–57).

#### Etymology.

The specific name refers to the lifestyle habits of this species, bromeliad endemism.

#### Types.

Holotype male (MNRJ 06241), Brazil, State of Bahia, Elísio Medrado, RPPN Jequitibá (12°52'3.20"S, 39°28'9.09"W), R. Bertani, C.S. Fukushima and R.H. Nagahama, 07 October 2007, collected at night, found immature inside bromeliads, matured in captivity in May 2010; Paratype female (MNRJ 06242), same data.

**Additional material examined.** BRAZIL: *Sergipe*: Areia Branca, Parque Nacional Serra de Itabaiana [10°44'S, 37°22'W], inside bromeliads, 4 males, R. Bertani, A. D. Brescovit, A. B. Bonaldo, September 1999 (IBSP 10010, 9789, 9832, 8716); 5 females, same data (IBSP 10216, 10463, 10399, 10035, 10748); 3 immatures, same data (IBSP 8644, 8599, 8525); 1 female, 1 immature, A. C. M. Fernandes, 12 November 1996 (IBSP 11762); Barra dos Coqueiros [10°54'S, 37°01'W] 1 immature, without collector, 20 February 1994 (IBSP 8087); 2 males, without data (IBSP Ref. 79901); Brejo Grande [10°25'S, 36°28'W], 1 female, no collector data, 26 October 1998 (IBSP 8085); Nossa Senhora da Gloria [10°13'S, 37°25'W], 1 female, S. Lucas, 1980 (IBSP 7892); 1 male, 1 female, same data (IBSP 7893); 1 female, same data (IBSP Ref. 28482); Pirambú [10°40'S, 36°52'W], 1 immature, without collector, 19 February 1998 (IBSP 8088); Poço Redondo [9°47'S, 37°41'W], 1 female, S. Lucas, August 1980 (IBSP 4712); Santo Amaro das Brotas [10°46'S, 37°03'W], inside bromeliad, 1 female, A. V. Alcântara, 09 September 1978 (MZSP 10846); 1 female, 2 immatures, no collector data, 7 October 1978 (MZSP 10842); 1 female, 2 immatures, no collector data, 2 June 1979 (MZSP 10843); 1 female, no collector data, 11 November 1978 (MZSP 10841); 14 females, 6 immatures, no collector data, 23 March 1978 (MZSP 10847); 1 female, no collector data, 02 July 1979 (MZSP 10845). *Bahia*: Acajutiba [11°39'S, 38°01'W], 2 females, 10 immatures, E. Boaventura, 18 April 1991 (MZSP 32179, col. Bock. 699–710); Camaçari, Praia do Jacuípe [12°42'S, 38°07'W], 1 male, L. Stabile, 7 September 2006 (IBSP 12991); Elísio Medrado, RPPN Jequitibá (12°52'3.20"S, 39°28'9.09"W), inside bromeliads, 2 males, 3 females, R. Bertani, R. H. Nagahama, C. S. Fukushima, 7 October 2007 (MZSP 36881); 1 female, 4 immatures, inside bromeliads, M. A. Freitas, April 2010 (MZSP 36882); Jeremoabo, [10°03'S, 38°20'W], 1 male, A. J. Silva, November 1989 (MZSP 32177, col. Bock. 770); 1 male, A. J. Silva (MZSP 32175, col. Bock. 787); 1 male, A. J. Silva, 15 July 1989 (MZSP 32172, col. Bock. 786); 1 male, A. J. Silva, 28 October 1989 (MZSP 32178, col. Bock. 792); 1 male, 2 females, A. J. Silva, December 1988 (MZSP 32169, col. Bock. 837–839); 3 females, A. J. Silva, 28 October 1999 (MZSP 32166, col. Bock. 827–829); 1 male, 7 females, 2 immatures, A. J. Silva, December 1988 (MZSP 32163, col. Bock. 840–849); 2 males, 6 females, 1 immature, A. J. Silva, 16 January 1989 (MZSP 32170, col. Bock. 861–869); 2 males, 14 females, 1 immature, A. J. Silva, 29 June 1989 (MZSP 32171, col. Bock. 711–713, 716, 748–755, 768–769, 772–774); 2 males, 8 females, 1 immature, A. J. Silva, December 1988 (MZSP 32167, col. Bock. 850–859); 6 females, A. J. Silva, 16 January 1989 (MZSP 32165, col. Bock. 880–884); 2 males, 6 females, A. J. Silva, 16 January 1989 (MZSP 32168, col. Bock. 870, 872–875, 877–879); 5 females, A. J. Silva, 28 October 1989 (MZSP 32164, col. Bock. 830, 832, 834–836); Maracás [13°25'S, 40°26'W], 1 female, Werner, November 1965 (IBSP 7889); Mata de São João, RPPN Sapiranga (12°34'0.58"S, 38°02'3.38"W), inside bromeliads, 2 females, 1 immature, R. Bertani, R. H. Nagahama, C. S. Fukushima, 1 October 2007 (MNRJ 06243); Minuim [9°50'S, 38°05'W], 1 male, without colector, 06 June 1987 (MZSP 32176, col. Bock. 714); Salvador, Itapoã [12°57'S, 38°21'W] 1 male, 1 female, A. Travassos, 1951 (IBSP 2369); inside bromeliads, 2 immatures, Vanzolini and Rebouças, December 1962 (MZSP 10840); 1 immature, same data and colectors (MZSP 10863); 1 male, 1 female, 1 immature, Vanzolini, 3 June 1963 (MZSP 4992); Ondina [13°00'S, 38°30'W], 1 female, T. B. Nunes, December 1982 (IBSP 7905); Santa Brigida [9°43'S, 38°07'W], 1 female, J. P. Carvalho, 23 October 1987 (MZSP 32174, col. Bock. 789).

#### Description.

Holotype male (MNRJ 06241). Carapace 11.6 long, 11.4 wide, chelicerae 5.3. Legs (femur, patella, tibia, metatarsus, tarsus, total): I: 9.8, 6.0, 7.6, 8.0, 4.6, 36.0. II: 9.5, 5.4, 7.1, 7.7, 4.1, 33.8. III: 8.5, 4.7, 7.2, 7.6, 4.1, 32.1. IV: 11.0, 5.4, 9.5, 10.7, 4.2, 40.8. Palp: 7.3, 4.2, 5.6, –, 2.3, 19.4. Mid-widths (lateral): femora I–IV = 2.4, 2.4, 2.6, 2.7, palp= 1.7; patellae I–IV = 2.2, 2.1, 2.2, 2.3, palp = 1.7; tibiae I–IV = 2.0, 1.9, 1.9, 2.1, palp = 1.7; metatarsi I–IV = 1.3, 1.2, 1.2, 1.4; tarsi I–IV = 1.2, 1.2, 1.2, 1.2, palp = 1.3. Abdomen 13.9 long, 9.4 wide. Spinnerets: PMS, 1.2 long, 0.4 wide, 0.4 apart; PLS, 2.2 basal, 1.1 middle, 1.5 distal; mid-widths (lateral), 1.0, 0.7, 0.6, respectively. Carapace: length to width 1.01; Fovea 1.1 wide. Eyes: tubercle 0.3 high, 1.2 long, 1.9 wide. Anterior eye row slightly procurved, posterior slightly recurved. Eye sizes and inter-distances: AME 0.4, ALE 0.4, PME 0.2, PLE 0.4, AME–AME 0.3, AME–ALE 0.3, AME–PME 0.2, ALE–ALE 1.5, ALE–PME 0.3, PME–PME 1.1, PME–PLE 0.2, PLE–PLE 1.6, ALE–PLE 0.3, AME–PLE 0.5. Ratio of eye group width to length 2.1. Maxillae: length to width: 1.6. Cuspules: 130–150 spread over ventral inner heel. Labium: 1.2 long, 1.9 wide, with *ca*. 90 cuspules spaced by one diameter from each other on the anterior third center. Labio-sternal groove shallow, flat, with two sigilla. Chelicerae: basal segments with eigth teeth decreasing in size from distal to basal portion. Sternum: 5.6 long, 4.7 wide. Sigilla: three pairs, small, ellipsoid, less than one diameter from margin. Scopula: tarsi I–IV fully scopulate, IV divided by four wide row of setae. Metatarsi I 4/5 scopulate; II 2/3 scopulate; III 1/2 distal scopulate; IV 1/3 distal scopulate. IV divided by five wide row of setae. Tibial spur 0.8 high, 1.4 wide; with numerous spiniform setae on tip (Fig. 51). Metatarsus I straight. Urticating hairs type II (0.63 to 1.0 long, 0.012 to 0.016 wide) on the abdomen dorsum. Palp: embolus 2.9 long, with a 45° curvature to the retrolateral side. Embolus basal, middle and distal width of 0.3, 0.2 and 0.08, respectively. Tegulum 1.0 long, 1.8 wide. (Figs 46–48). Cymbium: two subequal lobes, the prolateral one triangular in shape. Spiniform process 0.3 long, 0.4 wide on the apex (Fig. 49). Color pattern: carapace and chelicerae dark brown, covered with golden hairs. Legs and palps black, longer hairs with distal half light brown. Coxae, labium, maxilla and sternum black. Longitudinal stripes on dorsum of femora, patellae, tibiae and metatarsi inconspicuous. Distal femora, patellae, tibiae and metatarsi without rings. Abdomen dorsum orange with long reddish hairs. Abdomen ventrally grayish (Fig. 57).

#### Description.

Paratype female (MNRJ 06242). Carapace 15.7 long, 14.8 wide, chelicerae 7.3. Legs (femur, patella, tibia, metatarsus, tarsus, total): I: 10.1, 6.7, 7.6, 6.7, 4.0, 35.1. II: 9.2, 6.5, 6.6, 6.6, 3.8, 32.7. III: 8.9, 5.6, 6.7, 7.0, 3.9, 32.1. IV: 11.0, 6.5, 9.7, 9.8, 4.1, 41.1. Palp: 7.2, 5.1, 4.8, –, 4.9, 22.0. Mid-widths (lateral): femora I–IV = 2.8, 3.1, 3.4, 3.4, palp = 2.2; patellae I–IV = 2.9, 2.8, 3.0, 3.0, palp=2.3; tibiae I–IV = 2.7, 2.6, 2.8, 3.1, palp = 2.3; metatarsi I–IV = 2.0, 1.9, 2.0, 2.4; tarsi I–IV = 1.9, 1.9, 1.9, 1.9, palp = 2.0. Abdomen 17.4 long, 13.3 wide. Spinnerets: PMS, 1.7 long, 0.8 wide, 0.6 apart; PLS, 2.6 basal, 2.1 middle, 2.3 distal; mid-widths (lateral), 1.4, 1.1, 0.9, respectively. Carapace: length to width 1.06. Fovea 1.6 wide. Eyes: tubercle 0.3 high, 1.8 long, 2.6 wide. Anterior eye row straight, posterior slightly recurved. Eye sizes and inter-distances: AME 0.5, ALE 0.5, PME 0.3, PLE 0.4, AME–AME 0.4, AME–ALE 0.3, AME–PME 0.3, ALE–ALE 1.7, ALE–PME 0.4, PME–PME 1.3, PME–PLE 0.2, PLE–PLE 2.0, ALE–PLE 0.4, AME–PLE 0.5. Ratio of eye group width to length 2.4. Maxillae: length to width: 1.7. Cuspules: 150–200 spread over ventral inner heel. Labium: 2.0 long, 2.7 wide, with *ca*. 150 cuspules spaced by one diameter from each other on the anterior third. Labio-sternal groove without evident sigilla. Chelicerae:basal segments with eleven teeth decreasing in size from distal to basal portion.Sternum: 7.4 long, 6.4 wide. Urticating hairs on abdomen dorsum lacking. Genitalia: paired long, uniform, weakly sclerotized spermathecae with a slight curvature in their middle (Fig. 50). Color pattern: as in male, except abdomen dorsum black with sparse long reddish hairs, ventrally black (Fig. 56).

#### Distribution.

Brazil: States of Sergipe and Northern State of Bahia, mainly in coastal regions (Fig. 68).

#### Sexual dimorphism.

The cephalic region of female and immatures is very low in profile when compared with those of male, and abdomen is dorso-ventrally flattened in the former (Fig. 34). The eye tubercle is very low in female (Fig. 36) and immature, and first ocular row is straight (Fig. 37). Males have a more developed eye tubercle, the anterior ocular row is slightly procurved. Immatures and adult males have urticating hair type II on abdomen dorsally, which becomes lost in adult females.

#### Spermathecae variation.

As *Pachistopelma rufonigrum*, the typical spermatheca is weakly sclerotized, long, tapering apically, without constrictions or lobes, and slightly curved inwards (Figs 61–62). Shorter spermathecae (Figs 63–65) can be found, as well as almost straight ones (Fig. 64) and a few are curved outwards (Fig. 65). In the paratype female (MNRJ 06242), one spermatheca is curved inwards whereas the other is curved outwards (Fig. 50). Spermathecae of Figs 62–64 are from specimens of same population.

#### Natural history. 

As with *Pachistopelma rufonigrum*, all specimens examined and labeled with field data indicate they were found inside bromeliads, which agrees with field observations (Dias et al 2000; Dias and Brescovit 2003, 2004 – all misidentified as *Pachistopelma rufonigrum*). In my own field observations in Parque Nacional de Itabaiana, Areia Branca, state of Sergipe (September 1999); RPPN Sapiranga, Mata de São João, state of Bahia (October 2007) and RPPN Jequitiba, Elísio Medrado, state of Bahia (October 2007) specimens were found only inside bromeliads. The habitat in Itabaiana consists of white sandy soils with scattered shrubs, cactus and bromeliads, and is very similar to restinga vegetation (Dias and Brescovit 2003), found in coastal region which is a typical enviroment for *Pachistopelma rufonigrum* in Rio Grande do Norte state (Santos et al. 2004). Bromeliad phytotelma is a source of water, food and retreat for a variety of animal species (Frank and Lounibos 2009), and in restinga regions they are a key resource for the local fauna (Santos et al. 2002; 2003a, b). I failed to find *Pachistopelma bromelicola* sp. n. in parts of Parque Nacional de Itabaiana covered with Brazilian Atlantic rainforest. In RPPN Sapiranga, *Pachistopelma bromelicola* sp. n. was found in restinga area, inside *Hohenbergia stellata* bromeliads. Some of these bromeliads were very close to a house and were used in garden decoration. Inside two bromeliads we found an eggsac, protected by a retreat, in October 2007. In a region covered with Brazilian Atlantic rainforest, in RPPN Jequitiba, they were found close to a house located relatively far from forest shade. Other specimens were colected inside bromeliads in a caatinga (a xeric shrubland and thorn forest) region in Jeremoabo (R. A. Sanfilippo pers. comm.). Therefore, *Pachistopelma bromelicola* sp. n. is distributed over contrasting environments, from rainforest to xeric caatinga and restinga. An element in common among these populations is the obligatory bromelicolous habits.

#### Color pattern ontogeny.

The color pattern is similar to *Pachistopelma rufonigrum*, mainly in early instars. However, the lateral black stripes of abdomen dorsum are almost always connecting with the longitudinal central stripe (Figs 52–53) whereas in *Pachistopelma rufonigrum* they normally do not connect (Figs 41–43). Larger individuals have dark legs and dark-brown carapace, and abdominal pattern is conspicuous (Fig. 54). In subadults, abdomen is very dark, lacks a pattern, or is inconspicuous (Fig. 55). Adult female is almost completely black, except for some brown setae over carapace (Fig. 56). Adult male is also completely black, but with many long whitish setae on legs, carapace and chelicerae. Abdomen is black with long red setae (Fig. 57).

### Iridopelma

Pocock, 1901

http://species-id.net/wiki/Iridopelma

Figs 81 [Fig F17] [Fig F18] [Fig F19] [Fig F20] [Fig F21] [Fig F22] [Fig F23] [Fig F24] [Fig F25] [Fig F26] [Fig F27] [Fig F28] [Fig F29] [Fig F30] [Fig F31] [Fig F32] –169

Iridopelma Pocock 1901:549; Raven 1985:119; Smith 1993:15, f. 1–10; Platnick 2012.
Avicularia : Simon 1903:960 (in part: *Avicularia hirsuta*); Schmidt 1986:61, f. 109–110.
Typhochlaena : Mello-Leitão 1923:332 (in part: *Typhochlaena pococki*, superfluous new name).

#### Type species.

*Iridopelma hirsutum* Pocock, 1901 by original designation.

#### Diagnosis.

Males of *Iridopelma* species differ from those of other aviculariines in the presence of tibial spurs on both leg I (Fig. 85) and II (Fig. 86). Females differ from those of all other aviculariines, except *Avicularia* and *Typhochlaena*, in that the anterior eye row is strongly procurved. *Iridoplema* further differs from *Typhochlaena* in that the most distal PLS segment is digitiform; from *Avicularia* in possessing spermatheca lacking an accentuated curvature medially.

**Figures 81–88.**
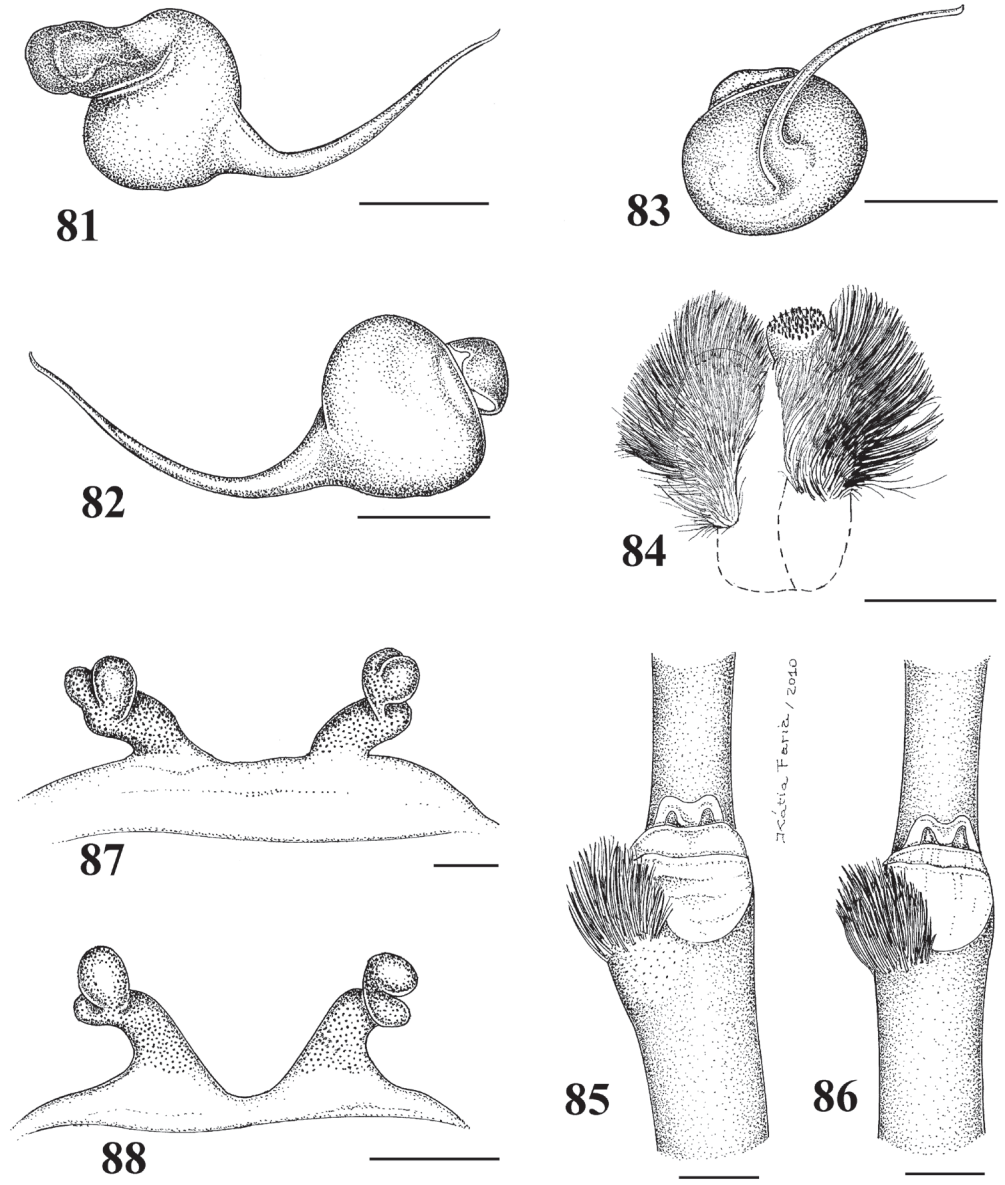
*Iridopelma hirsutum* Pocock, 1901 **81–86** male (MZSP 36884) **81–83** left palpal bulb **81** prolateral **82** retrolateral, **83** frontal **84** cymbium showing protuberance **85** tibial spur of left leg I **86** tibial spur of left leg II **87–88** female spermathecae **87** (MNRJ 06251) **88** (MNRJ 06249). Scale bar = 1mm.

#### Description.

Carapace longer than wide, cephalic region moderately raised. Cephalic and thoracic striae conspicuous. Fovea straight, shallow or deep. Chelicerae without rastellum. Eye tubercle raised (Fig. 91), wider than long. Clypeus narrow (Fig. 92). Anterior eye row procurved (Fig. 92). Labium wider than long, with 40–200 cuspules spaced by one diameter from each other on the anterior third center. Maxillary lyra absent. Maxilla subrectangular, anterior lobe distinctly produced in to conical process, inner angle bearing (75–150) cuspules. Sternum wider than long (Fig. 93). Posterior angle not separating coxae IV. Three pairs of sigillae, all rounded, less than one diameter from margin. Leg formula: I=IV II III (all females, males of *Iridopelma vanini* sp. n., *Iridopelma katiae* sp. n. and*Iridopelma zorodes*) or I IV II III (males of *Iridopelma hirsutum* and *Iridopelma oliveirai* sp. n.). Clavate trichobothria on the distal 2/3 of tarsi I–IV. STC of males and femalewithout teeth. Tarsi I–III fully scopulated, IV divided by a band of setae or scopula integral. Scopulae of tarsi and metatarsi I–II extended very laterally giving them a spatulate appearance. Femur IV without retrolateral scopula. Legs lacking spines. Posterior lateral spinneret distally elongating, digitiform (Fig. 94). Stridulatory setae absent. Cymbium with two subequal lobes, the prolateral one triangular in shape. Male spur on tibia I (Fig. 85) and II (Fig. 86). Male metatarsus I straight. Male palpal bulb globose narrowing abruptly forming a long slender embolus, 1.8 times (*Iridopelma oliveirai* sp. n.) (Figs 156–158), 2.4–2.7 times (*Iridopelma hirsutum*, *Iridopelma zorodes*) (Figs 81–83, 107–109) or 3.3 times (*Iridopelma vanini* sp. n., *Iridopelma katiae* sp. n**.**) (Figs 127–129, 134–136) longer than tegulum length, with curvature of roughly 60° (*Iridopelma hirsutum*) (Fig. 83), 90° (*Iridopelma katiae* sp. n.) (Fig. 136), 105° (*Iridopelma oliveirai* sp. n.) (Fig. 158), 115° (*Iridopelma zorodes*) (Fig. 109) or 135° (*Iridopelma vanini* sp. n.) (Fig. 129) to retrolateral side; keels absent, tegulum without prolateral depression. Paired straight spermathecae ending in two or three lobes (*Iridopelma hirsutum*, *Iridopelma oliveirai* sp. n., *Iridopelma vanini* sp. n., *Iridopelma marcoi* sp. n., *Iridopelma katiae* sp. n.) (Figs 87–88, 161, 132, 166, 139), or a single lobe (*Iridopelma zorodes*) (Fig. 112); with a single apical fold (*Iridopelma hirsutum*, *Iridopelma oliveirai* sp. n.) (Figs 87–88, 161), double folded (*Iridopelma marcoi* sp. n., *Iridopelma vanini* sp. n.) (Figs 166, 132), or without folds (*Iridopelma zorodes*, *Iridopelma katiae* sp. n.) (Figs 112, 139). Cymbium with well developed spiniform process (Fig. 84). Type II urticating hair on abdomen dorsum of males and females (most species), or absent in adult females (*Iridopelma marcoi* sp. n.). Color pattern ontogeny present.

Male *Iridopelma marcoi* sp. n. are currently unknown.

**Figures 89–94. F17:**
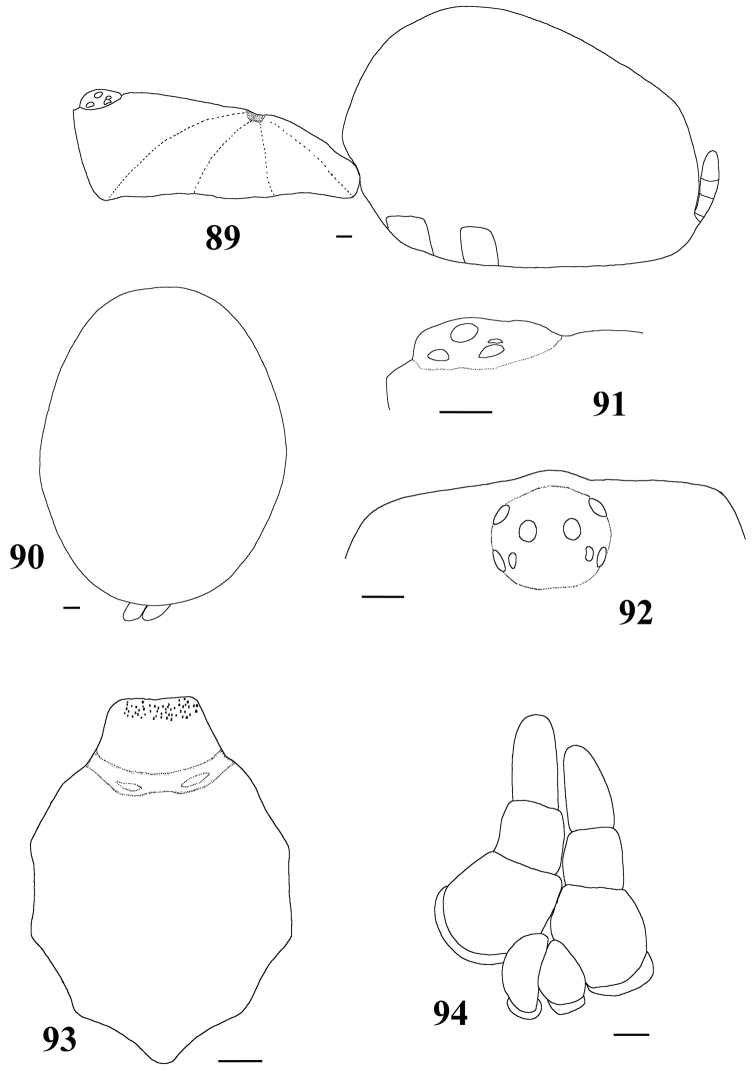
*Iridopelma hirsutum* Pocock, 1901, female (MNRJ 06249) **89** carapace and abdomen, lateral **90** abdomen, dorsal **91–92** eye tubercle **91** lateral **92** dorsal **93** labium and sternum **94** spinnerets, ventral. Scale bar = 1mm.

**Figures 95–100. F18:**
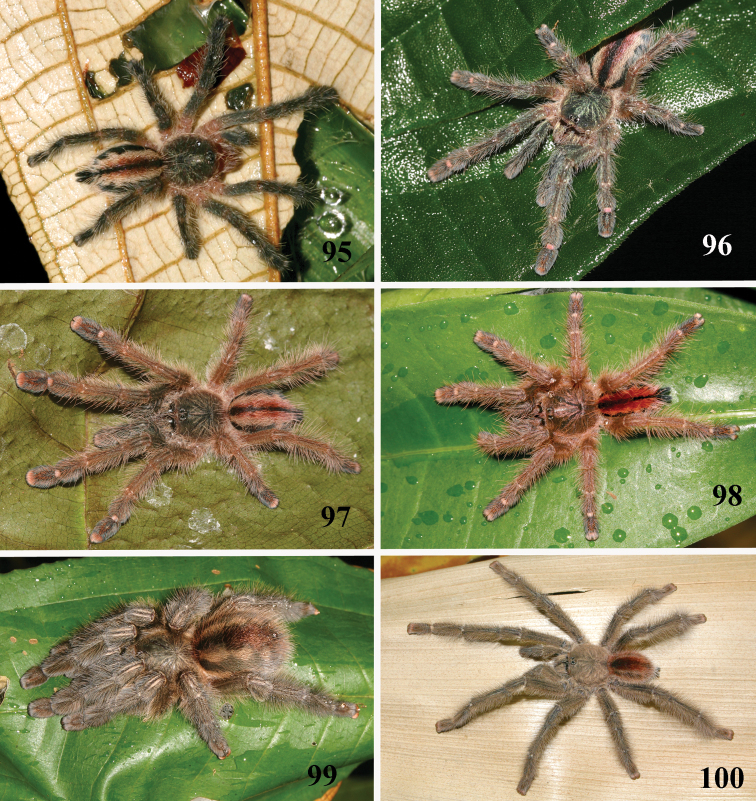
*Iridopelma hirsutum* Pocock, 1901, habitus **95–98** immatures in progression **99** female **100** male, all from Reserva Biológica de Saltinho, Rio Formoso, state of Pernambuco, except female, from E. E. Murici, Murici, state of Alagoas. Photos: R. Bertani.

**Figures 101–106. F19:**
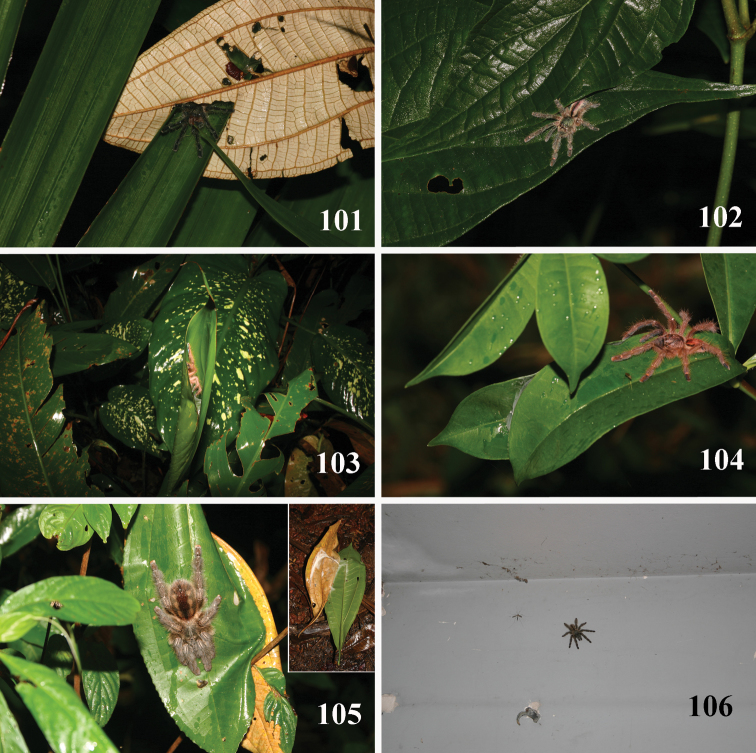
*Iridopelma hirsutum* Pocock, 1901, microhabitats **101** immature in retreat made with palm/Melastomatacea leaves **102, 104** immature in retreat made of two large unidentified leaves **103** immature in a retreat made of a Heliconiacea leaf **105** adult female in a retreat made of two leaves of “Guapeba” (Sapotaceae) **106** female on an abandoned house wall **101–104** Saltinho, state of Pernambuco, **105–106** E. E. Murici, Murici, state of Alagoas. Photos: R. Bertani.

**Figures 107–112. F20:**
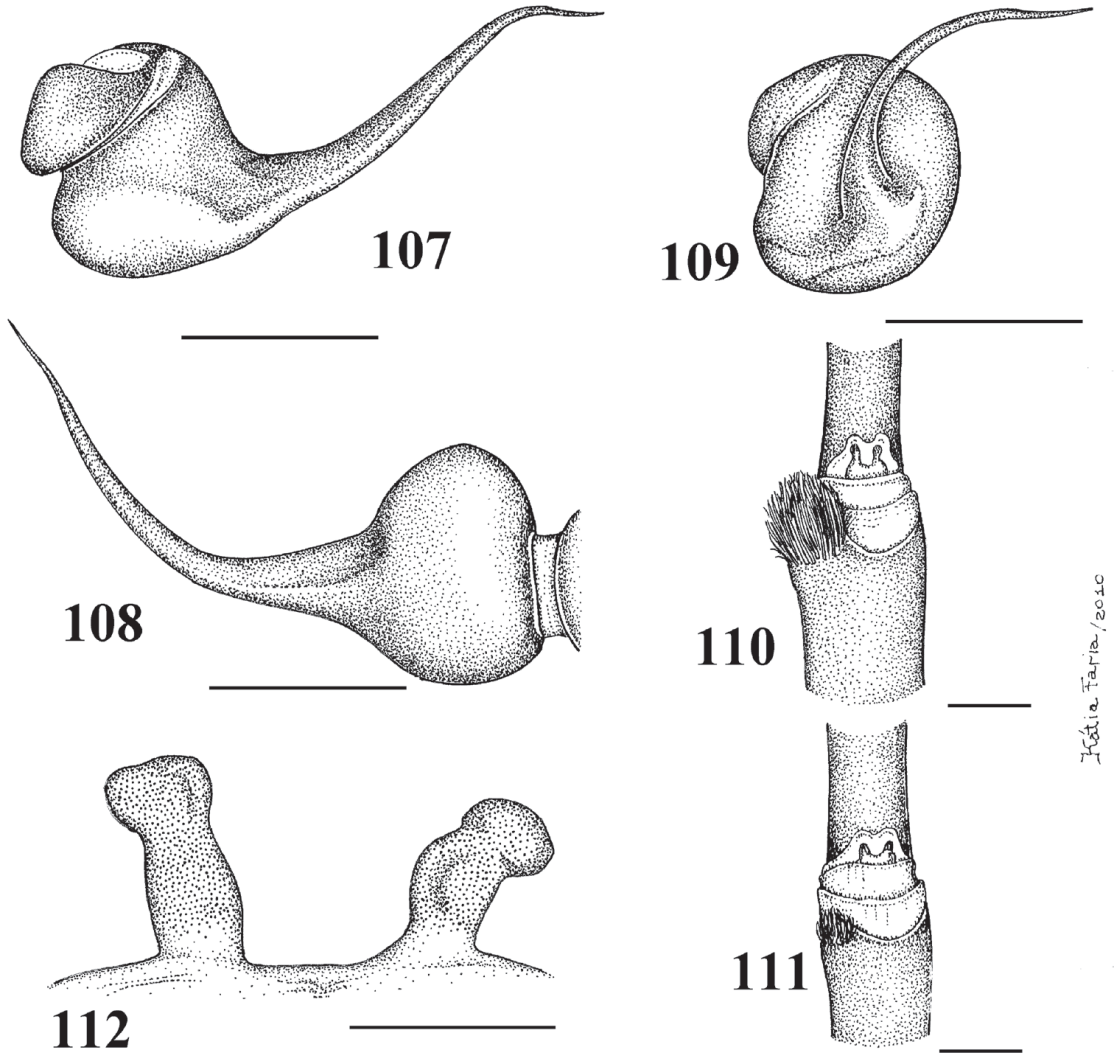
*Iridopelma zorodes* (Mello-Leitão, 1926) **107–111** male (MNRJ 06254) **107–109** left palpal bulb **107** prolateral **108** retrolateral **109** frontal **110** tibial spur of left leg I **111** tibial spur of left leg II **112** female (IBSP 11760) spermathecae. Scale bar = 1mm.

**Figures 113–118. F21:**
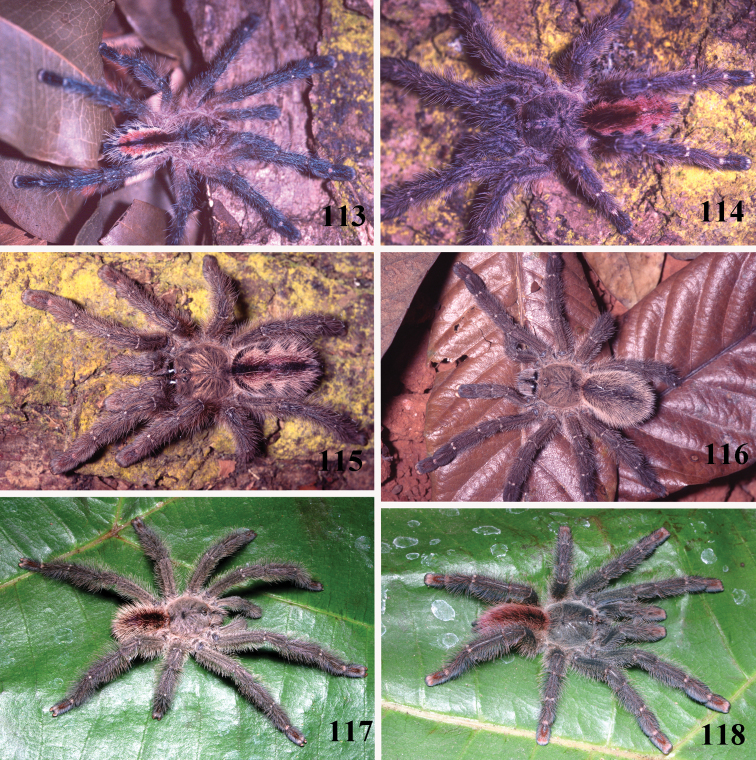
*Iridopelma zorodes* (Mello-Leitão, 1926), habitus **113–115** immatures in progression **116** female **117** male **118** female **113–116** immatures and female from Itabaiana National Park, Areia Branca, state of Sergipe **117** male from RPPN Camurujipe, São João da Mata, state of Bahia **118** female from Elísio Medrado, RPPN Jequitiba, state of Bahia (MNRJ 06253). Photos: R. Bertani.

**Figures 119–126. F22:**
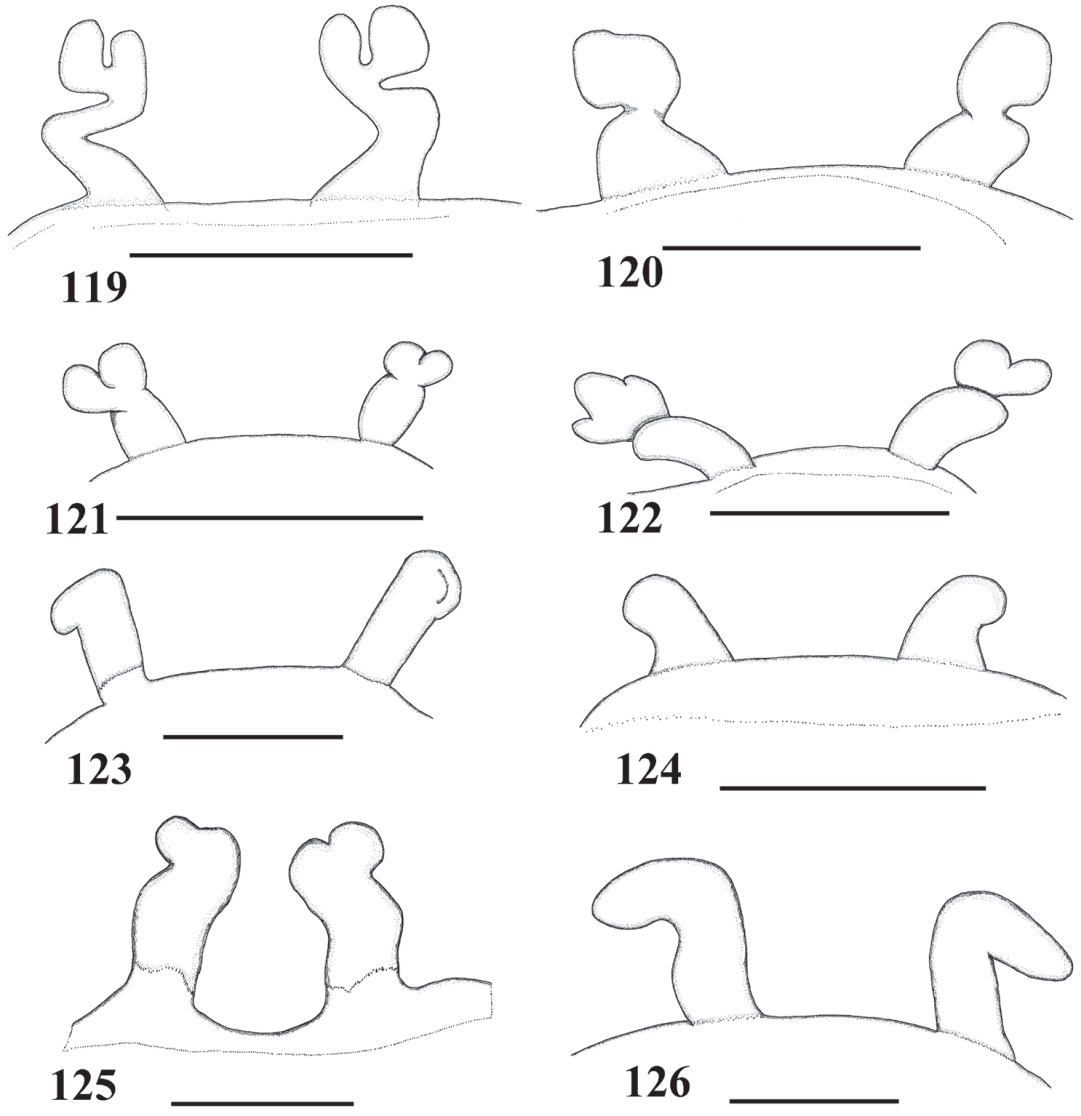
Variation in spermathecae **119–122** *Iridopelma hirsutum* Pocock, 1901 **119** João Pessoa, state of Paraiba (IBSP 8076) **120** Sucupira, state of Pernambuco (IBSP 3986A) **121** Cabedelo, Ilha da Restinga, state of Paraiba (IBSP 8082) **122** Olinda, state of Pernambuco (IBSP 8080) **123–126** *Iridopelma zorodes* (Mello-Leitão, 1926) **123** Santo Amaro das Brotas, state of Sergipe (MZSP 10847) **124, 126** Acajutiba, state of Bahia (MZSP 32160–698, MZSP 32181–800) **125** Elísio Medrado, RPPN Jequitibá, state of Bahia (MNRJ 06253). Scale bar = 1mm.

**Figures 127–132. F23:**
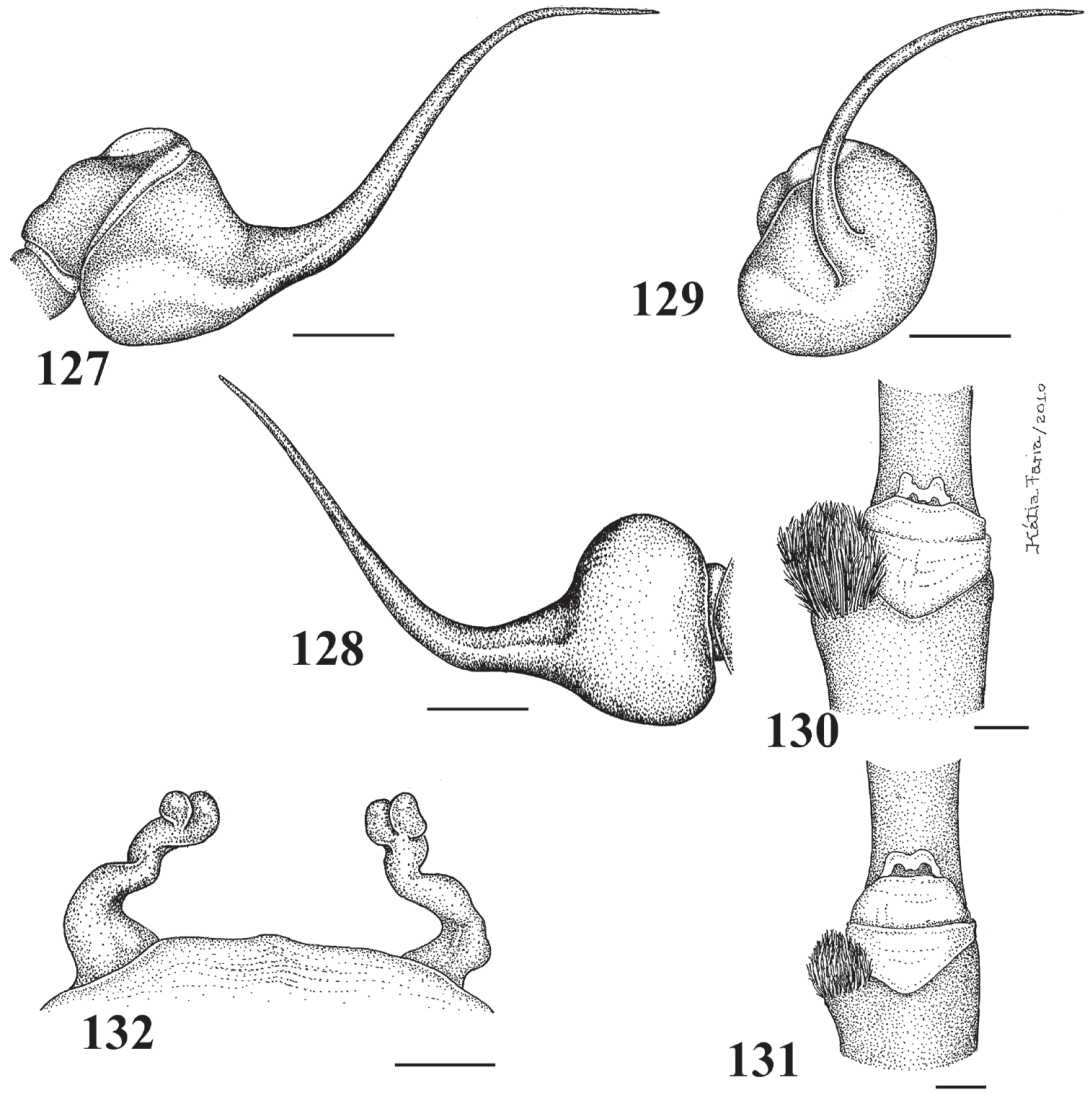
*Iridopelma vanini* sp. n. **127–131** male **127–129** left palpal bulb (IBSP 11328) **127** prolateral **128** retrolateral **129** frontal **130** tibial spur of left leg I **131** tibial spur of left leg II **132** holotype female (IBSP Ref. 74.595) spermathecae. Scale bar = 1mm.

#### Species included.

*Iridopelma hirsutum* Pocock, 1901 (Figs 81–106, 119–122, 169), *Iridopelma zorodes* (Mello-Leitão, 1926) (Figs 107–118, 123–126, 169), *Iridopelma vanini* sp. n. (Figs 127–133, 150–152, 169), *Iridopelma katiae* sp. n. (Figs 134–149, 153–155, 169), *Iridopelma oliveirai* sp. n. (Figs 156–165, 169), *Iridopelma marcoi* sp. n. (Figs 166–169).

#### Distribution and habitat.

Brazil: Northeastern, from Reconcave region in state of Bahia northwards. Few records for states of Para and Tocantins (Fig. 169). Specimens of *Iridopelma* spp. are mostly found in Brazilian Atlantic rainforest (Figs 101–105). Some species occur in drier, open environments such as “cerrado” (Fig. 168), “caatinga” and “campo rupestre” (Figs 145–146). A single record is known from the Amazon.

#### Natural history.

*Iridopelma hirsutum* and *Iridopelma zorodes* build retreats connecting leaves with silk threads (Figs 101–105). *Iridopelma katiae* sp. n. make retreats inside bromelids (Figs 147–149). Habitat preference of other *Iridopelma* species is in need of confirmation.

**Figure 133. F24:**
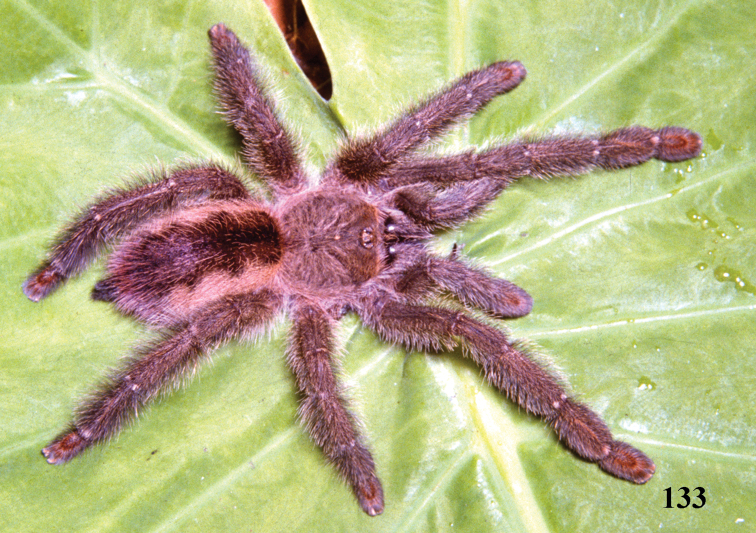
*Iridopelma vanini* sp. n., holotype female, habitus, from Parnamirim, state of Piaui. Photo: R. Bertani.

**Figure F25:**
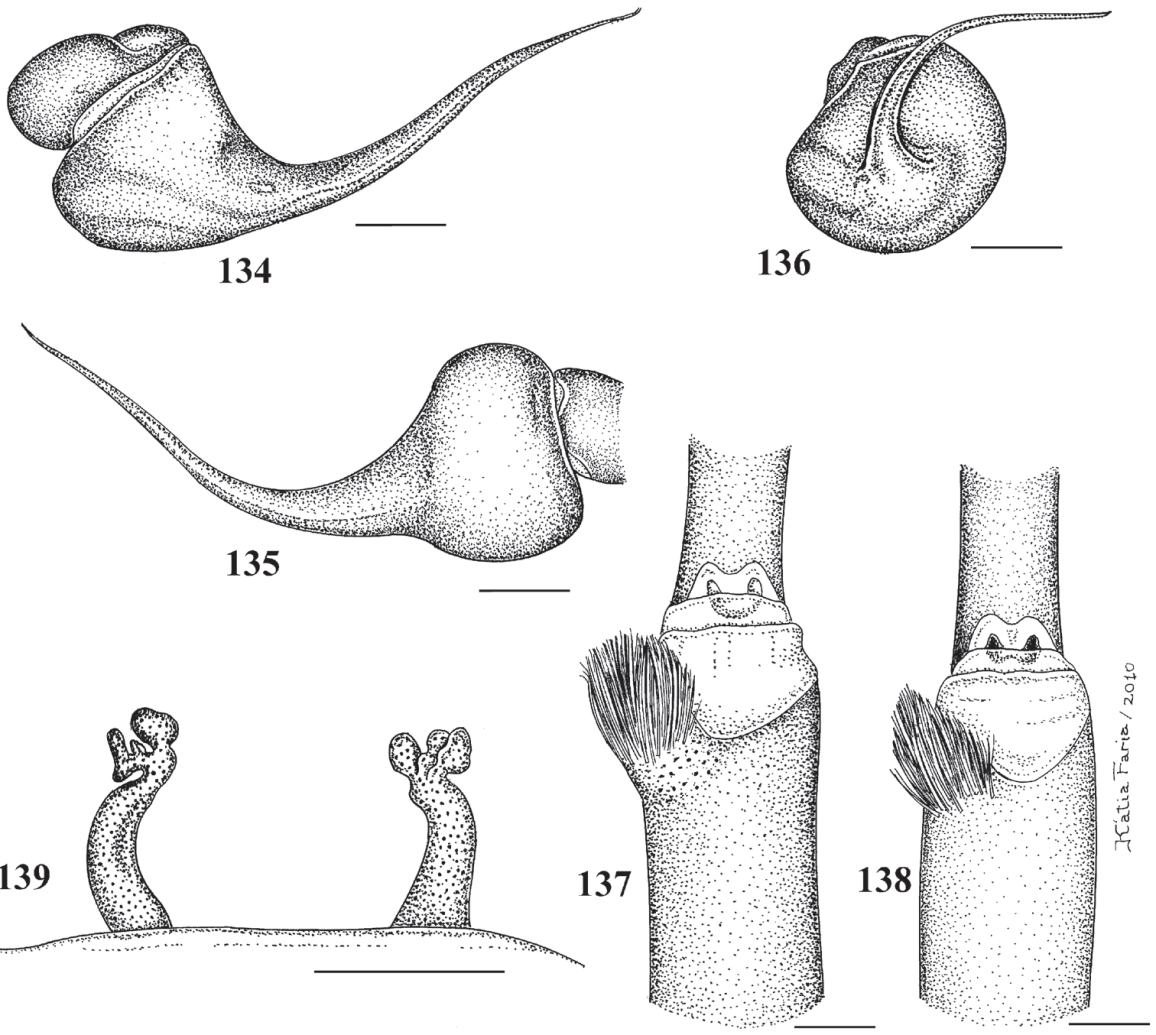
**Figures 134–139.** *Iridopelma katiae* sp. n. **134–138** paratype male (MZSP 36888) **134–136** left palpal bulb **134** prolateral **135** retrolateral **136** frontal **137** tibial spur of left leg I **138** tibial spur of left leg II **139** holotype female (MZSP 36887) spermathecae. Scale bar = 1mm.

**Figures 140–143. F26:**
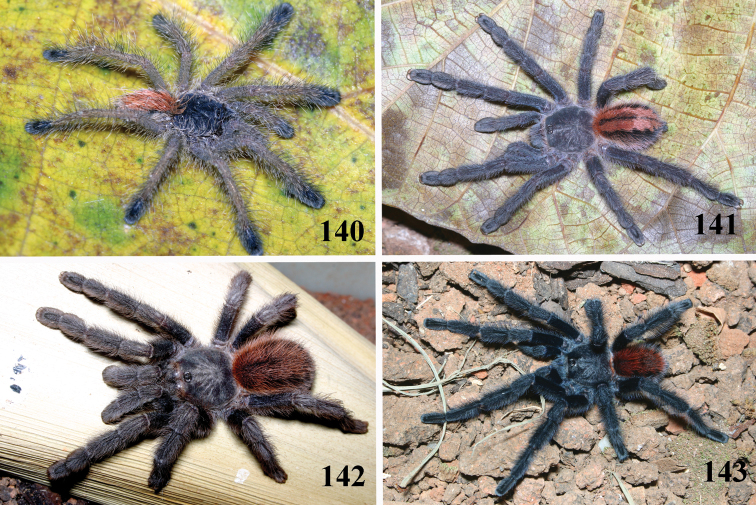
*Iridopelma katiae* sp. n., habitus **140** small immature **141** large immature **142** female **143** male, all from Chapada Diamantina National Park, Mucuge, state of Bahia. Photos: R. Bertani.

**Figures 144–149. F27:**
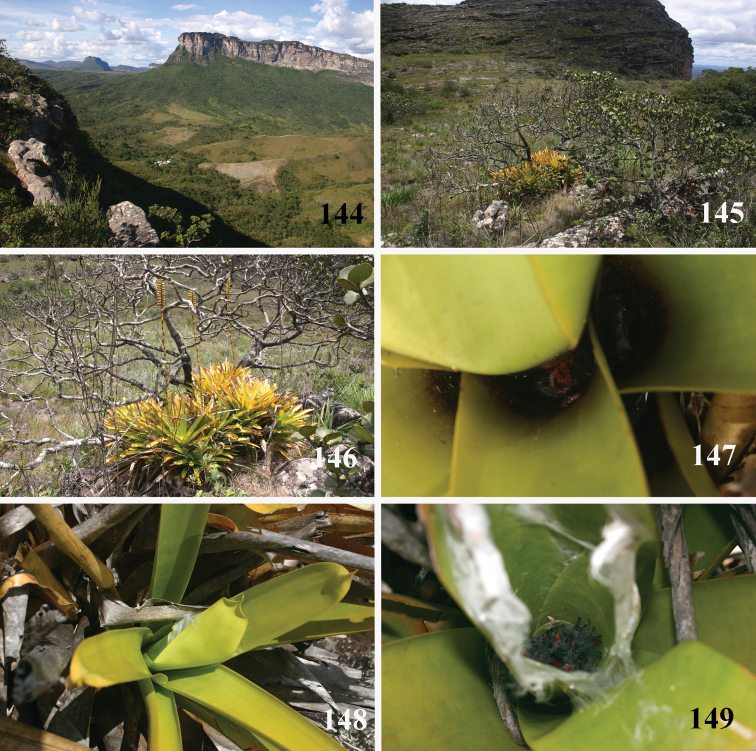
*Iridopelma katiae* sp. n. habitats **144** general view of Chapada Diamantina showing “Chapada” formation **145** detail of top of “Chapada” formation showing “campo rupestre” area and a bromeliad (*Vriesea atra*)“island” **146** detail of the bromeliad “island” **147** *Iridopelma katiae* sp. n. inside *Vriesea atra* leaves **148** *Vriesea atra* leaf folded and linked with silk threads making retreat **149** opened retreat, showing young. Photos: R. Bertani.

**Figures 150–155. F28:**
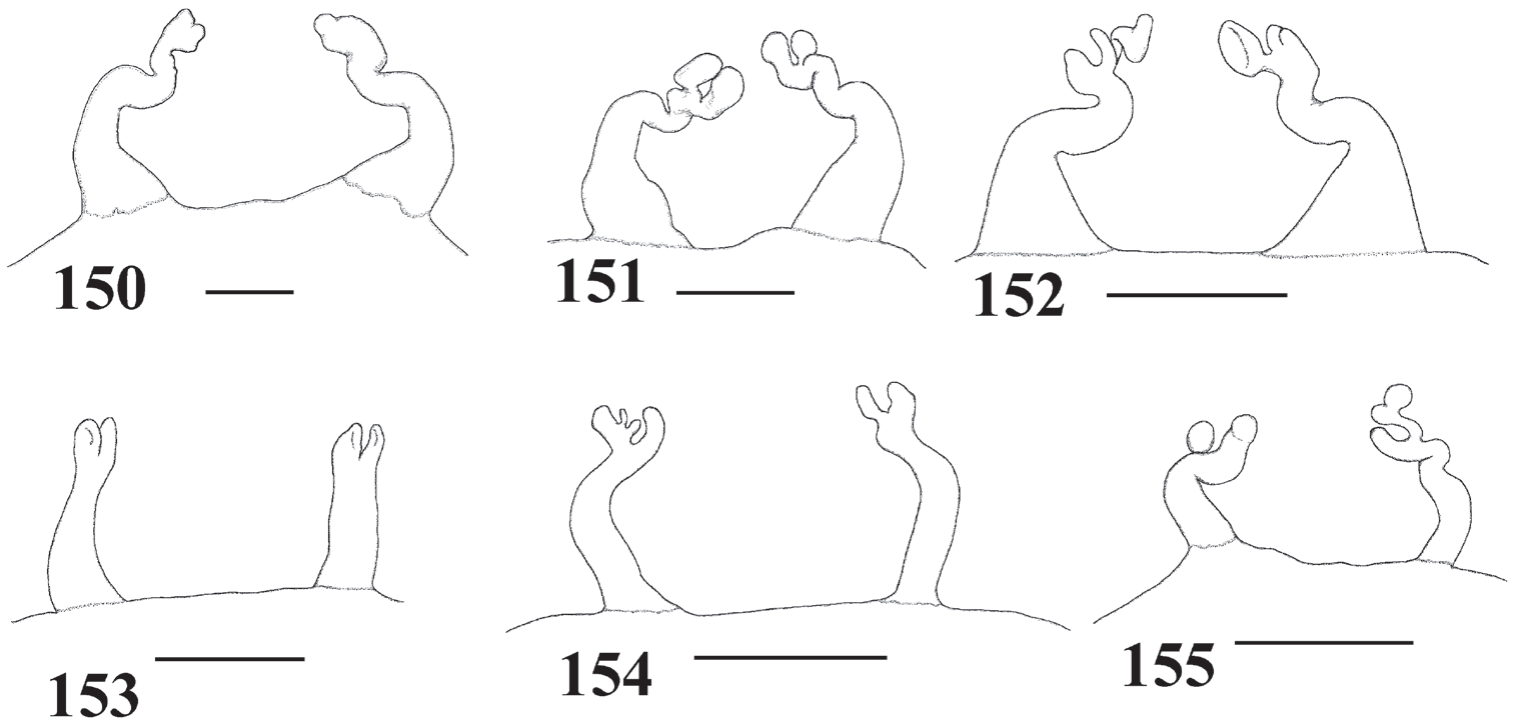
Variation in spermathecae **150–152** *Iridopelma vanini* sp. n. **150–151** Barreirinhas, state of Maranhão (IBSP 11343, IBSP 11330) **152** Colinas de Tocantins, state of Tocantins (IBSP 3918) **153–155** *Iridopelma katiae* sp. n. **153** Rio de Contas, state of Bahia (IBSP 10317) **154** Mucugê, Parque Nacional da Chapada Diamantina, state of Bahia (MZSP 36890) **155** Ibicoara, state of Bahia (MZSP 22762). Scale bar = 1mm.

**Figures 156–161. F29:**
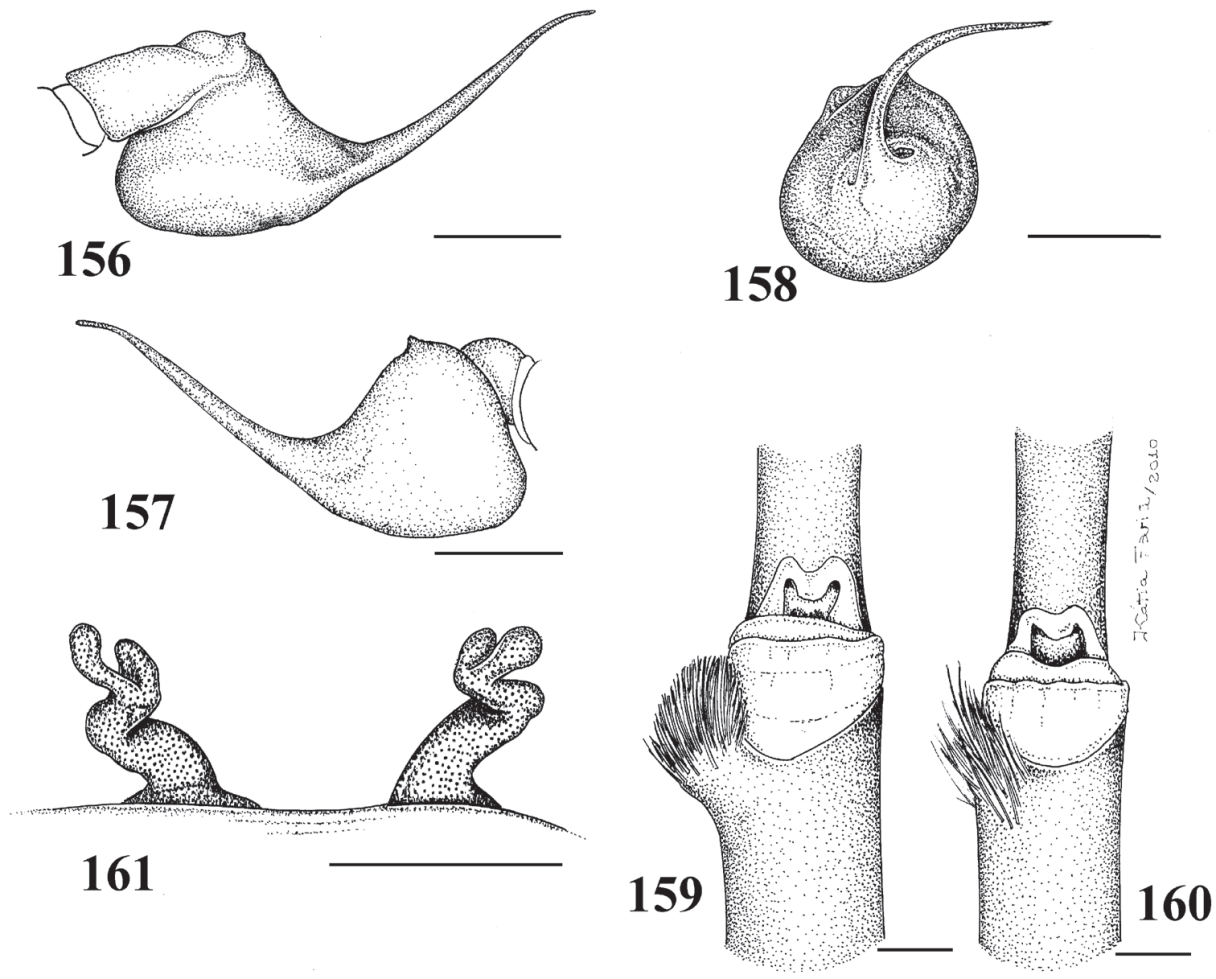
*Iridopelma oliveirai* sp. n. **156–160** male (IBSP 10100) **156–158** left palpal bulb **156** prolateral **157** retrolateral **158** frontal **159** tibial spur of left leg I **160** tibial spur of left leg II **161** female (IBSP 8714), spermathecae. Scale bar = 1mm.

**Figures 162–165. F30:**
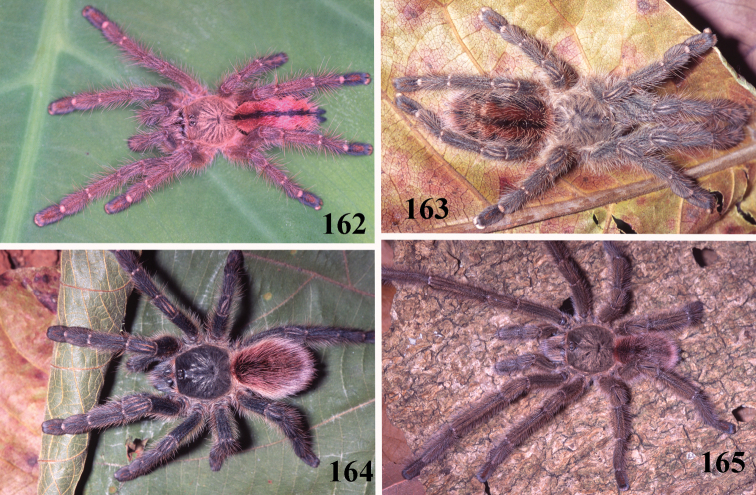
*Iridopelma oliveirai* sp. n., habitus **162–163** immatures in progression **164** paratype female (IBSP 8714) **165** holotype male (IBSP 10100), all from Central, state of Bahia. Photos: R. Bertani.

**Figure 166. F31:**
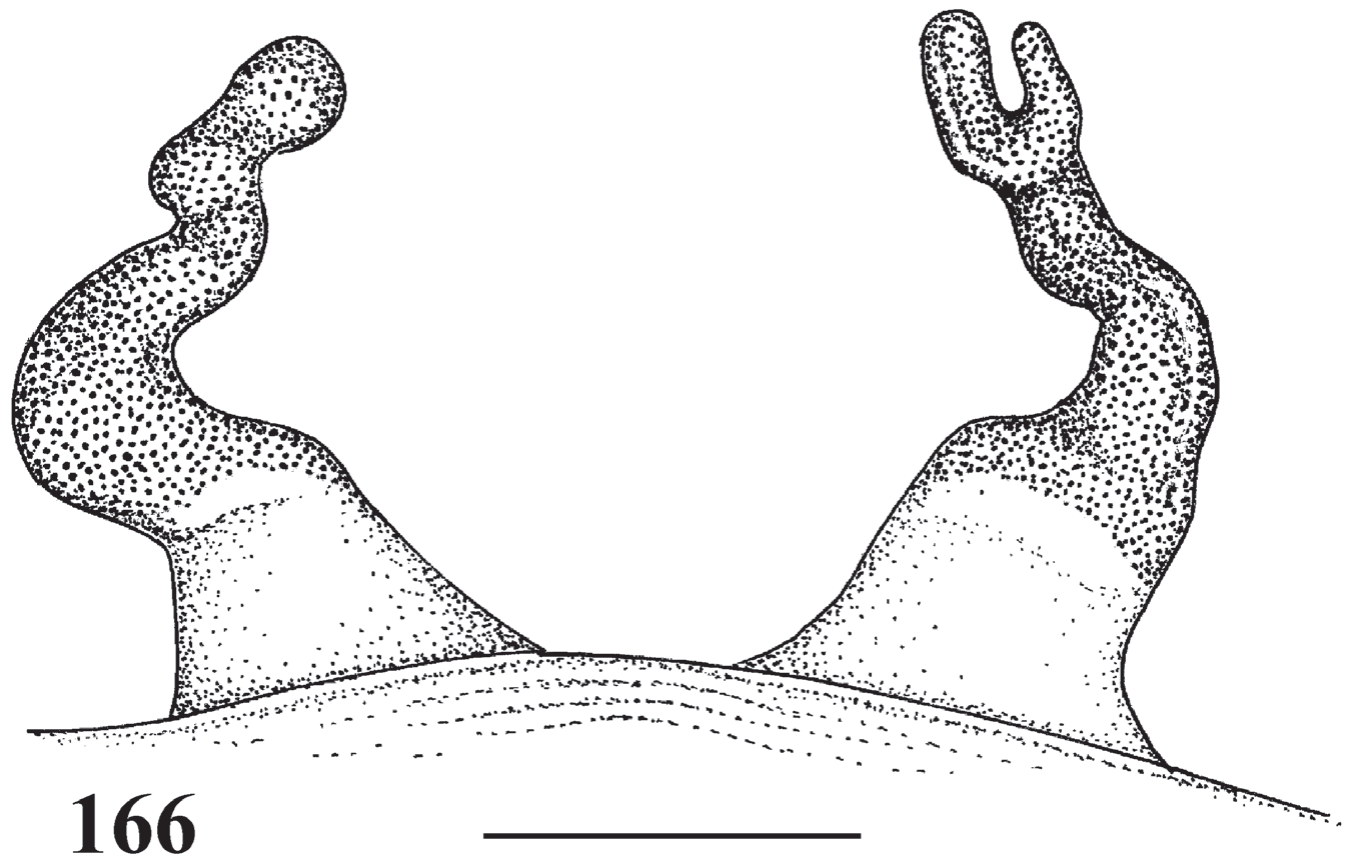
*Iridopelma marcoi* sp. n. holotype female (IBSP 36891), spermathecae. Scale bar = 1 mm.

**Figures 167–168. F32:**
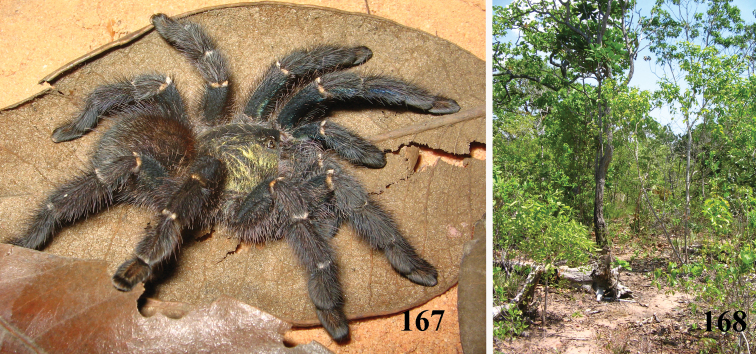
*Iridopelma marcoi* sp. n. **167** holotype female (IBSP 36891), habitus **168** habitat, São Desidério, state of Bahia. Photos: Marco A. Freitas.

**Figure 169.**
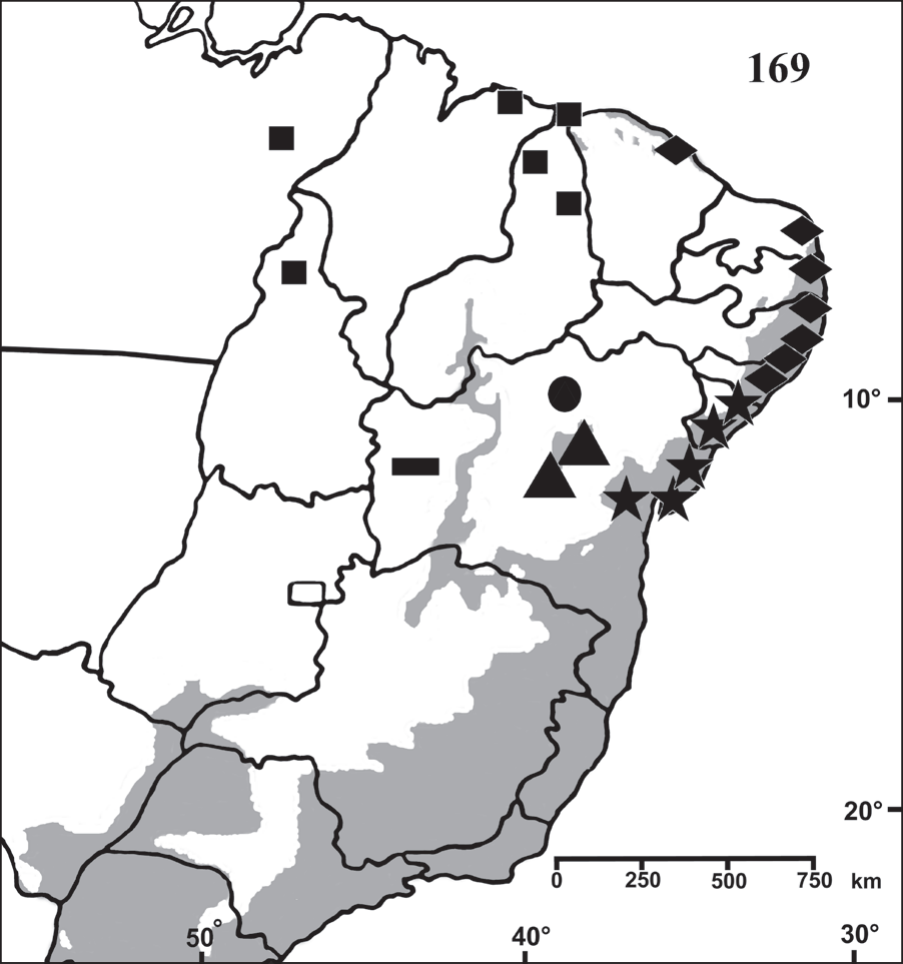
Map showing records of *Iridopelma* species in Northestern, Central western and Northern Brazil. Diamond = *Iridopelma hirsutum* Pocock, 1901, star = *Iridopelma zorodes* (Mello-Leitão, 1926), square = *Iridopelma vanini* sp. n., retangle = *Iridopelma marcoi* sp. n., triangle = *Iridopelma katiae* sp. n., circle = *Typhochlaena oliveirai* sp. n. The gray area represents the approximate original distribution of Brazilian Atlantic rainforest. White area represents open environments (cerrado and caatinga).

#### Color pattern ontogeny.

*Iridopelma hirsutum*, *Iridopelma zorodes* and *Iridopelma katiae* sp. n. color pattern changes dramatically from juvenile to adult stages. Data is insufficient for *Iridopelma vanini* sp. n., *Iridopelma oliveirai* sp. n. and *Iridopelma marcoi* sp. n.

#### Key to species of *Iridopelma*

(Male *Iridopelma marcoi* sp. n. is unknown)

**Table:** 

1	Male	2
–	Female	6
2	Leg I longer than leg IV (leg IV/I length 0.84 or less)	3
–	Leg I and IV roughly same length (leg IV/I length 0.93 or more)	4
3	Leg I much longer than leg IV (leg IV/I length 0.73), pattern lacking on abdomen dorsum (Fig. 165); embolus short (Figs 156–158)	*Iridopelma oliveirai* sp. n.
–	Leg I not so longer than leg IV (leg IV/I length 0.84), pattern on abdomen dorsum present (Fig. 100); embolus long (Figs 81–83)	*Iridopelma hirsutum*
4	General coloration black with reddish pattern on abdomen dorsum (Fig. 143)	*Iridopelma katiae* sp. n.
–	General coloration brown or light-brown, lacking pattern on abdomen dorsum	5
5	Large spiders, embolus very long (Figs 127–129)	*Iridopelma vanini* sp. n.
–	Small to medium-sized spiders, embolus shorter (Figs 107–109)	*Iridopelma zorodes*.
6	Spermathecae with a single lobe and lacking folds (Figs 112, 123–126)	*Iridopelma zorodes*
–	Spermathecae with two or more lobes	7
7	Spermathecae slender from base to tip, lacking folds (Fig. 139, 153–155); general coloration black with reddish pattern on abdomen dorsum (Fig. 142)	*Iridopelma katiae* sp. n.
–	Spermathecae tapering from base to tip, with at least one fold; general coloration brown or metallic green	8
8	Spermathecae very long, double folded (Figs 132, 150–152)	*Iridopelma vanini* sp. n.
–	Spermathecae not too long, with single fold (Figs 87–88)	9
9	Pattern on abdomen dorsum present, with longitudinal reddish central stripe (Fig. 99)	*Iridopelma hirsutum*.
–	Abdomen dorsally lacking pattern (Fig. 164)	10
10	Carapace metallic green (Fig. 167); urticating hair lacking on abdomen dorsum	*Iridopelma marcoi* sp. n.
–	Carapace brownish to grayish (Fig. 164); urticating hair type II present on abdomen dorsum	*Iridopelma oliveirai* sp. n.

### Iridopelma hirsutum

Pocock, 1901

http://species-id.net/wiki/Iridopelma_hirsutum

Figs 81 [Fig F17] [Fig F18] –106 119–122 169

Iridopelma hirsutum Pocock 1901:550; Smith 1993:15, f. 1–10; Peters 2000:34, f. 73–74; 2003:209, f. 843–844; Platnick 2012.
Avicularia hirsuta : Simon 1903:960; Petrunkevitch 1911:50; Roewer 1942:254; Bücherl 1957:404, f. 93–93a; Schmidt 1986:61, f. 109–110; 1993:102, f. 326–327; 2003:200, f. 574–575.
Typhochlaena pococki Mello-Leitão 1923:333 (superfluous new name for *Iridopelma hirsutum* Pocock, 1901).
Avicularia palmicola Mello-Leitão 1945:170; Bücherl et al. 1971:126, f. 44. **syn. n.**

#### Diagnosis.

Males resemble those of *Iridopelma oliveirai* sp. n. by leg I longer than leg IV (leg IV/I length = 0.84, SD = 0.05). Can be further distinguished by narrow embolus ending in a curved tip (Figs 81–83) and by a pattern present on the abdomen dorsally (Fig. 100). Female resembles *Iridopelma oliveirai* sp. n. by diverging spermathecae twisted in apical portions (Figs 87–88). It differs by brown carapace and legs, and an obvious abdominal pattern (Fig. 99).

#### Types.

Lectotype female (herein designated) a dissected adult female and 4 paralectotypes, 1 male, 3 immatures fom Pernambuco, Brazil, Royal Soc, H. N. Ridley (BMNH 1889-8-9-19) examined. Remarks: Smith (1993) considered the male from Igarassu, collected by Ramage (BMNH 1888-47) to be the species type. However, he overlooked that Pocock (1901) explicitly indicated in the original description the types as those from Pernambuco. Furthermore, Pocock (1901) described both female and male, whereas Ramage’s material consists only of a single male.

#### Additional material examined.

BRAZIL: *Ceará*: Fortaleza [3°43'S, 38°32'W], 1 male, H. A. Braga, 10 April 1947 (IBSP 3505); 1 male, M. P. Paiva, 16 November 1960 (IBSP 3555); 2 males, 1 female, D. Rocha (IBSP 3362); 1 immature, same collector (IBSP 3361); *Rio Grande do Norte*: Parnamirim [5°54'S, 35°15W], 1 male, C. A. Almeida, 9 April 1973 (IBSP 2741); 1 male, F. Kawata, November 2007 (IBSP 14685); *Paraíba*: 1 male, A. R. Roman, October 1980 (IBSP 4658); 1 immature, M. Ihering (MNRJ 42344); Cabedelo, Ilha da Restinga [7°00'S, 34°50'W], 1 female, P. F. L. Duarte, 12 November 1978 (IBSP 8082); João Pessoa [7°08'S, 34°51W], 2 males, P. F. L. Duarte (IBSP 9758); Campus da U.F.P.B., 1 male (IBSP 8077), 1 female, Robson and Paula, 29 September to 3 October (IBSP8079), 1 female, B. M. Grisi, 23 August 1977 (IBSP 8076), 1 male, P. F. L. Duarte, 30 November 1979 (IBSP 8078), 1 female, Carrocinha, 17 September 1976 (MNRJ 13778), 1 male, P. F. L. Duarte, 1976 (MNRJ 13745), 1 immature, P. F. L. Duarte, 5 May 1977 (MNRJ 13549); *Pernambuco*: 1 male, P. F. L. Duarte (IBSP 9757); Camocim de São Félix [8°21'S, 35°45'W], 1 immatue, Vera Lúcia, 25 November 1969 (IBSP 8081); Jaboatão dos Guararapes, Sucupira [8°06'S, 34°58'W], 1 immature, J. M. Arcanjo, 21 May 1969, on bromeliads (IBSP 3986A); Igarassu [7°50'S, 34°54'W], 1 male, G. A. Ramage (BMNH 1888-47); Olinda [8°00'S, 34°51'W], 2 females, 8 immatures, A. T. Coelho, 1963, on bushes (IBSP 8080); Recife [8°03'S, 34°52'W], 1 female, M. F. Santos, May 1985 (IBSP 2741); Rio Formoso, Reserva Biológica de Saltinho (8°43'5.99"S, 35°10'7.08"W), 1 male, R. Bertani, D. R. M. Ortega and R. H. Nagahama, 9 August 2006, at night, with retreat made with silk and two leaves, 1 m above the ground (MZSP 36884); 1 female, same colectors, 8 August 2006 (MZSP 36885); 1 female, same collectors, 10 August 2006, at night, on a leaf 0.5 m above the ground (MZSP 36886); 1 immature, same collectors, 9 August 2006, at night in palm tree, inside retreat made with silk and two leaves, 1 m above the ground (MZSP 36887), 2 immatures, 12 May 1969 (MNRJ 13721); *Alagoas*: Coruripe, Barragem da Fazenda Capiatã I, Usina Coruripe [10°08'S, 36°10'W], 1 male, E. C. Santos and R. C. T. Aquino (ex MUFAL 269), October 2004 (IBSP 12583), Murici, Estação Ecológica de Murici, Ufal (9°15'1.86"S, 35°51'0.17"W), 200 m a.s.l., 1 female, R. Bertani, D. R. M. Ortega and R. H. Nagahama, 17 August 2006, at night, inside reatreat made with silk and two leaves, 1 m above the ground (MNRJ 06249); 1 immature, same colectors, 12 August 2006, at night, in a web built between two *Heliconia* leaves (MNRJ 06250); (9°14'1.73"S, 35°50'1.61"W), 575 m a.s.l., 1 female, same collectors, 16 August 2006, at night in a wall of an abandoned house (MNRJ 06251); 1 female, same data (MNRJ 06252); without locality (Cidade Universitaria), 1 male, M. N. Marques, 11 May 1980 (MNRJ 13782).

#### Redescription.

Female (MNRJ 06249) from Murici, state of Alagoas, Brazil. Carapace 15.0 long, 14.4 wide, chelicerae 9.0. Legs (femur, patella, tibia, metatarsus, tarsus, total): I: 13.4, 7.6, 10.4, 9.6, 5.3, 46.3. II: 12.3, 7.0, 8.8, 9.3, 4.9, 42.3. III: 10.4, 6.0, 8.0, 8.6, 4.6, 37.6. IV: 12.6, 6.2, 10.5, 10.9, 4.7, 44.9. Palp: 9.4, 5.2, 6.2, –, 6.2, 27.0. Mid-widths (lateral): femora I –IV = 2.8, 2.8, 3.1, 2.8, palp = 2.2; patellae I–IV = 2.9, 2.8, 2.7, 2.7, palp = 2.4; tibiae I–IV = 2.7, 2.5, 2.5, 2.6, palp = 2.3; metatarsi I–IV = 2.2, 2.2, 1.8, 1.6; tarsi I–IV = 2.2, 2.2, 2.0, 2.3, palp = 2.5. Abdomen 21.5 long, 16.0 wide. Spinnerets: PMS, 1.8 long, 0.7 wide, 0.4 apart; PLS, 2.3 basal, 1.6 middle, 2.5 distal; mid-widths (lateral), 1.9, 1.4, 0.9, respectively. Carapace: length to width 1.04. Fovea: shallow, 2.3 wide. Eyes: tubercle 0.6 high, 2.4 long, 2.8 wide. Clypeus 0.6. Anterior eye row procurved, posterior straight. Eye sizes and inter-distances: AME 0.6, ALE 0.6, PME 0.4, PLE 0.6, AME–AME 0.6, AME–ALE 0.5, AME–PME 0.3, ALE–ALE 2.0, ALE–PME 0.7, PME–PME 1.6, PME–PLE 0.08, PLE–PLE 2.1, ALE–PLE 0.4, AME–PLE 0.4. Ratio of eye group width to length 2.0. Maxillae: length to width: 2.1. Cuspules: 90–100 spread over ventral inner heel. Labium: 2.1 long, 2.2 wide, with 70–90 cuspules spaced by one diameter from each other on the anterior third. Labio-sternal groove deep, narrow, sigilla not evident. Chelicerae: basal segments with ten teeth decreasing in size from distal to basal portion. Sternum: 6.9 long, 6.2 wide. Legs: leg formula: I=IV II III. Scopula: tarsi I–III fully scopulate, IV basal half divided by row of 6–7 setae. Metatarsi I–II 4/5 scopulate; III 2/3, IV 1/3 distal scopulate. IV divided by three wide row of setae. Urticating hairs type II (0.53 to 0.61 long, 0.015 wide) on abdomen dorsum. Genitalia: paired diverging spermathecae tapering from the basally to the apex, folding on medially and with a strong constriction forming two (more rarely one or three) distal lobes (Figs 87–88). Color pattern: carapace, chelicerae, legs and palps brown with light brown setae, except for femora, patellae and tibiae of leg I-II and patellae and tibiae of leg III, blackish ventrally and prolaterally and tibiae and metatarsi of leg IV, darkened ventrally. Sternum, coxae, labium, maxillae black. Longitudinal stripes on dorsum of femora, patellae, tibiae and metatarsi whitish, more conspicuous on the patellae. Distal femora, patellae, tibiae and metatarsi with white rings. Tarsi with a dorsal “U” shaped orange stripe. Abdomen dorsum brown with long light brown setae; a longitudinal large darker stripe on its center. Ventrally black (Fig. 99).

#### Redescription.

Male (MZSP 36884) from Rio Formoso, state of Pernambuco, Brazil. Carapace 10.4 long, 10.0 wide, chelicerae 5.7. Legs (femur, patella, tibia, metatarsus, tarsus, total): I: 13.2, 5.8, 10.9, 11.0, 5.3, 46.2. II: 11.9, 5.3, 9.7, 9.6, 4.9, 41,4. III: 9.8, 4.5, 7.6, 8.3, 3.9, 34.1. IV: 11.7, 4.7, 10.2, 11.0, 4.3, 41.9. Palp: 7.1, 3.9, 5.3, –, 2.2, 18.5. Mid-widths (lateral): femora I–IV = 1.8, 1.7, 2.0, 1.8, palp = 1.3; patellae I–IV = 1.8, 1.9, 1.7, 1.9, palp = 1.6; tibiae I–IV = 1.5, 1.4, 1.5, 1.6, palp = 1.5; metatarsi I–IV = 1.1, 1.0, 1.1, 1.1; tarsi I–IV = 1.1, 1.3, 1.2, 1.1, palp = 1.4. Abdomen 11.2 long, 7.6 wide. Spinnerets: PMS, 1.4 long, 0.5 wide, 0.2 apart; PLS, 2.3 basal, 1.1 middle, 1.7 distal; mid-widths (lateral), 0.8, 0.6, 0.5, respectively. Carapace: length to width 1.01. Fovea: shallow, 1.6 wide. Eyes: tubercle 0.4 high, 1.6 long, 2.1 wide. Clypeus 0.5. Anterior eye row procurved, posterior straight. Eye sizes and inter-distances: AME 0.5, ALE 0.5, PME 0.4, PLE 0.5, AME–AME 0.6, AME–ALE 0.3, AME–PME 0.1, ALE–ALE 1.3, ALE–PME 0.5, PME–PME 1.2, PME–PLE 0.08, PLE–PLE 1.3, ALE–PLE 0.3, AME–PLE 0.4. Ratio of eye group width to length 2.0. Other characters as in female, except: maxillae: length to width: 2.1. Cuspules: 75–80 spread over ventral inner heel. Labium: 1.2 long, 1.6 wide, with 50 cuspules spaced by one diameter from each other on the anterior third center. Labio-sternal groove shallow, flat, with two sigilla. Chelicerae: basal segments with eigth teeth decreasing in size from distal to basal portion. Sternum: 5.0 long, 4.7 wide. Legs: leg formula: I IV II III. Scopula: tarsi I–IV fully scopulate, IV divided by three row of setae. Metatarsi I 4/5 scopulate; II 2/3 scopulate; III 1/2 distal scopulate; IV 1/3 distal scopulate. IV divided by three wide row of setae. Tibial spurs. Legs: I, single, 0.6 long, 1.1 wide, with numerous spiniform setae on its tip (Fig. 85); II, single, without elevation, formed by numerous spiniform setae (Fig. 86). Urticating hairs type II (0.88 to 0.95 long, 0.015 wide) on abdomen dorsum. Palp: embolus 2.6 long, with a 60° curvature to the retrolateral side. Embolus basal, middle and distal width of 0.5, 0.1 and 0.05, respectively. Tegulum 1.1 long, 1.5 wide. (Figs 81–83). Cymbium: spiniform process 0.8 long, 0.4 wide on the apex (fig. 84). Color pattern as in female, except for legs dorsally and ventrally brown with light brown setae; coxa I with distal ventro-retrolateral margin, II with distal half and III–IV completely brown with light brown setae; dorsum of abdomen with a longitudinal stripe of reddish urticating hairs (Fig. 100).

#### Distribution.

Brazil: From state of Ceará southwards to the state of Alagoas (Fig. 169).

#### Spermathecae variation.

Typical spermatheca diverges and tapers from base to apex, folds after its middle and has a strong constriction forming two distal lobes (Figs 87, 119, 121). More rarely, there is one lobe on both (Figs 88, 120) spermathecae or even three lobes (Fig. 122). Relative length and width vary, mainly in smaller specimens (Figs 119–122). However, even small spermathecae are already folded after their middle.

#### Natural history.

Individuals of *Iridopelma hirsutum* are known to inhabit remnants of Brazilian Atlantic rainforest. Of eight specimens collected in Reserva Biológica de Saltinho, state of Pernambuco, and Estação Ecológica de Murici, state of Alagoas, six were found inside or close to retreats on leaves and two adult females were walking on the inner walls of an abandoned house (Fig. 106). They normally build a retreat with two leaves connected with silk threads (Figs 101, 102, 104–105) on different plants such as Piperaceae, Melastomataceae, Palmae (Fig. 101), “Guapeba” (Sapotaceae) (Fig. 105) and other not identified (Figs 102, 104). Ocasionally, they can be found with retreats in a single leaf, as in rolled Heliconiaceae leaf (Fig. 103). Retreat was above ground 0.5 to 1.0 m, but this was affected by collector’s inability to search for specimens high in the vegetation. No adult males or females with eggsacs were found in the excursion period (August).

#### Color pattern ontogeny.

Juveniles have most of the carapace, chelicerae and legs metallic green (Figs 95–96). Coxae, trochantera, basal femora dorsally and parts of the chelicerae are light brown. The abdomen is black with a light central longitudinal large spot having zigzag edges and a longitudinal central black stripe over a more reddish area. The sternum, coxae, labium and abdomen are ventrally black. As the juveniles mature, the metallic green coloration changes to brown (Fig. 97) and the central spot on dorsal abdomen becomes totally reddish (Fig. 98). In the adult females, parts of the abdominal pattern remain, the longitudinal central black stripe is broader, and the reddish area becomes inconspicuous (Fig. 99). Adult males also retain an abdominal pattern, and the reddish area is normally conspicuous (Fig. 100).

#### Remarks.

*Avicularia palmicola* Mello-Leitão, 1945, holotype female from Brazil, Paraíba, Mumbaba, Aristóteles Silva, collected in coconut tree (MNRJ 2328), examined. The specimen is an aviculariine female with first eye row procurved and spermathecae having a single fold and bilobed at distal portion. These characters agree with *Iridopelma* and spermathecae shape is typical of *Iridopelma hirsutum*. Therefore, *Avicularia palmicola* Mello-Leitão, 1945 is transferred to *Iridopelma* and considered a junior-synonym of *Iridopelma hirsutum* Pocock, 1901 syn. n.

### Iridopelma zorodes

(Mello-Leitão, 1926)

http://species-id.net/wiki/Iridopelma_zorodes

Figs 107 –118 123–126 169

Typhochlaena zorodes Mello-Leitão 1926:322, f. 9–10.
Avicularia zorodes : Petrunkevitch 1939:289.
Iridopelma zarodes : Smith 1993:18; Peters 2003:210, f. 847–848.
Iridopelma zorodes : Platnick 2012.

#### Diagnosis.

The males resemble those of *Iridopelma vanini* sp. n.and *Iridopelma katiae* sp. n. by leg I and IV having similar length (leg IV/I length = 0.93, SD = 0.01). It differs from *Iridopelma vanini* sp. n. by the shorter embolus (Figs 107–109). It differs from *Iridopelma katiae* sp. n. by general brown pattern and lack of a reddish pattern on abdomen dorsally (Fig. 117). Females differ from all other species by two straight to slightly curved spermathecae tapering little from base to apex, not folding and with a very slight constriction forming a single distal lobe (Fig. 112).

#### Types.

Holotype male of *Typhochlaena zorodes* [dry, pinned] from Bahia, O. Torres, MNRJ 1408 [MLPC 900], not located (see Silva-Moreira et al. 2010).

#### Additional material examined.

BRAZIL: *Sergipe*: Areia Branca, Parque Nacional Serra de Itabaiana [10°44'S, 37°22'W], 1 male, A. C. M. Fernandes, 12 November 1996 (IBSP 11761); 1 female, R. Bertani, A. D. Brescovit, A. B. Bonaldo, September 1999 (IBSP 9421); 1 female, same data (IBSP 8545); 1 male, same data (IBSP 8543); 1 immature, same data (IBSP 8722); 1 immature, same data (IBSP 8954); 1 female, I. Matos, 1 November 1992 (IBSP 11760); Lagarto [10°54'S, 37°39'W], 1 female, Maxwell ded. September 1999 (IBSP 8026); Santa Luzia do Itanhy, Mata do Crasto [11°23'S, 37°24'W], 1 female, R. Bertani, A. D. Brescovit, A. B. Bonaldo, September 1999 (IBSP 8782); Santo Amaro das Brotas [10°46'S, 37°03'W], no collector data, 23 March 1978, inside bromeliad (MZSP 10847); São Cristóvão [11°00'S, 37°13'W], 1 female, A. D. Brescovit, 6 December 1996 (IBSP 9664); *Bahia*: Acajutiba [11°39'S, 38°01'W], 1 male, 1 female, E. Boaventura, 27 March 1991 (MZSP 32159, col. Bock. 696–697); 1 female, same data (MZSP 32160, col. Bock. 698); 5 females, 17 immatures, same collector, 18 April 1991 (MZSP 32181, col. Bock. 716, 800–820); 3 females, 2 immatures, same data (MZSP 32180, col. Bock. 795–799); Elísio Medrado, RPPN Jequitibá (12°52'3.20"S, 39°28'9.09"W) 1 female, R. H. Nagahama, R. Bertani, C. S. Fukushima, 7 October 2007, at night, inside bromeliad (MNRJ 06253); Salvador, Alphaville [12°56'S, 38°21'W], 1 male, G. G. Montingelli, 11–29 November 2001 (IBSP 9717); Ondina [13°00'S, 38°30'W] 1 immature, T. B. Nunes Ded. December 1982 (IBSP 7906); 1 male (REF 42901C); Mata de São João, RPPN Camarujipe [12°31 S, 38°02'W], 1 male, C. S. Fukushima, R. Bertani and R. H. Nagahama, 4 October 2007, at night on a leaf (MNRJ 06254); *Paraná*: Curitiba [25°25'S, 49°16'W], 1 immature, F. Cominese (MZSP 32161, Bock. 695) (prabably mislabeled).

#### Description.

Female (IBSP 11760) from Areia Branca, Sergipe, Brazil. Carapace 12.8 long, 12.0 wide, chelicerae 6.3. Legs (femur, patella, tibia, metatarsus, tarsus, total): I: 10.8, 6.4, 8.1, 8.4, 4.1, 37.8. II: 9.8, 5.6, 6.9, 6.8, 3.6, 32.7. III: 8.4, 4.6, 6.0, 6.7, 3.4, 29.1. IV: 10.2, 5.3, 8.3, 8.8, 3.7, 36.3. Palp: 7.6, 4.4, 5.0, –, 5.3, 22.3. Mid-widths (lateral): femora I–IV = 2.3, 2.3, 2.4, 2.1, palp = 1.8; patellae I–IV = 2.4, 2.4, 2.4, 2.4, palp = 2.1; tibiae I–IV = 2.2, 2.1, 1.9, 2.1, palp = 1.9; metatarsi I–IV = 1.6, 1.5, 1.5, 1.4; tarsi I–IV = 1.8, 1.8, 1.8, 1.7, palp = 1.8. Abdomen 12.8 long, 9.5 wide. Spinnerets: PMS, 1.3 long, 0.7 wide, 0.3 apart; PLS, 1.6 basal, 1.0 middle, 1.9 distal; mid-widths (lateral), 1.1, 0.9, 0.7, respectively. Carapace: length to width 1.07. Fovea: deep, 2.1 wide. Eyes: tubercle 0.9 high, 1.6 long, 2.6 wide. Clypeus 0.4. Anterior eye row procurved, posterior straight. Eye sizes and inter-distances: AME 0.5, ALE 0.6, PME 0.4, PLE 0.5, AME–AME 0.4, AME–ALE 0.3, AME–PME 0.2, ALE–ALE 1.6, ALE–PME 0.5, PME–PME 1.4, PME–PLE 0.1, PLE–PLE 2.1, ALE–PLE 0.2, AME–PLE 0.4. Ratio of eye group width to length 2.2. Maxillae: length to width: 1.8. Cuspules: 90–100 spread over ventral inner heel. Labium: 1.2 long, 2.1 wide, with 80–100 cuspules spaced by one diameter from each other on the anterior third. Labio-sternal groove deep, narrow, with two sigilla. Chelicerae: basal segments with nine teeth decreasing in size from distal to basal portion. Sternum: 5.9 long, 4.8 wide. Legs: leg formula: I=IV II III. Scopula: tarsi I–IV fully scopulate, IV with some sparse setae. Metatarsi I–II 4/5 scopulate; III 2/3, IV 1/3 distal scopulate. IV divided by three wide row of setae. Urticating hairs type II (0.40 to 0.45 long, 0.01 wide) on the abdomen dorsum. Genitalia: paired straight to slightly curved spermathecae tapering slightly from base to apex, not folding, with very slight constriction forming single distal lobe (Fig. 112). Color pattern: carapace, chelicerae, legs and palps brown with light brown hairs; metatarsi, tibiae and patellae ventrally dark. All tarsi dorsally with a “U” shaped orange stripe. Sternum, labium and maxillae black. Coxae I black, except by brown retrolateral apical third; Coxae II–IV basal half black, distal half brown. Longitudinal stripes on dorsum of femora, patellae, tibiae and metatarsi light. Distal femora, patellae, tibiae and metatarsi with white rings. Abdomen venter black; lateral light brown; dorsal central area dark with reddish urticating hairs (Fig. 116).

#### Redescription.

Male (MNRJ 06254) from Mata de São João, Camurujipe, state of Bahia, Brazil. Carapace 8.0 long, 7.6 wide, chelicerae 4.5. Legs (femur, patella, tibia, metatarsus, tarsus, total): I: 10.2, 4.7, 8.1, 8.3, 4.0, 35.3. II: 9.3, 4.1, 7.3, 7.5, 3.5, 31.7. III: 7.6, 3.6, 5.8, 6.4, 3.2, 26.6. IV: 9.5, 3.7, 7.9, 8.7, 3.4, 33.2. Palp: 5.6, 3.0, 4.1, –, 2.1, 14.8. Mid-widths (lateral): femora I –IV = 1.4, 1.5, 1.5, 1.4, palp = 1.2; patellae I–IV = 1.6, 1.5, 1.5, 1.8, palp = 1.3; tibiae I–IV = 1.4, 1.2, 1.1, 1.3, palp = 1.3; metatarsi I–IV = 0.9, 0.9, 0.9, 0.8; tarsi I–IV = 1.1, 1.1, 1.0, 1.0, palp = 1.2. Abdomen 8.7 long, 4.7 wide. Spinnerets: PMS, 0.9 long, 0.4 wide, 0.1 apart; PLS, 1.2 basal, 0.8 middle, 1.1 distal; mid-widths (lateral), 0.6, 0.6, 0.5, respectively. Carapace: length to width 1.05. Fovea: shallow, 1.3 wide. Eyes: tubercle 0.5 high, 1.4 long, 1.7 wide. Clypeus 0.2. Anterior eye row procurved, posterior straight. Eyes sizes and inter-distances: AME 0.5, ALE 0.3, PME 0.2, PLE 0.3, AME–AME 0.3, AME–ALE 0.1, AME–PME 0.1, ALE–ALE 1.1, ALE–PME 0.3, PME–PME 1.1, PME–PLE 0.08, PLE–PLE 1.2, ALE–PLE 0.2, AME–PLE 0.2. Ratio of eye group width to length 2.1. Other characters as in female, except: maxillae: length to width: 1.8. Cuspules: 90–100 spread over ventral inner heel. Labium: 0.8 long, 1.5 wide, with 70–80 cuspules spaced by one diameter from each other on the anterior third center. Labio-sternal groove shallow, flat, with two sigilla. Chelicerae: basal segments with eleven teeth decreasing in size from distal to basal portion. Sternum: 3.9 long, 3.3 wide. Legs: leg formula: I=IV II III. Scopula: Tarsi I–IV fully scopulate, IV with some sparse setae. Metatarsi I–II 2/3 scopulate; III 2/3 distal scopulate; IV 1/3 distal scopulate. IV divided by four wide row of setae. Tibial spur. Legs: I, single, 0.8 long, 1.0 wide (Fig. 110); II, 0.2 long, 0.7 wide (Fig. 111); both with numerous spiniform setae on tips. Urticating hairs type II (0.75 to 0.85 long, 0.015 to 0.02 wide) on the abdomen dorsum. Palp: embolus 2.2 long, with a 115° curvature to the retrolateral side. Embolus basal, middle and distal width of 0.4, 0.2 and 0.03, respectively. Tegulum 0.8 long, 1.2 wide. (Figs 107–109). Cymbium: spiniform process 0.4 long, 0.4 wide on the apex.

#### Distribution. 

Brazil: states of Sergipe and Northern Bahia (Fig. 169).

#### Spermathecae variation.

Typical spermatheca is straight, or diverges little from its base; has no fold and apical portion is dilated (Figs 112, 123). Some spermathecae are shorter and taper considerably (Fig. 124), whereas others are long and curved distally (Fig. 126). The spermatheca of a specimen from Elísio Medrado, state of Bahia, is partially divided distally (Fig. 125). It is from the southermost distribution of the species and shows a different color pattern as well (Fig. 118).

#### Natural History.

Individuals of *Iridopelma zorodes* inhabits remnants of Brazilian Atlantic rainforest. My own field observations in state of Sergipe (Areia Branca, Parque Nacional Serra de Itabaiana) and Santa Luzia do Itanhy (Mata do Crasto), indicate that individuals make retreats similar to those of *Iridopelma hirsutum*, i. e., leaves connected with silk threads. Eventually, they were found inside bromeliads. An adult male was found on a leaf in Camurujipe, São João da Mata, state of Bahia, in October.

#### Color pattern ontogeny.

As *Iridopelma hirsutum*, but adult males and females lack or have an inconspicuous abdominal pattern (Figs 113–118).

#### Remarks.

Even though the holotype is lost (Silva-Moreira et al. 2010), Mello-Leitão’s (1926) description provides some information that allows conspecific recognition, mainly leg I and IV of similar length, absence of a pattern on abdomen dorsum and type locality state of Bahia. Therefore, I consider this species valid.

### Iridopelma vanini sp. n.

urn:lsid:zoobank.org:act:8302CBCC-2045-4E2B-943E-2FE15BFB7F5E

http://species-id.net/wiki/Iridopelma_vanini

Figs 127 –133 150–152 169

#### Diagnosis.

Males resemble those of *Iridopelma zorodes* and *Iridopelma katiae* sp. n. by leg I and IV having similar length (leg IV/I length = 0.98, SD = 0.02). It differs from *Iridopelma zorodes* and *Iridopelma katiae* sp. n. by longer embolus (Figs 127–129). Additionally, it differs from *Iridopelma katiae* sp. n. by a general brown pattern. Female resembles those of *Iridopelma marcoi* sp. n. by longer spermathecae folded twice (Fig. 132), and differs by having type II urticating setae on abdomen dorsum.

#### Etymology.

The specific name is a patronym in honour of Professor Sérgio Antonio Vanin, a Brazilian entomologist. As advisor of some arachnologists, myself included, he contributed to the development of modern Brazilian arachnology.

#### Types.

Holotype female (IBSP Ref. 74.595), Brazil, state of Piauí, Parnaíba [2°53'S, 41°41'W], 5 m a.s.l., R. Bertani, November 1994; Paratype male (IBSP 11328), Brazil, state of Maranhão, Barreirinhas, Parque Nacional dos Lençóis Maranhenses [2°41'S, 42°55'W), 32 m a.s.l., Equipe Biota, 12–18 October 2001.

#### Additional material examined.

BRAZIL: *Pará*: Rio Tocantins, margem direita, Chiqueirão [4°20'S, 49°25'W], B. M. Mascarenhas, 1 April 1984 (MPEG-ARA 005080); *Maranhão*: Barreirinhas, Parque Nacional dos Lençóis Maranhenses [2°41'S, 42°55'W), 32 m a.s.l., Equipe Biota, 12–18 October 2001, 1 male (IBSP 11346), 1 female (IBSP 11343), 1 female (IBSP 11330), 1 female, 1 immature (IBSP 11345), 1 female, 2 immatures (IBSP 11342); *Piauí*: Castelo do Piauí, E. C. B. Rochas Ornamentais (5°13'50.8"S, 41°42'01.1"W), F. M. Oliveira-Neto, 27 October 2005, 1 male (MPEG-ARA 002297); José de Freitas, Nazareth Eco Resort [4°46'S, 42°34'W], S. Brasil, 3 males (IBSP 11349); *Tocantins*: Colinas de Tocantins [8°03'S, 48°28'W], W. R. Soares, 10 December 1968, 1 female (IBSP 3918).

#### Description.

Holotype female (IBSP Ref. 74.595). Carapace 21.3 long, 20.0 wide, chelicerae 11.0. Legs (femur, patella, tibia, metatarsus, tarsus, total): I: 17.4, 9.8, 13.1, 11.9, 6.5, 58.7. II: 15.9, 9.1, 11.9, 11.2, 5.9, 54.0. III: 14.0, 8.5, 10.6, 10.7, 5.8, 49.6. IV: 16.5, 9.1, 13.5, 13.9, 5.9, 58.9. Palp: 12.0, 7.0, 8.0, –, 8.1, 35.1. Mid-widths (lateral): femora I–IV = 4.2, 4.2, 4.5, 4.2, palp = 3.3; patellae I–IV = 4.0, 4.3, 3.9, 3.8, palp = 3.4; tibiae I–IV = 3.5, 3.3, 3.1, 3.5, palp=3.0; metatarsi I–IV = 2.6, 2.7, 2.4, 2.4; tarsi I–IV = 2.9, 2.9, 2.9, 2.9, palp = 3.0. Abdomen 22.1 long, 16.9 wide. Spinnerets: PMS, 2.2 long, 1.3 wide, 0.5 apart; PLS, 3.7 basal, 2.5 middle, 3.8 distal; mid-widths (lateral), 2.1, 1.9, 1.4, respectively. Carapace: length to width 1.06. Fovea: deep, 4.4 wide. Eyes: tubercle 1.0 high, 2.5 long, 3.5 wide. Clypeus 1.4. Anterior eye row procurved, posterior slightly recurved. Eye sizes and inter-distances: AME 0.6, ALE 0.7, PME 0.4, PLE 0.7, AME–AME 1.0, AME–ALE 0.5, AME–PME 0.4, ALE–ALE 2.4, ALE–PME 0.9, PME–PME 2.1, PME–PLE 0.2, PLE–PLE 2.7, ALE–PLE 0.5, AME–PLE 0.7. Ratio of eye group width to length 2.1. Maxillae: length to width: 2.2. Cuspules: 100–150 spread over ventral inner heel. Labium: 2.5 long, 3.0 wide, with ca. 100 cuspules spaced by one diameter from each other on the anterior third. Labio-sternal groove deep, narrow, sigilla not evident. Chelicerae: basal segments with ten teeth decreasing in size from distal to basal portion. Sternum: 9.9 long, 7.5 wide. Legs: leg formula: I=IV II III. Scopula: tarsi I–IV fully scopulate. Metatarsi I–II 4/5 scopulate; III 2/3, IV 1/3 distal scopulate. IV divided by five wide row of setae. Urticating hairs type II (0.65 to 0.70 long, 0.02 wide) on abdomen dorsum. Genitalia: paired long and converging spermathecae tapering strongly from base to apex, double folded and with strong constriction forming two (more rarely one or three) distal lobes (Fig. 132). Color pattern: carapace, chelicerae and dorsum of legs and palps covered with short setae having green/blue iridescence and with abundant longer light brown setae giving a brownish appearance. Tarsi dorsally with a “U” orange stripe. Femora ventrally of the same color as the dorsum; patellae, tibiae, metatarsi and tarsi ventrally blackish. Coxae, labium, sternum and maxillae black. Longitudinal stripes on dorsum of femora, patellae, tibiae and metatarsi whitish, conspicuous. Distal femora, patellae, tibiae and metatarsi with white rings. Abdomen dorsum and lateral light-brown with long setae of same color. Central dorsal area blackish, forming a pattern, with long setae having basal part blackish, distal light brown. Urticating hairs reddish. Abdomen ventrally black (Fig. 133).

#### Description.

Paratype male (IBSP 11328). Carapace 17.7 long, 17.4 wide, chelicerae 9.6. Legs (femur, patella, tibia, metatarsus, tarsus, total): I: 17.5, 9.1, 13.9, 13.6, 7.3, 61.4. II: 16.4, 8.3, 13.0, 12.7, 6.5, 56.9. III: 14.0, 7.0, 11.0, 11.6, 5.8, 49.4. IV: 16.7, 7.7, 14.4, 14.9, 6.3, 60.0. Palp: 9.9, 5.8, 7.7, –, 3.6, 27.0. Mid-widths (lateral): femora I–IV = 3.7, 3.6, 3.7, 3.4, palp = 2.5; patellae I–IV = 3.6, 3.5, 3.5, 3.3, palp = 2.6; tibiae I–IV = 3.0, 2.9, 2.9, 2.7, palp = 2.4; metatarsi I–IV = 2.2, 2.1, 1.9, 1.8; tarsi I–IV = 2.4, 2.5, 2.1, 2.3, palp = 2.4. Abdomen 19.3 long, 13.1 wide. Spinnerets: PMS, 1.7 long, 0.8 wide, 0.3 apart; PLS, 2.9 basal, 2.3 middle, 2.9 distal; mid-widths (lateral), 1.3, 1.1, 0.8, respectively. Carapace: length to width 1.01. Fovea 2.1 wide. Eyes: tubercle 0.9 high, 1.9 long, 3.0 wide. Clypeus 1.0. Anterior eye row procurved, posterior slightly recurved. Eye sizes and inter-distances: AME 0.6, ALE 0.7, PME 0.5, PLE 0.7, AME–AME 0.7, AME–ALE 0.5, AME–PME 0.3, ALE–ALE 1.9, ALE–PME 0.6, PME–PME 1.8, PME–PLE 0.3, PLE–PLE 2.3, ALE–PLE 0.2, AME–PLE 0.6. Ratio of eye group width to length 2.0. Other characters as in female, except: maxillae: length to width: 2.3. Cuspules: 150–200 spread over ventral inner heel. Labium: 2.3 long, 2.9 wide, with 150–200 cuspules spaced by one diameter from each other on the anterior third center. Labio-sternal groove shallow, flat, sigilla not evident. Sternum: 8.2 long, 6.8 wide. Legs: leg formula: I=IV II III. Tibial spurs. Legs: I, single, 0.8 long, 2.2 wide (Fig. 130); II, single, 0.5 long, 2.1 wide (Fig. 131); both with numerous spiniform setae on their tips. Urticating hairs type II (1.13 long, 0.02 wide) on the abdomen dorsum. Palp: embolus 4.9 long, with a 135° curvature to the retrolateral side. Embolus basal, middle and distal width of 0.9, 0.3 and 0.04, respectively. Tegulum 1.5 long, 2.3 wide. (Figs 127–129). Cymbium: spiniform process 1.0 long, 0.7 wide on apex. Color pattern and setation mostly as in female. Dorsal abdominal pattern not as conspicuous as in female.

#### Distribution.

Brazil, states of Piaui, Maranhão and possibly Tocantins and Pará (Fig. 169). There is a single record for both Tocantins (Colinas de Tocantins) and Pará (Tocantins River). These specimens are smaller than those from Maranhão and Piauí, and the specimen from Pará has both male palpal bulb emboli broken, therefore its identification is only tentative.

#### Spermathecae variation.

Typical spermatheca is long, converging, tapers strongly from base to apex, double folded and with a strong constriction forming two distal lobes (Figs 132, 151). In some specimens the apical region ends in a single lobe, that can be partially divided (Fig. 150) or in two lobes with one partially or completely divided, in this case resulting in multilobular spermathecae (Fig. 152).

#### Natural history. 

Little is known of this species. The holotype female was collected with an eggsac in November, under a fallen tree trunk in a moderately anthropized area in Parnaíba, state of Piaui. Other specimens were collected in Barreirinhas, State of Maranhão. Both localities are on coastal region characterized by huge sandy dunes and sparse vegetation. Other localities, as Jose de Freitas (Carvalho and Avelino 2010) and Castelo do Piaui, both in state of Piaui, as well as Colinas do Tocantins, state of Tocantins, are chiefly covered by cerrado, a savannah-like vegetation.

#### Color pattern ontogeny.

No data available.

### Iridopelma katiae sp. n.

urn:lsid:zoobank.org:act:E4ED25DB-B675-4BC5-9E5D-E5A9C9159DB6

http://species-id.net/wiki/Iridopelma_katiae

Figs 134 [Fig F26] –149 153–155 169

#### Diagnosis.

Male resemble those of *Iridopelma zorodes* and *Iridopelma vanini* sp. n. in similar leg I and IV lengths (leg IV/I length =1.04, SD= 0.02). It differs from *Iridopelma zorodes* by blackish coloration with a reddish pattern on abdomen dorsum (Fig. 143). It differs from *Iridopelma vanini* sp. n. by shorter embolus (Figs 134–136). Female differs from those of all species in spermathecal morphology, two straight to slightly curving spermathecae tapering little from base to apex, lacking folds and with constriction forming two to multiple distal lobes (Fig. 139). Additionally, females have a general blackish coloration with a reddish pattern on abdomen dorsum (Fig. 142).

#### Etymology.

The specific name is a matronym in honour of my wife, Kátia de Mendonça Faria, who illustrated several plates for spider taxonomy papers, this work included.

#### Types.

Holotype female, Brazil, *Bahia*, Mucugê, Parque Nacional da Chapada Diamantina (12°45'7.00"S, 41°30'2.18"W), 1399 m a.s.l., R. Bertani, C. S. Fukushima and R. H. Nagahama, 18 February 2008 (MZSP 36887); Paratype male (12°45'3.77"S, 41°30'4.01"W), 1377 m a.s.l., 17 February 2008 (MZSP 36888), same data; Paratype male (12°45'4.18"S, 41°30'3.73"W), 1366 m.a.s.l, same data (MZSP 36889); Paraype female (12°45' 2.50"S, 41°30'4.15"W), 1388 m.a.s.l, same data, found with *ca*. 100 young (MZSP 36890). All specimens found inside bromeliads (*Vriesea atra*) in a campo rupestre area.

#### Additional material examined.

BRAZIL: *Bahia*: Ibicoara, Serra do Sincorá [13°24'S, 41°15'W], G. Machado, 12 March 2000, 2 females (MZSP 22762); Rio de Contas [13°35'S, 41°48'W], O. A. V. Marques and G. Skuk, 09 October 1991, 1 female, 1 immature (IBSP 10317).

#### Description.

Holotype female (MZSP 36887). Carapace 13.3 long, 12.7 wide, chelicerae 7.7. Legs (femur, patella, tibia, metatarsus, tarsus, total): I: 11.3, 6.5, 8.2, 7.6, 4.6, 38.2. II: 10.0, 6.1, 7.2, 7.0, 4.3, 34.6. III: 9.3, 5.3, 6.4, 6.9, 4.1, 32.0. IV: 10.9, 5.2, 8.8, 9.6, 4.7, 39.2. Palp: 7.9, 4.6, 5.3, –, 5.9, 23.7. Mid-widths (lateral): femora I–IV = 2.4, 2.5, 2.8, 2.4, palp = 2.2; patellae I–IV = 2.7, 2.6, 2.5, 2.5, palp = 2.3; tibiae I–IV = 2.1, 2.1, 2.1, 2.0, palp = 2.1; metatarsi I–IV = 1.9, 1.7, 1.6, 1.6; tarsi I–IV = 2.1, 2.1, 2.1, 2.0, palp = 2.1. Abdomen 18.4 long, 13.0 wide. Spinnerets: PMS, 1.7 long, 0.7 wide, 0.3 apart; PLS, 2.1 basal, 1.4 middle, 1.5 distal; mid-widths (lateral), 1.3, 1.1, 0.8, respectively. Carapace: length to width 1.04. Fovea: shallow, 2.3 wide. Eyes: tubercle 0.5 high, 1.8 long, 2.4 wide. Clypeus 0.6. Anterior eye row procurved, posterior slightly recurved. Eye sizes and inter-distances: AME 0.4, ALE 0.6, PME 0.5, PLE 0.6, AME–AME 0.6, AME–ALE 0.4, AME–PME 0.2, ALE–ALE 1.5, ALE–PME 0.3, PME–PME 1.4, PME–PLE 0.08, PLE–PLE 1.8, ALE–PLE 0.3, AME–PLE 0.5. Ratio of eye group width to length 2.0. Maxillae: length to width: 1.9. Cuspules: 100–120 spread over ventral inner heel. Maxillary lyra absent. Labium: 1.6 long, 2.0 wide, with *ca*. 100 cuspules spaced by one diameter from each other on the anterior third. Labio-sternal groove deep, narrow, sigilla not evident. Chelicerae: basal segments with ten teeth decreasing in size from distal to basal portion. Sternum: 6.2 long, 5.3 wide. Legs: leg formula: I=IV II III. Scopula: Tarsi I–III fully scopulate, IV basal half divided by row of 6–7 setae. Metatarsi I–II 4/5 scopulate; III 2/3, IV 1/3 distal scopulate. IV divided by three wide row of setae. Urticating hairs type II (0.45 to 0.51 long, 0.012 wide) on abdomen dorsum. Genitalia: paired slightly curving inwards spermathecae tapering slightly from base to apex, not folded, with constriction forming two to multiple distal lobes (Fig. 139). Color pattern: carapace, chelicerae, legs and palps black, covered with hairs having a weak golden iridescence. Coxae, labium, sternum, and maxillae black. Longitudinal stripes on dorsum of femora, patellae, tibiae and metatarsi inconspicuous. Distal femora, patellae, tibiae and metatarsi with discrete whitish rings. Abdomen dorsum black with longitudinal large band of long reddish setae on the central area, forming a pattern. Sparse long red setae over lateral area of abdomen. Abdomen ventrally black (Fig. 142).

#### Description.

Paratype male (MZSP 36888). Carapace 12.1 long, 11.2 wide, chelicerae 6.5. Legs (femur, patella, tibia, metatarsus, tarsus, total): I: 11.7, 6.6, 9.1, 10.5, 5.2, 43.1. II: 10.8, 6.0, 8.3, 9.3, 4.8, 39.2. III: 9.5, 4.6, 7.0, 8.2, 4.6, 33.9. IV: 11.4, 5.5, 9.7, 11.0, 4.5, 42.1. Palp: 6.6, 4.4, 4.9, –, 2.6, 18.5. Mid-widths (lateral): femora I–IV = 2.1, 2.0, 2.4, 2.4, palp=1.7; patellae I–IV = 2.1, 2.3, 2.2, 2.2, palp = 2.0; tibiae I–IV = 1.9, 1.8, 1.6, 1.9, palp = 1.7; metatarsi I–IV = 1.3, 1.3, 1.3, 1.2; tarsi I–IV = 1.2, 1.3, 1.5, 1.2, palp = 1.8. Abdomen 12.3 long, 8.1 wide. Spinnerets: PMS, 1.4 long, 0.5 wide, 0.2 apart; PLS, 2.4 basal, 1.2 middle, 1.7 distal; mid-widths (lateral), 0.9, 0.8, 0.6, respectively. Carapace: length to width 1.08. Fovea 1.8 wide. Eyes: tubercle 0.8 high, 1.5 long, 2.1 wide. Clypeus 0.4. Anterior eye row procurved, posterior straight. Eyes size and inter-distances: AME 0.4, ALE 0.4, PME 0.3, PLE 0.4, AME–AME 0.4, AME–ALE 0.3, AME–PME 0.2, ALE–ALE 1.3, ALE–PME 0.4, PME–PME 1.1, PME–PLE 0.1, PLE–PLE 1.6, ALE–PLE 0.3, AME–PLE 0.5. Ratio of eye group width to length 2.1. Maxillae: length to width: 1.6. Cuspules: 120–150 spread over ventral inner heel. Labium: 1.3 long, 2.1 wide, with *ca*. 90 cuspules spaced by one diameter from each other on the anterior third center. Labio-sternal groove shallow, flat, with two sigilla. Chelicerae: basal segments with eleven teeth decreasing in size from distal to basal portion. Sternum: 5.1 long, 4.9 wide. Legs: leg formula: I=IV II III. Scopula: Tarsi I–IV fully scopulate, IV with a row of sparse setae. Metatarsi I–II 4/5 scopulate; III 1/2 distal scopulate; IV 1/3 distal scopulate. IV divided by three wide row of setae. Tibial spurs. Legs: I, single, 0.4 long, 1.2 wide (Fig. 137); II, 0.2 long, 1.1 wide (Fig. 138); both with numerous spiniform setae on their tips. Urticating hairs type II (0.72 to 0.75 long, 0.015 wide) on abdomen dorsum. Palp: embolus 3.2 long, with a 90° curvature to retrolateral side. Embolus basal, middle and distal width of 0.8, 0.2 and 0.04, respectively. Tegulum 1.0 long, 1.5 wide. (Figs 134–136). Cymbium: spiniform process 0.7 long, 0.5 wide on the apex. Color pattern and setation as in female (Fig. 143).

#### Distribution.

Brazil: state of Bahia, endemic to Chapada Diamantina and its immediate surrounding regions, in Espinhaço mountain range (Fig. 169).

#### Spermathecae variation.

Typical spermatheca is slender, slightly curving inwards, tapering little from base to apex, not folded, with a distal constriction forming two to multiple distal lobes (Fig. 139, 153–155). Detected variation concerns shape and number of lobes.

#### Natural history.

Two specimens were found under rocks in Ibicoara (G. Machado, pers. comm.) and in Rio de Contas, both in state of Bahia. I found four specimens in Mucugê, Parque Nacional da Chapada Diamantina, all inside bromeliads (*Vriesea atra*) (Fig. 147). One of the specimens folded a bromeliad leaf and connected it with silk threads, making a retreat (Fig. 148). Inside with the mother were *ca*. 100 small juveniles (Fig. 149). The region where they were found is high (1400 m. a. s. l.) (Fig. 144) and vegetation is sparse, growing over a rocky substratum with shallow soil (campo rupestre) (Figs 145–146). Temperature is high during the day, falling abruptly at night. Water is largely available only during the rainy period. That’s a very distinct environmental condition when compared with the habitat in rainforest of *Iridopelma hirsutum* and *Iridopelma zorodes*, and seems inhospitable, mainly for an arboreal species. In this environment, bromeliads and space under rocks are among the few available shelter for spiders.

Cladistic analysis suggests that a retreat made with tree leaves and silk is primitive for *Iridopelma* species, and bromelicolous habitat in *Iridopelma katiae* sp. n. is derived. A possibility to explain the habit change could be the existence of a primitive forest in the area which became drier more recently in geological time, resulting in grasslands over a rocky or sandy shallow soil and lack of high vegetation. Bromeliads then become the primary (and relatively scarce) available microhabitat with similar conditions and resources, e. g. leaves as substratum and high humidity, necessities for an aviculariine spider. The environmental conditions in campo rupestre areas in Chapada Diamantina could aid in understanding the specialization to endemic bromelicolous lifestyle in other spider species, such as *Pachistopelma* spp.

#### Color pattern ontogeny.

Juveniles have light brown legs, except for black tarsi. Carapace is black and abdomen has a broad central longitudinal red stripe over a black area. Abdomen laterals are light brown (Fig. 140). When the individual grows, it becomes darker and retains the abdominal pattern, now with a longitudinal central black stripe (Fig. 141). Adults males and females are completely black and have a conspicuous abdominal pattern remains (Figs 142–143).

### Iridopelma oliveirai sp. n.

urn:lsid:zoobank.org:act:11BF0C3A-82BB-4337-8441-4446A8E9EB02

http://species-id.net/wiki/Iridopelma_oliveirai

Figs 156 –165 169

#### Diagnosis.

The male differs from those of all other species by very long leg I (leg IV/I length = 0.73) (Fig. 165). The female resembles *Iridopelma hirsutum* by diverging spermathecae folded distally (Fig. 161), but differs by having a grayish carapace and legs and lacking an abdominal pattern (Fig. 164).

#### Etymology.

Specific name is a patronym in honor of Judge João Carlos Sá Moreira de Oliveira, for allowing the author to have access to part of the aviculariine specimens deposited in the Instituto Butantan collections, which were fundamental for the completion of this present work.

#### Types.

Holotype male (IBSP 10100), Brazil, state of Bahia, Central, Toca dos Pilões [11°08'S, 42°06'W], A. D. Brescovit, July 2000, and paratype female (IBSP 8714), at night in “Macambira” bromeliad (*Bromelia laciniosa*), same data.

#### Additional material examined.

Only the types.

#### Description.

Holotype male (IBSP 10100). Carapace 14.7 long, 12.7 wide, chelicerae 7.1. Legs (femur, patella, tibia, metatarsus, tarsus, total): I: 16.5, 7.9, 16.7, 17.8, 7.9, 68.8. II: 14.3, 7.3, 13.4, 14.4, 6.1, 55.5. III: 10.8, 5.6, 9.0, 10.2, 4.4, 40.0. IV: 13.1, 6.2, 12.1, 13.4, 4.8, 49.6. Palp: 9.1, 5.1, 6.9, –, 2.4, 23.5. Mid-widths (lateral): femora I –IV = 2.5, 2.4, 2.3, 2.3, palp = 1.8; patellae I–IV = 2.5, 2.4, 2.3, 2.4, palp = 1.9; tibiae I–IV = 1.9, 1.9, 1.9, 2.0, palp = 1.7; metatarsi I–IV = 1.3, 1.2, 1.2, 1.2; tarsi I–IV = 1.1, 1.2, 1.4, 1.5, palp = 1.6. Abdomen 12.8 long, 6.1 wide. Spinnerets: PMS, 1.1 long, 0.5 wide, 0.2 apart; PLS, 1.7 basal, 1.1 middle, 1.6 distal; mid-widths (lateral), 1.1, 0.9, 0.7, respectively. Carapace: length to width 1.16. Fovea: deep, 2.0 wide. Eyes: tubercle 0.9 high, 1.8 long, 2.5 wide. Clypeus 0.8. Anterior eye row procurved, posterior straight. Eye sizes and inter-distances: AME 0.4, ALE 0.5, PME 0.3, PLE 0.5, AME–AME 0.6, AME–ALE 0.3, AME–PME 0.2, ALE–ALE 1.6, ALE–PME 0.5, PME–PME 1.5, PME–PLE 0.1, PLE–PLE 1.9, ALE–PLE 0.5, AME–PLE 0.5. Ratio of eye group width to length 1.9. Maxillae: length to width: 2.0. Cuspules: 90–100 spread over ventral inner heel. Labium: 1.4 long, 2.3 wide, with *ca*. 40 cuspules. Labio-sternal groove deep, flat, with two sigillae. Chelicerae: basal segments with eleven teeth decreasing in size from distal to basal portion. Sternum: 6.6 long, 5.0 wide. Sigilla: not evident. Legs: leg formula: I II IV III. Scopula: tarsi I–IV fully scopulate. Metatarsi I–II 4/5 fully scopulate; III 3/4 distal scopulate; IV 1/2 distal scopulate. IV divided by three wide row of setae. Tibial spurs. Legs: I, single, 2.0 long, 1.2 wide, with numerous spiniform setae on its tip (Fig. 159); II, single, without elevation, formed by numerous spiniform setae (Fig. 160). Urticating hairs type II (0.73 to 0.77 long, 0.015 wide) on abdomen dorsum. Palp: embolus 2.2 long, with 105° curvature to retrolateral side. Embolus basal, middle and distal width of 0.2, 0.1 and 0.03, respectively. Tegulum 1.2 long, 1.5 wide. (Figs 156–158). Cymbium: Spiniform process 0.6 long, 0.5 wide on apex. Color pattern: carapace, chelicerae, legs and palps brown with light brown setae. Sternum, labium, maxillae and anterior half of coxa I black. Posterior half of coxa I and coxae II–IV brown with light brown setae. Longitudinal stripes on dorsum of femora, patellae, tibiae and metatarsi whitish. Distal femora, patellae, tibiae and metatarsi with white rings. Tarsi with dorsal “U” shaped orange stripe. Abdomen dorsum brown with long light brown setae. Ventrally black (Fig. 165).

#### Description.

Paratype female (IBSP 8714). Carapace 13.2 long, 11.4 wide, chelicerae 6.7. Legs (femur, patella, tibia, metatarsus, tarsus, total): I: 11.0, 6.2, 8.6, 8.0, 4.6, 38.4. II: 9.7, 5.8, 7.6, 7.1, 4.0, 34.2. III: 8.3, 4.8, 5.9, 6.3, 3.8, 29.1. IV: 10.2, 5.2, 8.6, 8.8, 4.0, 36.8. Palp: 7.4, 4.2, 5.0, –, 5.1, 21.7. Mid-widths (lateral): femora I –IV = 2.0, 2.0, 2.3, 2.1, palp = 1.8; patellae I–IV = 2.2, 2.1, 2.1, 2.2, palp = 2.0; tibiae I–IV = 2.0, 1.8, 1.8, 2.1, palp = 1.8; metatarsi I–IV = 1.7, 1.5, 1.5, 1.2; tarsi I–IV = 1.8, 1.7, 1.8, 1.5, palp = 1.9. Abdomen 13.2 long, 8.8 wide. Spinnerets: PMS, 1.6 long, 0.9 wide, 0.2 apart; PLS, 1,8 basal, 1.3 middle, 1.8 distal; mid-widths (lateral), 1.3, 1.1, 0.8, respectively. Carapace: length to width 1.16. Fovea: 2.4 wide. Eyes: tubercle 0.7 high, 1.6 long, 2.3 wide. Clypeus 0.4. Anterior eye row procurved, posterior slightly recurved. Eye sizes and inter-distances: AME 0.4, ALE 0.4, PME 0.4, PLE 0.4, AME–AME 0.5, AME–ALE 0.2, AME–PME 0.5, ALE–ALE 1.7, ALE–PME 0.5, PME–PME 1.4, PME–PLE 0.1, PLE–PLE 1.8, ALE–PLE 0.5, AME–PLE 0.5. Ratio of eye group width to length 2.1. Other characters as in male, except: maxillae: length to width: 1.8. Cuspules: 100–150 spread over ventral inner heel. Labium: 1.5 long, 1.9 wide, with ca. 50 cuspules. Labio-sternal groove deep, narrow, with two sigillae. Chelicerae: basal segments with thirteen teeth decreasing in size from distal to basal portion. Sternum: 5.8 long, 5.1 wide. Legs: leg formula: I=IV II III. Scopula: tarsi I–IV fully scopulate. Metatarsi I–II fully scopulate; III 2/3, IV 1/2 distal scopulate, IV divided by three wide row of setae. Urticating hairs type II (0.57 to 0.61 long, 0.015 wide) on abdomen dorsum. Genitalia: paired diverging spermathecae tapering from base to apex, folded medially, with strong distal constriction forming two distal lobes (Fig. 161). Color pattern as in male, except: carapace, chelicerae, legs and palps grayish with pinkish setae; patellae and tibiae of palps and legs I–II and tibiae and metatarsi of leg III ventrally dark. Sternum, labium, maxillae and coxae black. Abdomen dorsum brown with long pinkish setae.

#### Distribution.

Brazil: known only from Central, State of Bahia (Fig. 169).

#### Natural History. 

Little is known regarding this species. The vegetation in Central, state of Bahia, comprises mainly caatinga (a xerophilous vegetation). One female was found at night in a bromeliad **(***Bromelia laciniosa*).

#### Color pattern ontogeny.

Data for small juveniles is lacking. Large immatures specimens have a pattern similar to *Iridopelma hirsutum* (Figs 162–163). Adult male and female lack or have a very inconspicuous abdominal pattern (Figs 164–165).

### Iridopelma marcoi sp. n.

urn:lsid:zoobank.org:act:4ED0A9EA-7460-4CAE-82B8-EB38B489CBE0

http://species-id.net/wiki/Iridopelma_marcoi

Figs 166 [Fig F32] –169

#### Diagnosis. 

The female resembles that of *Iridopelma vanini* sp. n. by long double folded spermathecae (Fig. 166), but differ by lacking type II urticating setae on abdomen dorsum. Male unknown.

#### Etymology.

The specific name is a patronym in honour of Marco Antonio de Freitas, a Brazilian zoologist and geographer who collected the holotype and some other aviculariine specimens studied in this work.

#### Type.

Holotype female, Brazil, state of Bahia, São Desidério (12°28'52"S, 45°09'10"W), 724 m a.s.l, M. A. Freitas, October 2009, under tree bark, 1 m above the ground, area of carrasco vegetation (MZSP 36891).

#### Additional material examined.

Only holotype.

#### Description.

Holotype female (MZSP 36891). Carapace 14.9 long, 14.6 wide, chelicerae 9.3. Legs (femur, patella, tibia, metatarsus, tarsus, total): I: 12.4, 7.8, 8.9, 8.3, 4.5, 41.9. II: 11.4, 6.9, 8.1, 7.6, 4.4, 38.4. III: 10.0, 6.1, 7.3, 6.5, 4.3, 34.2. IV: 12.2, 6.5, 9.7, 10.1, 4.7, 43.2. Palp: 8.7, 5.5, 5.6, –, 5.9, 25.7. Mid-widths (lateral): femora I–IV = 3.0, 3.1, 3.2, 2.5, palp = 2.4; patellae I–IV = 2.8, 2.8, 2.8, 2.8, palp = 2.4; tibiae I–IV = 2.6, 2.6, 2.5, 2.4, palp = 2.3; metatarsi I–IV = 2.1, 2.0, 2.0, 1.6; tarsi I–IV = 2.2, 2.2, 229, 2.2, palp = 2.2. Abdomen 16.7 long, 11.7 wide. Spinnerets: PMS, 1.9 long, 0.9 wide, 0.3 apart; PLS, 2.3 basal, 1.5 middle, 2.2 distal; mid-widths (lateral), 1.3, 1.2, 1.0, respectively. Carapace: length to width 1.02. Fovea: deep, 2.5 wide. Eyes: tubercle 0.8 high, 2.1 long, 2.6 wide. Clypeus 1.0. Anterior eye row procurved, posterior slightly recurved. Eye sizes and inter-distances: AME 0.5, ALE 0.5, PME 0.4, PLE 0.4, AME–AME 0.6, AME–ALE 0.3, AME–PME 0.1, ALE–ALE 1.8, ALE–PME 0.5, PME–PME 1.6, PME–PLE 0.1, PLE–PLE 2.1, ALE–PLE 0.4, AME–PLE 0.4. Ratio of eye group width to length 2.0. Maxillae: length to width: 2.0. Cuspules: 100–150 spread over ventral inner heel. Labium: 1.9 long, 2.6 wide, with ca. 150 cuspules. Labio-sternal groove deep, narrow, sigilla not evident. Chelicerae: basal segments with ten teeth decreasing in size from distal to basal portion. Sternum: 7.5 long, 6.6 wide. Legs: leg formula: I=IV II III. Scopula: tarsi I–IV fully scopulate. Metatarsi I–II 4/5 scopulate; III 2/3, IV 1/2 distal scopulate. IV divided by three wide row of setae. Urticating hairs lacking on abdomen dorsum. Genitalia: paired long and converging spermathecae tapering strongly from base to apex, double folded and with a strong distal constriction forming one or two distal lobes (Fig. 166). Color pattern: carapace, chelicerae, and femora of legs and palps dark brown covered with dense layer of metallic green setae. Other leg and palp segments dorsally dark brown with brown setae. Tibiae and patellae covered with metallic blue setae prolaterally and retrolaterally; ventrally black. Tarsi with a dorsal “U” orange stripe. Sternum, labium, maxillae and coxae black. Abdomen ventrally black; dorsally dark brown with a large lighter central spot. Longitudinal stripes on dorsum of femora, patellae, tibiae and metatarsi inconspicuous. Distal femora, patellae, tibiae and metatarsi with broad white rings (Fig. 167).

Male: unknown.

#### Distribution.

Brazil: known only from São Desidério, state of Bahia (Fig. 169).

#### Natural history.

The only known specimen was found in cerrado/carrasco vegetation (Fig. 168), under loose tree bark about one meter above the ground (M. A. Freitas, pers. comm.).

#### Color pattern ontogeny.

Only the adult female holotype is known.

#### Cladistics.

Searches using NONA resulted in 12 trees and strict consensus is used (Fig. 177). With X-PEE-WEE, it was found from 1 to 6 trees, depending on the concavity used (Table 2). Cladogram obtained with concavity 6 is shown in Figs 178. The main difference of cladograms obtained with the different strategies comprise the position of clade having *Ephebopus*, *Tapinauchenius* and *Psalmopoeus*. In NONA and X-PEE-WEE with concavities 2–6 that clade is paraphyletic in respect to other aviculariine genera (Figs 177–178); with X-PEE-WEE and concavity 1 Aviculariinae is retrieved as monophyletic. Topologies of clade *Typhochlaena* (*Avicularia* spp.1 (*Pachistopelma*, *Avicularia* spp.2, *Iridopelma*)) were retrieved in all employed search strategies. Resolution of the terminal clade retrieving *Iridopelma* as sister group of *Avicularia* spp.2 was obtained with X-PEE-WEE and concavities 1–5, whereas *Iridopelma* and *Avicularia* spp.2 were in a tricotomy with *Pachistopelma* when using NONA and X-PEE-WEE with concavity 6. Internal relationship of *Typhochlaena* is totally unresolved with NONA, and partially resolved with X-PEE-WEE, independently of the concavity used. Position of *Iridopelma marcoi* sp. n. and *Iridopelma katiae* sp. n. change more markedly. With concavities 1–5 *Iridopelma katiae* sp. n. is sister-group of *Pachistopelma* (*Iridopelma* + *Avicularia* spp.2). With NONA and X-PEE-WEE with concavity 6 it is part of *Iridopelma* clade. *Iridopelma marcoi* sp. n. is part of *Iridopelma* clade only with NONA. With X-PEE-WEE it is always polyphyletic. Its position will be fully discussed below.

**Table 2.** Number of trees obtained, length and fit using different concavities and X-PEE-WEE.<br/>

	**Trees**	**Length**	**FIT**
K = 1	2	221	2945,4
K = 2	6	213	3549,1
K = 3	6	213	3919,8
K = 4	6	213	4172,5
K = 5	1	211	4361,2
K = 6	1	209	4507,4

**Figures 170–175.**
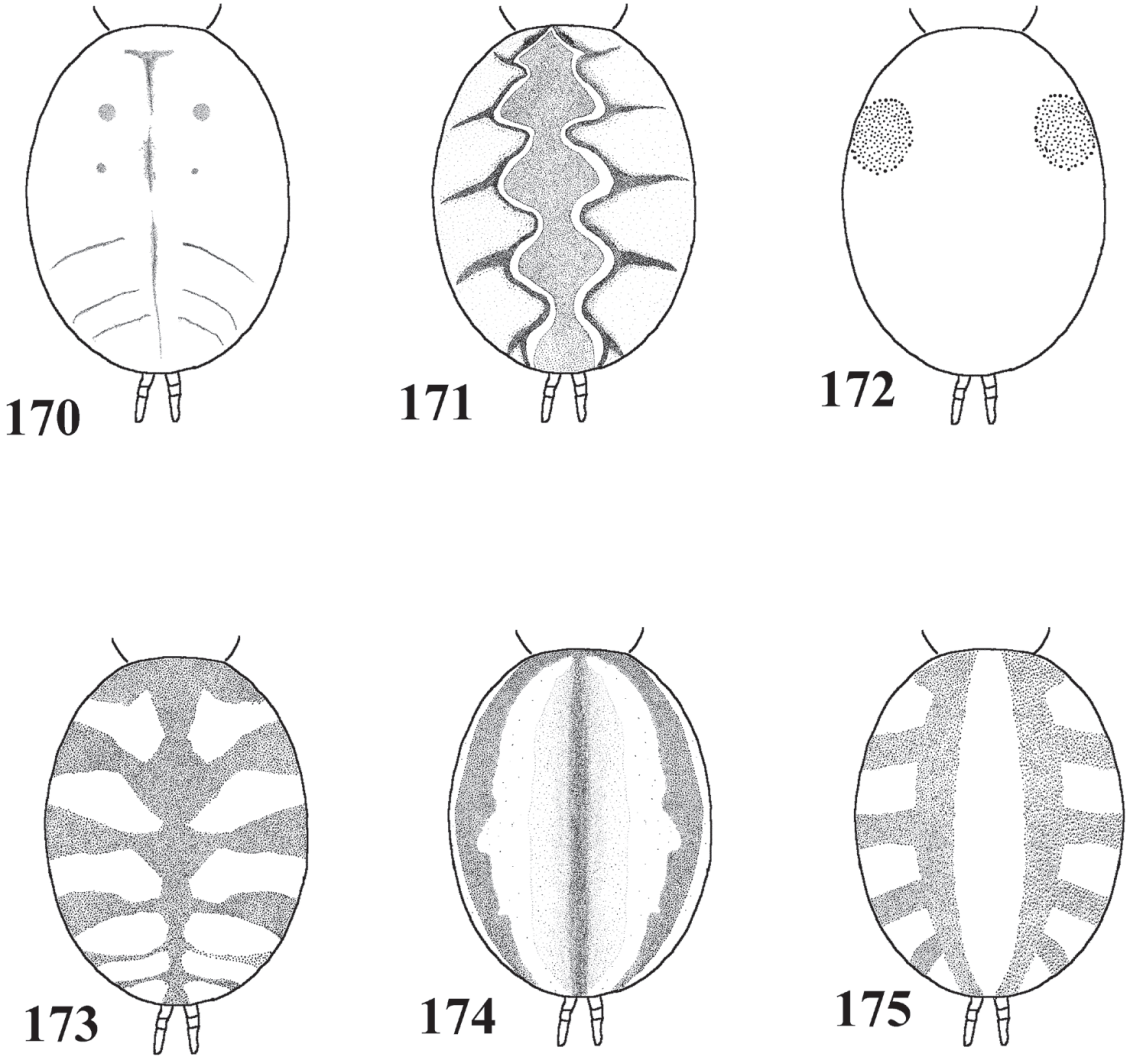
Dorsal abdominal pattern in immatures **170** herringbone (*Pterinochilus* sp.) **171** with a zigzag central longitudinal dark stripe over a clear spot, which is marginated in black and connects to five narrow transversal black stripes (*Poecilotheria* sp.) **172** two median dorso-lateral spots (*Euathlus vulpinus* (Karsch, 1880)) **173** central longitudinal black stripe with 5–6 lateral stripes, connecting or not with the central stripe (*Pachistopelma bromelicola* sp. n.) **174** black with a light central longitudinal large spot having zigzag edges and a longitudinal central black stripe over a more reddish area (*Iridopelma hirsutum* Pocock, 1901) **175** two longitudinal dark stripes with five lateral stripes connecting to them (*Avicularia diversipes* (C. L. Koch, 1842)).

**Figure 176.**
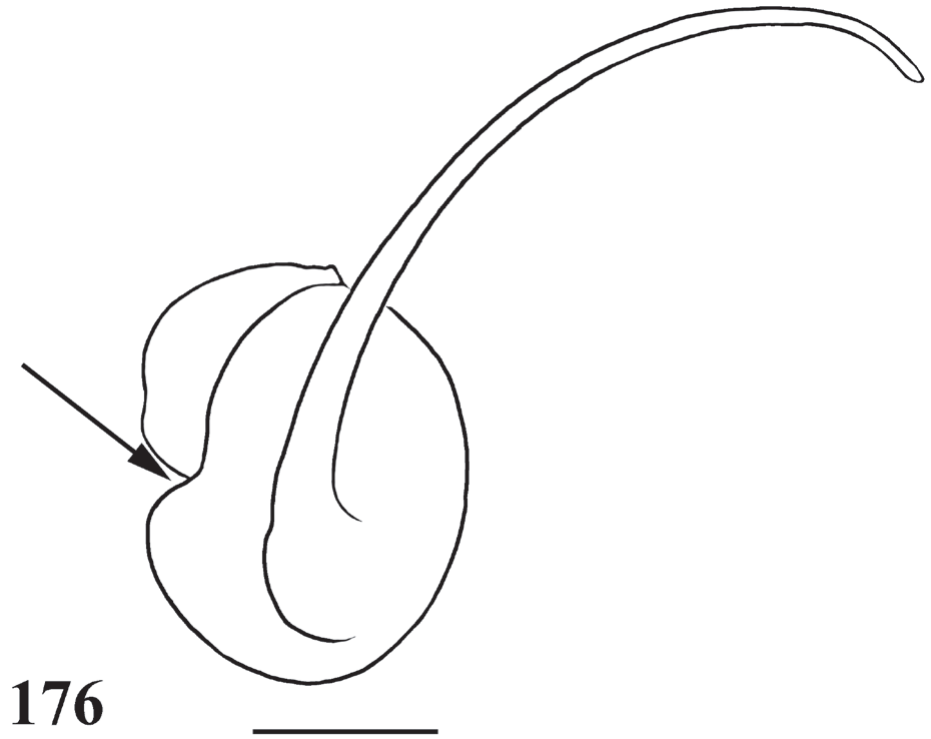
*Avicularia taunayi* (Mello-Leitão, 1920), left male palpal bulb, frontal view. Arrow indicates accentuated depression on retrolateral tegulum. Scale bar = 1 mm.

**Figure 177.**
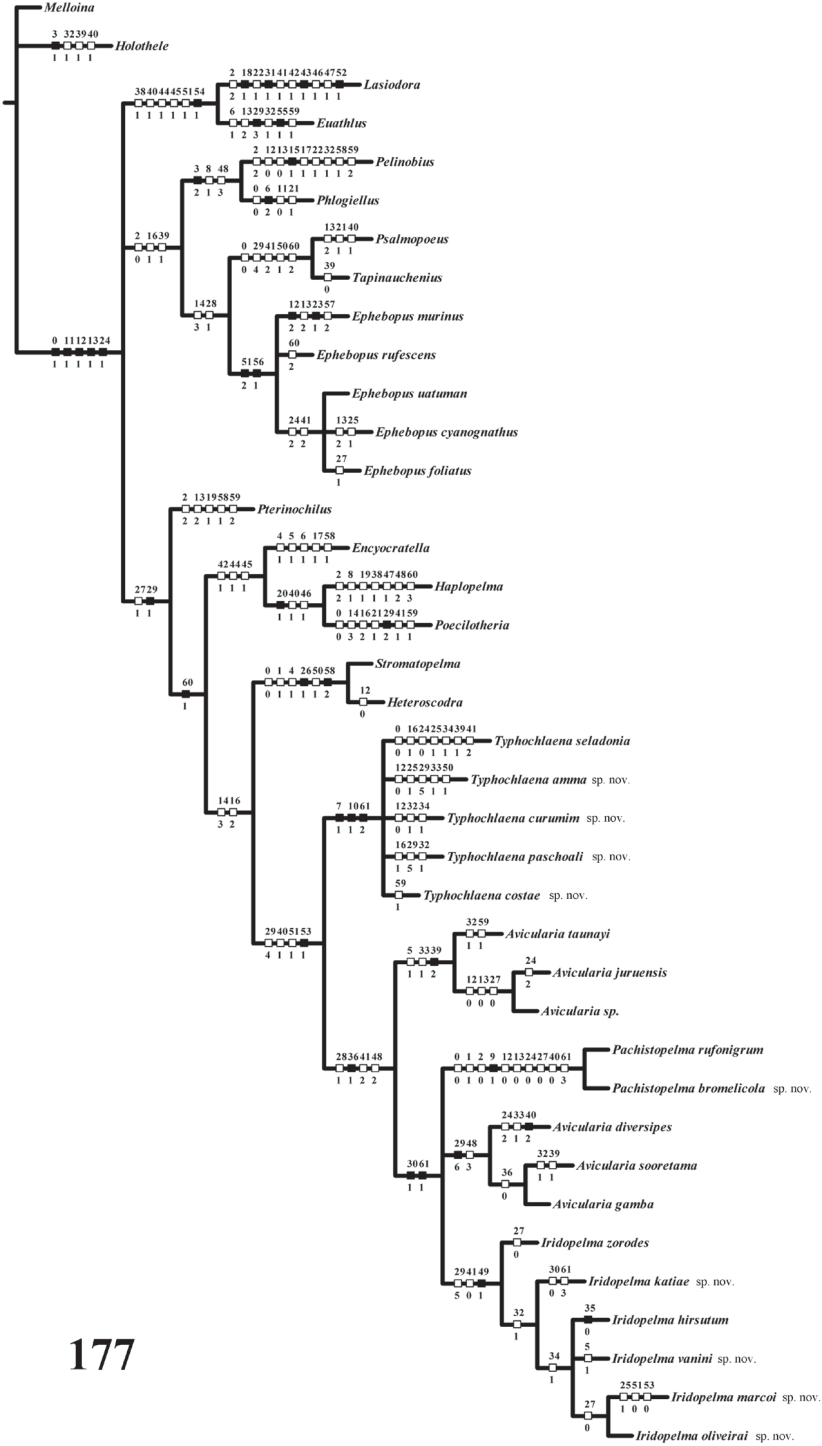
Strict consensus of 12 trees obtained with Nona and all characters non-additive. Black square = synapomorphy, white square = homoplasy. Length = 211, Ci = 0.44, Ri = 0.68.

**Figure 178.**
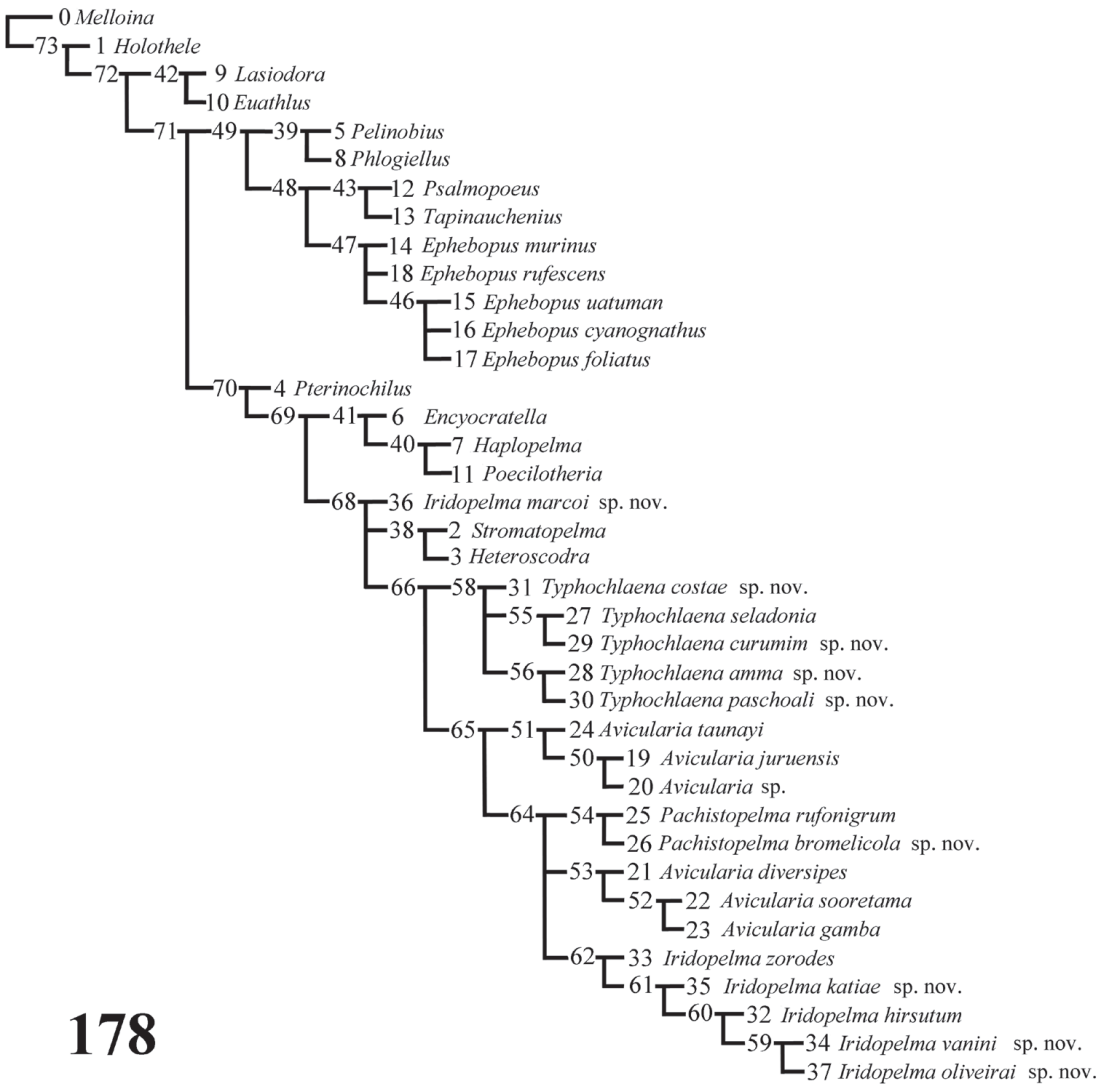
Single tree obtained with X-Pee-Wee, all characters non-additive and concavity 6. Fit = 4507.4 length = 209.

The results obtained herein were compared with other published analyses for *Ephebopus* spp. and Aviculariinae genera relationship of West et al. (2008), which share 21 taxa and 48 characters with the present work. NONA (Fig. 177) and X-PEE-WEE with concavities 2–6 (Fig. 178) results differed in topology with West et al. (2008) cladogram by clades having *Haplopelma* and *Poecilotheria* and that with *Psalmopoeus*, *Tapinauchenius* and *Ephebopus* spp. swapping positions. In West et al. (2008) these three genera were basal in the Aviculariinae and *Haplopelma* and *Poecilotheria* were sister to Theraphosinae. In the present analysis, Aviculariinae are paraphyletic, the clade *Encyocratella* (*Haplopelma* + *Poecilotheria*) is the sister-group of Aviculariinae and the clade with *Psalmopoeus*, *Tapinauchenius* and *Ephebopus* spp. is the sister group of *Pelinobius* + *Phlogiellus*. However, with X-PEE-WEE and concavity 1 Aviculariinae is retrieved as monophyletic, and, although with a slight different topology, it contains all genera proposed by West et al. (2008) to belong in Aviculariinae. Anyway, I refrain to make changes in the subfamily content as other analyses with all *Avicularia* species is in preparation and can give support for one or other topologies. Additional information on morphological variability of *Poecilotheria*, Ornithoctoninae, Selenocosmiinae and Eumenophorinae would help to resolve the conflict, but cladistic and taxonomical studies on these taxa are lacking.

In agreement with other studies (Ramírez 2003, West et al. 2008, Bertani et al. 2011) the shortest tree was obtained with X-PEE-WEE and concavity 6 (Fig. 178, Tables 3–4). It has also the highest fit (Table 2) and it is chosen as the preferred tree, on which the discussion below is done.

**Table 3.** Characters, Fits, Steps and Extra Steps for characters of cladogram of Fig 178.<br/>

**Character**	**Fit**	**Steps**	**Extra Steps**	**Character**	**Fit**	**Steps**	**Extra Steps**
0	50	7	6	31	100	2	0
1	66.7	4	3	32	42.86	9	8
2	54.5	7	5	33	75	3	2
3	100	2	0	34	75	3	2
4	85.71	2	1	35	100	2	0
5	60	5	4	36	85.71	2	1
6	85.71	3	1	37	100	1	0
7	100	1	0	38	85.71	2	1
8	85.71	2	1	39	60	6	4
9	100	1	0	40	54.54	7	5
10	100	1	0	41	54.54	7	5
11	85.71	2	1	42	85.71	2	1
12	50	8	6	43	85.71	3	1
13	40	11	9	44	85.71	2	1
14	75	5	2	45	85.71	2	1
15	-	-	-	46	85.71	2	1
16	66.67	5	3	47	85.71	2	1
17	85.71	2	1	48	66.67	6	3
18	-	-	-	49	100	1	0
19	85.71	2	1	50	75	3	2
20	100	1	0	51	85.71	3	1
21	75	3	2	52	-	-	-
22	85.71	2	1	53	100	1	0
23	-	-	-	54	100	1	0
24	60	6	4	55	-	-	-
25	66.67	4	3	56	100	1	0
26	100	1	0	57	85.71	3	1
27	50	7	6	58	75	4	2
28	85.71	2	1	59	50	9	6
29	75	8	2	60	75	5	2
30	85.71	2	1	61	85.71	4	1

**Table 4.** Synapomorphies for cladogram of Fig. 178.

**Taxa or Node**	**Character**	**Change**	**Taxa or Node**	**Character**	**Change**	**Taxa or Node**	**Character**	**Change**
*Holothele*	3	0 → 1	*Ephebopus cyanognathus*	13	1 → 2		29	0 → 4
	32	0 → 1		25	0 → 1		50	0 → 1
	39	0 → 1	*Ephebopus foliatus*	27	0 → 1		60	3 → 2
*Heteroscodra*	12	1 →0	*Ephebopus rufescens*	60	3 → 2	Node 46	24	1 → 2
*Pterinochilus*	2	1 → 2	*Avicularia* sp.1	24	1 → 2	Node 47	51	0 → 2
	13	1 → 2	*Avicularia diversipes*	24	1 → 2		56	0 → 1
	19	0 → 1		33	0 → 1	Node 48	14	2 → 3
	58	0 → 1		40	1 → 2		28	0 → 1
	59	0 → 2	*Avicularia sooretama*	32	0 → 1	Node 49	2	1 → 0
*Pelinobius*	2	0 → 2		39	0 → 1		16	0 → 1
	12	1 → 0	*Avicularia taunayi*	32	0 → 1		39	0 → 1
	13	1 → 0		59	0 → 1	Node 50	12	1 → 0
	15	0 → 1	*Typhochlaena seladonia*	0	1 → 0		13	1 → 0
	17	0 → 1		16	2 → 1		27	1 → 0 → 1
	22	0 → 1		24	1 → 0	Node 51	5	0 → 1
	32	0 → 1		25	0 → 1		33	0 → 1
	58	0 → 1	*Typhochlaena amma*	12	1 → 0		39	0 → 2
	59	0 → 2		25	0 → 1	Node 52	36	1 → 0
*Haplopelma*	2	1 → 2		33	0 → 1	Node 53	29	4 → 6
	8	0 → 1	*Typhochlaena curumim*	12	1 → 0	Node 54	0	1 → 0
	19	0 → 1		32	0 → 1		1	0 → 1 → 0
	38	0 → 1	*Typhochlaena paschoali*	16	2 → 1		2	1 → 0
	47	0 → 1		32	0 → 1		9	0 → 1
	48	3 → 2	*Typhochlaena costae*	59	0 → 1		12	1 → 0
	60	1 → 3	*Iridopelma hirsutum*	35	1 → 0		13	1 → 0
*Phlogiellus*	0	1 → 0	*Iridopelma vanini*	5	0 → 1		24	1 → 0
	6	0 → 2	*Iridopelma katiae*	30	1 → 0		40	1 → 0
	11	1 → 0		59	0 → 3		61	1 → 3
	21	0 → 1		61	1 → 3	Node 55	34	0 → 1
*Lasiodora*	2	1 → 2	*Iridopelma marcoi*	25	0 → 1	Node 56	29	4 → 5
	18	0 → 1		27	1 → 0	Node 58	7	0 → 1
	22	0 → 1		32	0 → 1		10	0 → 1
	31	0 → 1		34	0 → 1	Node 59	59	0 → 1
	41	0 → 1		59	0 → 1	Node 60	34	0 → 1
	42	0 → 1	*Iridopelma oliveirai*	27	1 → 0	Node 61	32	0 → 1
	43	0 → 1	*Encyocratella*	4	0 → 1	Node 62	29	4 → 5
	46	0 → 1		5	0 → 1		41	2 → 0
	47	0 → 1		6	0 → 1		49	0 → 1
	52	0 → 1		17	0 → 1	Node 64	30	0 → 1
*Euathlus*	6	0 → 1		58	0 → 1		61	0 → 1
	13	1 → 2	Node 38	0	1 → 0	Node 65	28	0 → 1
	29	0 → 3		1	0 → 1		36	0 → 1
	32	0 → 1		4	0 → 1	Node 66	29	1 → 4
	55	0 → 1		26	0 → 1		40	0 → 1
	59	0 → 1		50	0 → 1		51	0 → 1
*Poecilotheria*	0	1 → 0		58	0 → 2		53	0 → 1
	14	2 → 3	Node 39	3	0 → 2	Node 68	14	2 → 3
	16	0 → 2		8	0 → 1		16	0 → 2
	21	0 → 1	Node 40	20	0 → 1	Node 69	60	3 → 1
	29	1 → 2		40	0 → 1	Node 70	27	0 → 1
	41	0 → 1		46	0 → 1		29	0 → 1
	59	0 → 1	Node 41	42	0 → 1	Node 71	37	0 → 1
*Psalmopoeus*	13	1 → 2		44	0 → 1	Node 72	0	0 → 1
	21	0 → 1		45	0 → 1		11	0 → 1
	40	0 → 1	Node 42	38	0 → 1		12	0 → 1
*Tapinauchenius*	39	1 → 0		44	0 → 1		13	0 → 1
*Ephebopus murinus*	12	1 → 2		45	0 → 1		24	0 → 1
	13	1 → 2		51	0 → 1	Node 73	2	0 → 1
	23	0 → 1		54	0 → 1		60	0 → 3
	57	1 → 2	Node 43	0	1 → 0			

*Typhochlaena* is monophyletic and supported by characters sternum as long as wide, truncated behind (character 7, Table 4), and posterior lateral spinnerets with distal segment short, domed (character 10). The topology of *Typhochlaena* clade shows a tricotomy. In that *Typhochlaena seladonia* and *Typhochlaena curumim* sp. n. are sister-groups supported by spiraled spermathecae (character 34) with homoplasies in *Iridopelma marcoi* sp. n. and *Iridopelma hirsutum* (*Iridopelma vanini* sp. n. + *Iridopelma oliveirai* **sp. n**)**.** *Typhochlaena amma* sp. n. and *Typhochlaena paschoali* sp. n. are sister-groups supported by abdomen pattern in immatures (state 5 of character 29) with homoplasies with most *Iridopelma* species.

*Pachistopelma* is monophyletic and supported by 9 synapomorphies: low eye tubercle (character 0), straight anterior row of eyes (character 1), clypeus absent (character 2), flattened abdomen a quarter longer than wide in females (character 9), leg IV longer than leg I in females (character 12) and in males (character 13), leg rings on distal tibiae and metatarsi not evident (character 24), embolous straight or very slightly curved (character 40), bromelicolous lifestyle (61). All these synapomorphies are very homoplasious (3 additional steps or more), except for character 9, which is exclusive for the genus, and character 61, homoplastic with *Iridopelma katiae* sp. n.

*Iridopelma* is monophyletic only if *Iridopelma marcoi* sp. n. is excluded. However, some important characters for the analyses are exclusive to males (*i. e.*, tibial spurs, cymbium protuberance) and at least one, exclusive to *Iridopelma* (tibial spur presence on leg II). The lack of a known male in this species undoubtedly influenced its position in the cladogram topology. Also, the absence of urticating hairs in female, that is an important synapomorphy for a large aviculariine clade, surely contributed for this result as well. On the other hand, the spermathecae shape, as well as some somatic characters indicate it is an *Iridopelma* species. The discovery of the male of *Iridopelma marcoi* sp. n. migth confirm its position.

Other *Iridopelma* species are in a monophyletic clade. Three synapomorphies support *Iridopelma* monophyly: dorsal abdominal pattern in immatures (Character 29), homoplasious with *Typhochlaena amma* sp. n.; embolous length that changes from long to median (Character 41), extremally homoplasious, and presence of a tibial spur also on leg II, exclusive for the genus (character 49). Therefore, the main synapomorphy for *Iridopelma* is still the classical character proposed by Pocock (1901). However, the other proposed synapomorphy, leg I longer than leg IV (Pocock 1901) (character 12 for females and 13 for males) is extremely homoplasious. Even in *Iridopelma*, only *Iridopelma hirsutum* and *Iridopelma oliveirai* sp. n. males have a clearly longer leg I in relation to leg IV. All *Iridopelma* females have leg I and IV of similar length. Support for the node 59 (*Iridopelma vanini* sp. n.+ *Iridopelma oliveirai* sp. n.) is their habitat in deciduous forest (character 59), an homoplasious character. Node 60 *Iridopelma hirsurtum* (*Iridopelma vanini* sp. n. + *Iridopelma oliveirai* sp. n.) has as apomorphy the spiraled spermathecae (character 34). Node 61 is supported by character 32, presence of lobes in spermathecae, extremely homoplasious.

#### Biogeography.

**Figure 179.**
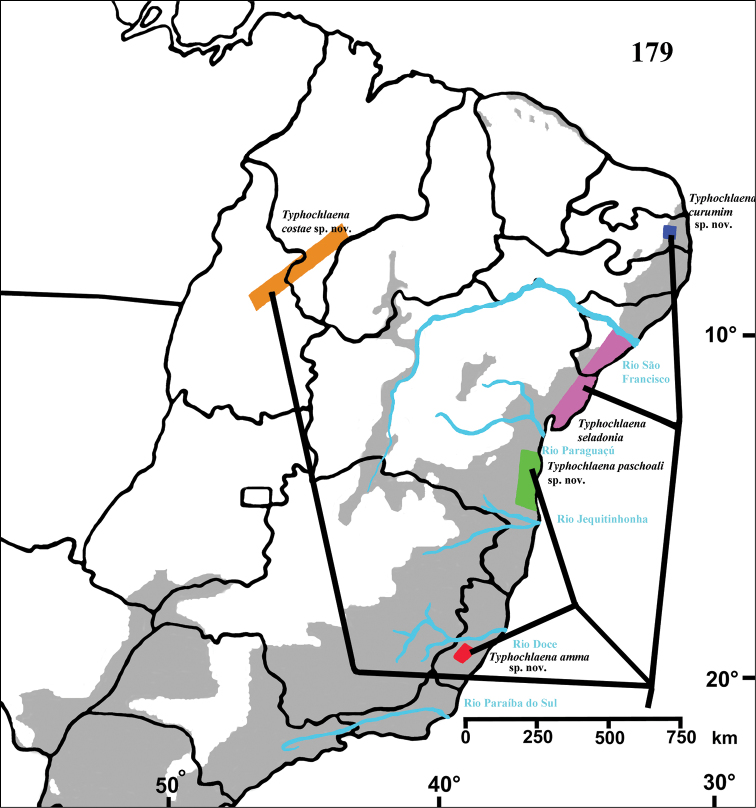
Area cladogram for *Typhochlaena* species in Brazil.

**Figure 180.**
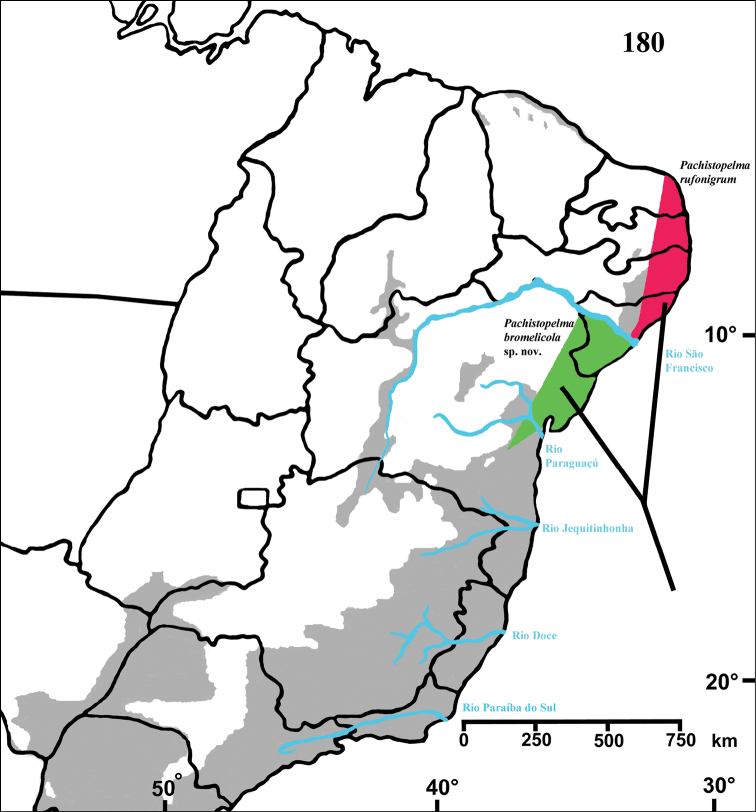
Area cladogram for *Pachistopelma* species in Brazil.

**Figure 181.**
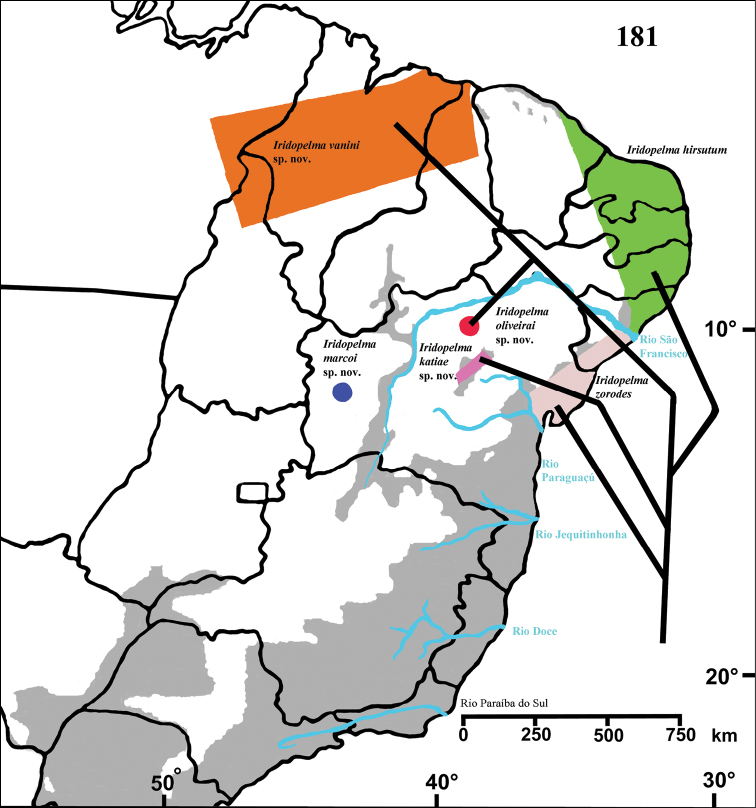
Area cladogram for *Iridopelma* species in Brazil.

**Figure 182.**
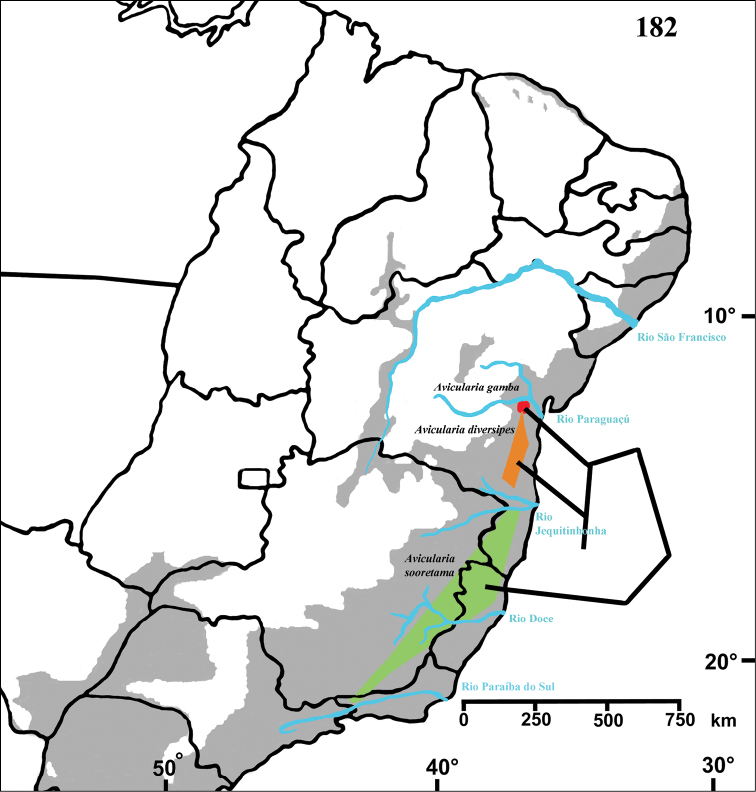
Area cladogram for *Avicularia* spp.2 (*Avicularia diversipes* (C. L. Koch, 1842), *Avicularia gamba* Bertani and Fukushima, 2009 and *Avicularia sooretama* Bertani and Fukushima, 2009) in Brazil.

*Typhochlaena*, *Pachistopelma* and *Iridopelma* species are highly endemic, and there is no overlapping in distribution of species belonging to the same genus (Figs 179–181). Most areas of endemism for species of those genera are mostly concordant with river systems, as proposed for Atlantic rainforest in Northeastern and Southeastern Brazil (Pellegrino et al. 2005). The northermost endemic area ranges roughly from state of Ceará/Rio Grande do Norte southwards to state of Alagoas and is limited in the south by Rio São Francisco. *Iridopelma hirsutum*, *Pachistopelma rufonigrum* and *Typhochlaena curumim* sp. n. distribution is restricted to this region (Figs 179–181). Southwards, another area of endemism is recognized between Rio São Francisco and Rio Paraguaçú (Pellegrino et al. 2005). *Iridopelma zorodes*, *Pachistopelma bromelicola* sp. n. and *Typhochlaena seladonia* are found in this endemic area, but there are some records for *Pachistopelma bromelicola* sp. n. south of Rio Paraguaçú (Elísio Medrado and Maracás, state of Bahia). Cladistic analysis retrieved *Typhochlaena seladonia*/*Typhochlaena curumim* sp. n. and *Pachistopelma rufonigrum*/*Pachistopelma bromelicola* sp. n. as sister species, indicating Rio São Francisco and Rio Paraguaçú as potential barriers responsible for vicariant event (Figs 179–180). However, the analysis did not retrieve *Iridopelma zorodes* as sister species of *Iridopelma hirsutum* (Fig. 181), suggesting a more complex history for *Iridopelma* genus in Northeastern and Center-Western Brazil. Another area of endemism was proposed for the region from Rio Paraguaçú southwards to Rio Jequitinhonha (Pellegrino et al. 2005). *Typhochlaena paschoali* sp. n., *Avicularia diversipes* and *Avicularia gamba* are endemic to the region (Figs 179, 182). Following southwards, another area of endemism is proposed between Rio Jequitinhonha and Rio Doce (Pellegrino et al. 2005). Only *Avicularia sooretama* is known from this region. Interestingly, Rio Doce is not a barrier for the species, which is distributed from Rio Jequitinhonha to Rio Paraíba do Sul (Fig. 182). However, there is a single old record for the species south of Rio Doce, in Itatiaia, State of Rio de Janeiro (Bertani and Fukushima 2009). The southermost area of endemism for aviculariines is between Rio Doce and Rio Paraíba do Sul. *Typhochlaena amma* sp. n. is endemic to this region and cladistic analysis retrieved it as sister species of *Typhochlaena paschoali* sp. n. (Fig. 179).

At least two hypotheses were proposed to explain the existence of endemism areas in Atlantic forest of Northeastern and Southeastern Brazil. Pellegrino et al. (2005) proposed that after a semiarid climate in Miocene/Pliocene boundary the region had higher humidity conditions in the Pliocene, and major rivers were formed, bissecting deposits from Barreiras Formation (Sugio and Nogueira 1999), thus isolating inter-riverine regions. The alternative hypothesis (Carnaval and Moritz 2008) provided evidence for the existence of forest refugia areas during the Quaternary that are congruent with areas of endemism known for Atlantic forest of Northeastern Brazil.

**Figure 183.**
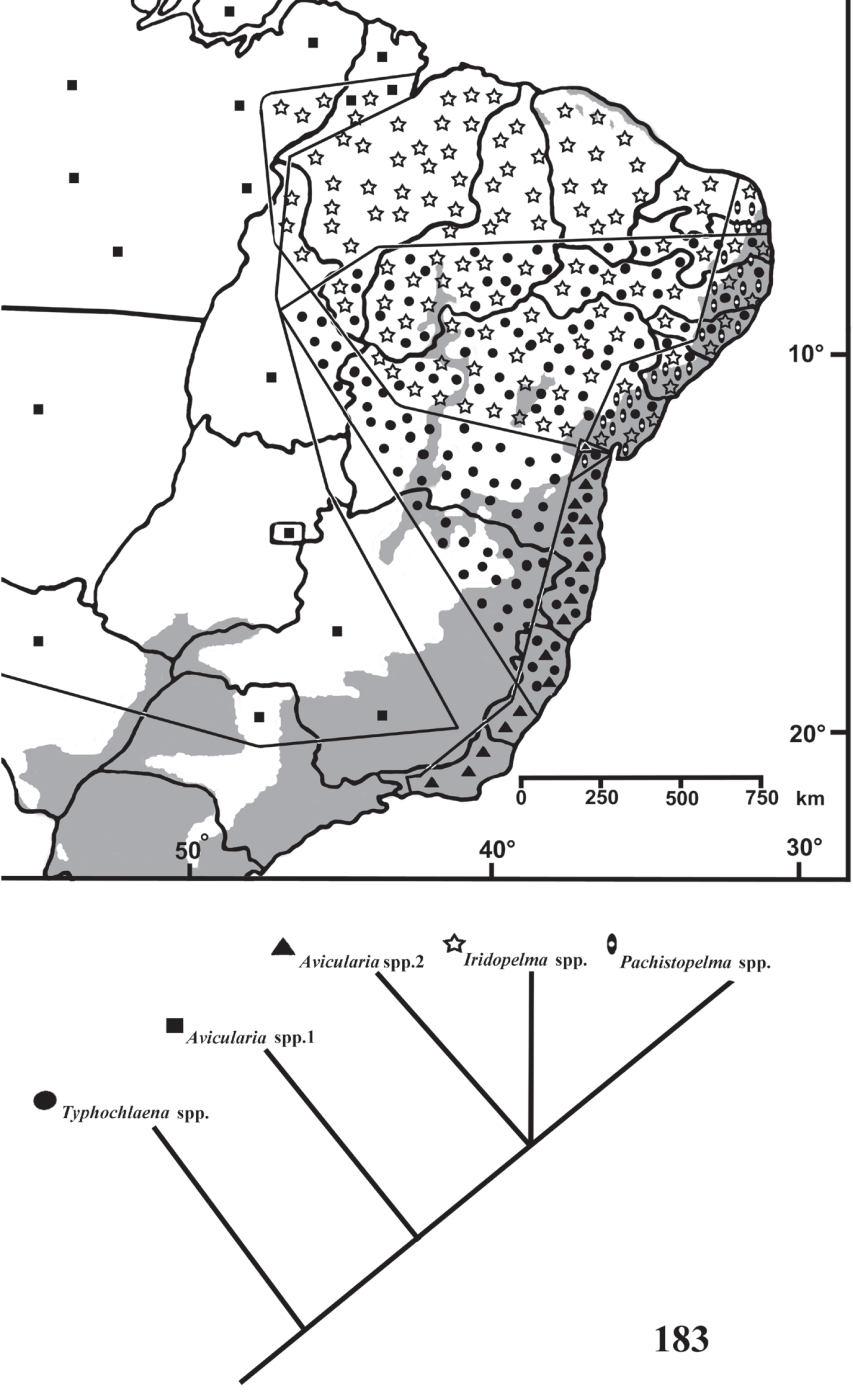
Area cladogram for *Avicularia* spp.1, *Avicularia* spp.2, *Iridopelma* spp., *Pachistopelma* spp. and *Typhochlaena* spp. in Brazil.

Whereas distribution of congeners did not overlap, there is strong ovelap of species of *Typhochlaena* with *Pachistopelma*, *Iridopelma* and *Avicularia* spp.2 as well as of *Iridopelma* with *Pachistopelma* (Fig. 183). *Typhochlaena* spp. overlap with *Avicularia* spp. 2 on state of Espírito Santo and Southern state of Bahia, with *Iridopelma* on Northern state of Bahia, Sergipe, Paraíba, Piauí, Maranhão and possibly Pernambuco, Alagoas and Tocantins. They also overlap with *Pachistopelma* spp. in Northern Bahia, Sergipe, Alagoas, Pernambuco and Paraíba. *Pachistopelma* spp. overlap with *Iridopelma* spp. and *Typhochlaena* spp. over almost all their distribution (from Rio Grande do Norte to Northern Bahia). *Iridopelma* possibly overlaps slightly with *Avicularia* spp.1 in parts of Maranhão, Pará and Tocantins, with *Typhochlaena* and *Pachistopelma* on the coast and with *Typhochlaena* on Northern Bahia, Sergipe, Alagoas, Pernambuco, Paraiba and Southern Ceara, Piauí, Maranhão and parts of Tocantins. Most of the overlap involves *Typhochlaena* – the most basal genus of the clade, and, if this genus is not considered, only a marginal overlapping of *Avicularia* spp.1, *Avicularia* spp.2 and *Iridopelma* will remain (except *Pachistopelma*, which will be discussed separately).

#### Habitat and evolution.

The overlap of *Iridopelma* spp. with *Pachistopelma* spp. in most of the distribution of the latter deserves a more detailed discussion. *Pachistopelma* spp. are strictly associated with bromeliads (Bertani et al. 1994, Santos et al. 2002, 2004; Dias et al. 2000, Dias and Brescovit 2003, Dias and Brescovit 2004, this work), a characteristic possibly shared only with *Iridopelma katiae* sp. n. In all other closely related aviculariine genera with available information, the retreat is made under loose tree bark (*Typhochlaena seladonia*, *Typhochlaena curumim* sp. n.), on tree trunk or on leaves of palm trees (*Avicularia* sp.1, *Avicularia juruensis* and *Avicularia taunayi*), with two or more leaves connected with silk threads (*Avicularia* spp. 2, *Iridopelma hirsutum*, *Iridopelma zorodes*). Therefore, almost all other aviculariine species use primarily trees to make their retreats, though some use bromeliads eventually (immature *Avicularia avicularia* - Stradling, 1994; *Iridopelma oliveirai* sp. n.– type label data; immature *Avicularia juruensis*, immature and adult *Avicularia diversipes* (pers. obs.).

*Pachistopelma* spp. inhabit tank bromeliads - *Aechmea aquilega* (Dias et al. 2000, Santos et al. 2004); *Hohenbergia stellata*, *Hohenbergia ridley* (Dias et al. 2000); *Hohenbergia ramageana* and *Avicularia lingulata* (Santos et al. 2004) which are mainly terrestrial in rocky outcrops, restinga and even caatinga, and are facultatively epiphytes (Siqueira Filho and Leme 2006). When terrestrials, they grow on areas of shallow, sandy or rocky soil, exposed to intense sunlight. Tank bromeliads retain rain water on their phytothemalta and are an important source of humidity to several species of animals (Frank and Lounibos 2009). Tank bromeliads are particularly abundant in drier areas of restinga which are depleted of high trees (Santos et al. 2004). In this xeric environment there are few available places with adequate humidity and protection against high temperatures. Therefore, bromeliads provide the few suitable microhabitats for a variety of animals.

*Pachistopelma* spp. seem specialized to live inside bromeliads, mainly by the dorso-ventrally flattened body of immatures and female which aids the spider in moving between the narrow inter-leaves spaces (Bertani 1994, Bertani in Dias et al. 2000). A question arising then is, why this specialization occurred with *Pachistopelma* spp., but not with *Iridopelma* spp. that live in contiguous areas? It is largely known that climate fluctuations of the Neogene and the Quaternary periods transformed large wet forested regions of Northeastern Brazil into xeric biomes (Ab’Sáber 1977, Martin et al. 1993, Suguio and Nogueira 1999, Carnaval and Moritz 2008). With the change, part of the fauna more dependent on wet and low temperature probably became extinct. Especially arboreal animals might be affected, as large trees became rare. On the other hand, bromeliads, that live normally as epiphytes or on rocks and forest borders, expanded their distribution on the ground. Thus, these plants were the few available places for the ancestor of *Pachistopelma* species to live. Natural selection then acted and morphological modifications took place leading to speciation and specialization to live strictly inside bromeliads. When climate changed and the region became wet again, the forest expanded and bromeliads became restricted as epiphytes or more concentrated in restinga regions in the coast, caatinga, or on rocky formations on hills. Even though the habitat along their distribution area is now suitable for an arboreal spider, the morphological, ethological end physiological specializations of *Pachistopelma* species did not allow them to recolonize trees. *Pachistopelma* populations seem to be smaller in bromeliads on shaded forest borders than in regions exposed to direct sunlight (Santos et al. 2004, pers. obs.). On the other hand, *Iridopelma* species expanded their distribution following forest expansion and now species of the two genera have sympatric distribution, one living inside bromeliads and the other in trees. Existence of other animal species living strictly inside bromeliads in the same region reinforces the idea. At least another spider, *Nothroctenus fuxico* Dias and Brescovit, 2004 is found exclusively in bromeliads, sometimes together with *Pachistopelma bromelicola* sp. n. specimens (Dias and Brescovit 2004). One scorpion, *Tityus neglectus* Mello-Leitão, 1932, is also found living inside tank bromeliads (Lourenço and Eickstedt 1988, Santos et al. 2003) in same regions as *Pachistopelma* spp. This model basically corresponds to the ecogeographical speciation proposed by Vanzolini and Williams (1981).

At least five other aviculariine species are found in open vegetation regions - cerrado (*Typhochlaena costae* sp. n., *Iridopelma marcoi* sp. n., *Iridopelma vanini* sp. n.), caatinga (*Iridopelma oliveirai* sp. n.), sandy dunes/restinga (*Iridopelma vanini* sp. n.), or campo rupestre (*Iridopelma katiae* sp. n.). For all species with available field data, there are records of them living on trees (*Iridopelma marcoi* sp. n.) or in bromeliads (*Iridopelma oliveirai* sp. n., *Iridopelma katiae* sp. n.). The holotype of *Iridopelma vanini* sp. n. was found under a fallen tree trunk on a sandy dune region in Parnaíba, state of Piaui. *Iridopelma katiae* sp. n. habitat is interesting, because it is in a high region (1200–1300 m a.s.l.) in a campo rupestre area. As restinga and caatinga, the climate is severe, with rocky formations, water stress present most times of year, high temperatures during the day and low temperatures at night, and few sparse trees (Conceição et al. 2007). Again, the only available place for an arboreal spider is the frequent bromeliad islands formed by *Vriesea atra*. The four specimens collected in this region were inside those bromeliads, including a female with spiderlings. The other two records are for a region close to this and the spiders were found under rocks, an unusual habitat for an arboreal spider. This indicates that *Iridopelma katiae* sp. n. could be suffering a similar selective pressure that led to the specialization to bromeliad lifestyle in *Pachistopelma* spp., after a climatic and vegetational change.

Another bromelicolous spider, the salticid *Psecas chapoda* (Peckham & Peckham, 1894), is specialized to living inside the bromeliad *Bromelia balanseae* (Romero and Vasconcelos-Neto 2005). These bromeliads also occur in open vegetation formation (cerrado), which is, sometimes, bordered by forest formations. *Psecas chapoda* is physiologically adapted to *Bromelia balanseae*, and denser populations’ occur far from shaded areas of forest borders (Romero and Vasconcelos-Neto 2005). A facultative mutualism involving *Psecas chapoda* and *Bromelia balanseae* has been proposed, in which the plant benefits from nutrients generated by spider debris, such as feces, exuvia, prey carcasses and silk (Romero et al. 2006, Romero et al. 2008). A similar process to that of *Pachistopelma* spp. could have occurred with an ancestor of this salticid spider, leading to a specialization to live inside bromeliads. Salticid association with bromeliads are much more frequent in bromelids of open environments, such as cerrado, rather than in bromeliads of forest interiors (Romero 2006). As with cerrado, campo rupestre is another key area to understanding the association between spiders and bromeliads. *Alpaida quadrilorata* (Simon, 1897) (Araneidae) is also associated with a bromeliad-like plant, *Paepalanthus bromelioides* (Eriocaulaceae) (Figueira and Vasconcellos-Neto 1991) which also has leaves in a rosette and occurs on sandy soil in campo rupestre areas of Serra do Cipó. I also observed the same association with these spider species and plant in campo rupestre of Caraça (Santa Bárbara, state of Minas Gerais). Therefore, this can be a recurrent process leading to the strict bromelicous lifestyle, and should be investigated in other animal groups having similar habits.

This complex history may be responsible for the extraordinary diversity of aviculariines in Northeastern, Southeastern and Central-western Brazil, which rivalizes with the aviculariine forms found in Northern South America and Central America in number of species, morphological variation and habitat use. Specifically, parts of Northeastern Brazil, as Reconcavo Bahiano, has one of the richest aviculariine fauna in the world, with records for *Avicularia diversipes*, *Avicularia gamba*, *Iridopelma zorodes*, *Pachistopelma bromelicola* sp. n., and possibly at least one *Typhochlaena* species.

Due to their limited dispersal abilities and high endemism, theraphosids are important taxa for biogeographic and evolutionary studies. Phylogeographical studies with species of aviculariines would aid in understanding important evolutionary processes and biogeographical patterns in South America, and I expect the present paper stimulates this kind of study with these animals.

#### Conservation.

As with other Brazilian Atlantic forest aviculariines (*Avicularia diversipes*, *Avicularia sooretama* and *Avicularia gamba* – Bertani and Fukushima 2009), species of *Typhochlaena*, *Iridopelma* and *Pachistopelma* are very endemic. *Typhochlaena* species are, moreover, rare and little is known about their habits. Only forty specimens are known in collections for all five species. As *Typhochlaena* is the sister group of a large aviculariine clade, the conservation of the relict species of this genus is imperative to preserve aviculariine diversity (Vane-Wright et al. 1991, Faith 1992).

*Iridopelma hirsutum* and *Iridopelma zorodes* are abundant, but they are known only from patches of Atlantic forest, which is limited to 7.91% of its original distribution (Fundação SOS Mata Atlantica and INPE 2009). These species depend on forest protection as a whole. Little data is available on *Iridopelma oliveirai* sp. n., *Iridopelma vanini* sp. n., *Iridopelma marcoi* sp. n. and *Iridopelma katiae* sp. n. All these species occur in drier areas in cerrado, caatinga, campo rupestre and restinga and can be under natural stress. Information on population sizes and habitat usage are desirable to aid in conservation of these species. *Pachistopelma* spp. populations are normally high, but their existence, as a specialized species, is dependent upon conservation of bromeliads, mainly in restinga of Northeastern Brazil. However, bromeliad species are also threatened in several portions of that region (Siqueira Filho and Tabarelli 2006).

## Supplementary Material

XML Treatment for Typhochlaena

XML Treatment for Typhochlaena seladonia

XML Treatment for Typhochlaena curumim

XML Treatment for Typhochlaena paschoali

XML Treatment for Typhochlaena amma

XML Treatment for Typhochlaena costae

XML Treatment for Pachistopelma

XML Treatment for Pachistopelma rufonigrum

XML Treatment for Pachistopelma bromelicola

XML Treatment for Iridopelma

XML Treatment for Iridopelma hirsutum

XML Treatment for Iridopelma zorodes

XML Treatment for Iridopelma vanini

XML Treatment for Iridopelma katiae

XML Treatment for Iridopelma oliveirai

XML Treatment for Iridopelma marcoi
